# An epigenome-wide view of osteoarthritis in primary tissues

**DOI:** 10.1016/j.ajhg.2022.05.010

**Published:** 2022-06-08

**Authors:** Peter Kreitmaier, Matthew Suderman, Lorraine Southam, Rodrigo Coutinho de Almeida, Konstantinos Hatzikotoulas, Ingrid Meulenbelt, Julia Steinberg, Caroline L. Relton, J. Mark Wilkinson, Eleftheria Zeggini

**Affiliations:** 1 Institute of Translational Genomics, Helmholtz Zentrum München, German Research Center for Environmental Health, 85764 Neuherberg, Germany; 2 Graduate School of Experimental Medicine, TUM School of Medicine, Technical University of Munich, 81675 Munich, Germany; 3 MRC Integrative Epidemiology Unit, Population Health Sciences, University of Bristol, Bristol BS8 2BN, UK; 4 Department of Biomedical Data Sciences, Section Molecular Epidemiology, Leiden University Medical Center, 2333 ZC Leiden, the Netherlands; 5 The Daffodil Centre, The University of Sydney, a Joint Venture with Cancer Council NSW, Sydney, NSW 1340, Australia; 6 Department of Oncology and Metabolism, The University of Sheffield, Sheffield S10 2RX, UK; 7 TUM School of Medicine, Technical University of Munich and Klinikum Rechts der Isar, 81675 Munich, Germany

**Keywords:** DNA methylation, osteoarthritis, cartilage, synovium, methylation QTL, chondrocyte, synoviocyte, machine learning, EWAS

## Abstract

Osteoarthritis is a complex degenerative joint disease. Here, we investigate matched genotype and methylation profiles of primary chondrocytes from macroscopically intact (low-grade) and degraded (high-grade) osteoarthritis cartilage and from synoviocytes collected from 98 osteoarthritis-affected individuals undergoing knee replacement surgery. We perform an epigenome-wide association study of knee cartilage degeneration and report robustly replicating methylation markers, which reveal an etiologic mechanism linked to the migration of epithelial cells. Using machine learning, we derive methylation models of cartilage degeneration, which we validate with 82% accuracy in independent data. We report a genome-wide methylation quantitative trait locus (mQTL) map of articular cartilage and synovium and identify 18 disease-grade-specific mQTLs in osteoarthritis cartilage. We resolve osteoarthritis GWAS loci through causal inference and colocalization analyses and decipher the epigenetic mechanisms that mediate the effect of genotype on disease risk. Together, our findings provide enhanced insights into epigenetic mechanisms underlying osteoarthritis in primary tissues.

## Introduction

Osteoarthritis (MIM: 165720) is a complex degenerative joint disease characterized by chronic pain and stiffness. It affects more than 40% of people over the age of 70 and is a leading cause of disability worldwide.^1^ In spite of its high prevalence, treatment methods are limited to pain management and total joint replacement (TJR). To drive the development of novel and personalized treatments, it is necessary to understand the genetic and genomic architecture underlying osteoarthritis. Genome-wide association studies (GWASs) have determined around 150 independent osteoarthritis-linked single-nucleotide variants.^2^ For the most part, it is unknown which variants and genes at these loci are causal to disease development and along which molecular pathways they exert their osteoarthritis-promoting effect. To identify these mechanisms, studies using relevant tissues are necessary, and TJR surgeries provide an opportunity to molecularly profile relevant tissues from osteoarthritis-affected individuals.^3^

DNA methylation in promoter regions and particularly around the transcription start site is strongly associated with gene downregulation, whereas its effect in gene bodies or other regulatory regions remains less predictable. DNA methylation is dynamic, with highly tissue-specific patterns,^4^ and can interact with a multitude of factors such as genotype, age, sex, or environment.^5^ The methylation profiles of relevant tissues and cell types in complex diseases can further our understanding of disease etiology, for example by generating insights into perturbed regulatory mechanisms and by revealing epigenetic markers of disease development or progression. Given the importance of tissue-specific molecular patterns, initiatives such as GTEx,^6^ ENCODE,^7^ ROADMAP,^8^ and BLUEPRINT^9^ have generated large publicly available resources that have made molecular datasets broadly accessible. However, these datasets do not include osteoarthritis-affected tissues.

To fill this gap, a small number of studies have investigated DNA methylation profiles of articular cartilage, typically comparing methylation profiles between macroscopically intact (low-grade) and degraded (high-grade) osteoarthritis cartilage to identify epigenetic markers of cartilage degeneration. Previous epigenome-wide association studies (EWASs) of this type have been limited in size, with a maximum of 17 knee osteoarthritis-affected individuals studied to date.^10^, ^11^, ^12^, ^13^, ^14^ There is a need for better powered studies to improve our understanding of the role of DNA methylation in osteoarthritis (supplemental note S1).

Combining DNA methylation data with matched genotypes enables the detection of genetic variants associated with differential methylation levels at cytosine-guanine dinucleotides (CpGs), i.e., methylation quantitative trait loci (mQTLs). Characterizing these associations can help elucidate effector genes through which disease-associated genetic risk variants may exert their biological effect. To date, studies seeking to investigate mQTL effects in joint tissues have mostly focused on a candidate gene^15^ or single genetic variants previously linked to osteoarthritis.^16^, ^17^, ^18^, ^19^ One study has investigated the association of genome-wide methylation with gene expression in osteoarthritis-affected cartilage in 31 osteoarthritis-affected individuals (17 knee and 14 hip osteoarthritis patients).^11^ They reported 87 methylation sites that were correlated with the expression of 70 genes, where both gene and methylation site were linked to cartilage degeneration. Of these, 36 were targeted by *cis*-mQTLs. There remains a need to comprehensively map the mQTL landscape on a genome-wide scale and, in better-powered sample sizes, to generate comprehensive insights into the interplay between genetic variation and epigenetic changes in osteoarthritis tissues, and to provide a resource to help elucidate the mechanism for novel genetic risk loci discovered in GWASs.

To date, molecular studies of osteoarthritis have mainly focused on articular cartilage, the most prominent osteoarthritis-affected tissue. However, osteoarthritis is regarded as a disease of the whole joint, affecting multiple tissues within the synovial joint. Therefore, expanding genomic analyses to include other joint tissues has the potential to reveal novel insights into disease progression. The synovium, a connective tissue that lines the joint capsule separating the synovial cavity from neighboring tissues, undergoes pathological alterations during osteoarthritis. There is well-documented evidence of synovial inflammation in osteoarthritis-affected joints, referred to as synovitis.^20^ Several studies have compared mQTL effects across tissues but have focused only on specific, osteoarthritis-linked loci.^16^^,^^19^^,^^21^^,^^22^

In this study, we have analyzed genome-wide methylation profiles from up to 98 osteoarthritis-affected individuals undergoing TJR due to knee osteoarthritis (matched low-grade and high-grade cartilage and synovium). We enhance our understanding of osteoarthritis aetiopathogenesis by (1) identifying methylation markers for cartilage degeneration, (2) building machine-learning-based models to distinguish between low-grade and high-grade osteoarthritis cartilage samples, (3) determining genome-wide methylation quantitative trait loci (mQTLs) in osteoarthritis tissues (cartilage and synovium), and (4) resolving high-confidence effector genes for osteoarthritis GWAS signals.

## Subjects and methods

For full details of methods, see supplemental subjects and methods.

### Osteoarthritis-affected individuals and study samples

Samples from osteoarthritis-affected knees were collected in 101 osteoarthritis-affected individuals that underwent total knee replacement due to late-stage osteoarthritis. Cartilage samples were graded with the OARSI cartilage classification system (cohort1) or International Cartilage Repair Society (ICRS) scoring system (cohort2 and cohort3). This work was approved by Oxford NHS REC C (10/H0606/20 and 15/SC/0132), and samples were collected under Human Tissue Authority license 12182, Sheffield Musculoskeletal Biobank, University of Sheffield, UK. Before participating in the study, all osteoarthritis-affected individuals provided written, informed consent.

### Sample extraction

A previous study^3^ reported the isolation of the chondrocytes (section “Isolation of chondrocytes”), the isolation of synoviocytes (section “Isolation of synoviocytes”), and DNA extraction (section “DNA, RNA and protein extraction”) in its methods part.

### DNA methylation data

Genome-wide DNA methylation was measured with the Illumina 450k or EPIC array in three sequencing batches. We used the R package minfi to read idat files.^23^^,^^24^ We removed samples of three ethnicity outliers, gender mismatches (two samples), X-Y ratio outliers, and samples with unbalanced ratios between methylated and unmethylated signals (ten samples). To normalize methylation signals, we applied functional normalization,^25^ We removed probes on sex chromosomes, probes with detection p values of p > 0.01 in more than 5% of the samples, and previously reported cross-reactive probes.^26^, ^27^, ^28^ Furthermore, we excluded probes that had been reported to overlap with common genetic variants, as the signal of these probes might solely reflect genetic variation rather than true methylation signal.^26^ The resulting data comprised 401,870 methylation loci and 266 samples from 98 osteoarthritis-affected individuals (56 female and 42 male patients, age range: 38–88, age mean: 69.6, age sd: 9.72, Table S1), including 98, 90, and 78 samples from low-grade osteoarthritis cartilage, high-grade osteoarthritis cartilage, and synovium, respectively. We conducted downstream statistical analyses on M values as recommended.^29^

### DNA methylation data (replication set)

We used published methylation data for low-grade and high-grade osteoarthritis cartilage to replicate the findings of the EWAS and the machine-learning-based classifiers.^30^

The data is publicly available in the Gene Expression Omnibus database^31^ and accessible through the entry number GEO: GSE63106*.* The replication data comprises methylation data of matching low-grade and high-grade osteoarthritis cartilage samples from 31 patients who underwent total joint replacement to treat primary osteoarthritis (knee: 17 osteoarthritis-affected individuals, hip: 14 osteoarthritis-affected individuals).

### Genotype data

Genotypes were measured with the InfiniumCoreExome-12v1-1_A array or the InfiniumCoreExome-24v1-1_A array (supplemental subjects and methods). Genotype data were preprocessed as previously described.^3^

### Sample stratification with multivariate modelling

To investigate differences between tissues on a global level, we used DNA methylation data (including 98, 90, and 78 samples from low-grade and high-grade osteoarthritis cartilage and synovium, respectively) corrected for batch effects with the ComBat function^32^ from the R package sva and considered these corrected methylation. We applied (1) principal-component analysis (R function prcomp) and (2) a follow-up hierarchical clustering (R package FactoMineR).^33^

### Differential methylation analysis (discovery)

To identify differentially methylated sites (DMSs) in pairs of low-grade and high-grade osteoarthritis cartilage samples from 90 osteoarthritis-affected individuals, we performed linear modeling by using the function lmFit and eBayes function of limma.^34^ We added the factor variable patient ID to ensure paired analysis design and 18 surrogate variables (SVs) to account for technical confounders as covariates. To assess genome-wide significance in the EWAS, we applied Bonferroni correction considering the number of tested methylation sites: 0.05/401,870 = 1.24 × 10^−7^. To identify differentially methylated regions (DMRs), we applied the R package dmrff.^35^ Regions were defined as differentially methylated when composed of more than one methylation site and achieving a Bonferroni-adjusted p < 0.05. To identify sex-specific markers of cartilage degeneration, we used a similar approach as in the combined analysis (supplemental subjects and methods).

### Differential methylation analysis (replication)

We performed an EWAS on knee samples of the replication data (17 low-grade and high-grade osteoarthritis cartilage samples, respectively) to validate our findings. To determine DMSs between low- and high-grade osteoarthritis cartilage, we applied a mixed-effect model, which is similar to what has been applied to this dataset previously^11^ (supplemental subjects and methods). Replicated DMSs are defined as (1) showing the same direction of effect in the replication set (2) at nominal significance (p < 0.05). We performed the EWAS on a regional level in the replication dataset with dmrff (default settings analog to the discovery analysis). We defined DMRs as replicated when they are composed of exactly the same methylation sites in the replication set and show the same direction of effect on nominal significance.

### Pathway enrichment analysis

We used the gometh and goregion functions (available through R package missMethyl) to identify enrichments among DMSs and DMRs.^36^^,^^37^ We considered pathways consisting of between 20 and 200 genes.

### Distinguishing cartilage grades with machine learning

We constructed classifiers that distinguish cartilage grades. More specifically, we trained and tested random forest (RF)-based classifiers repeatedly in 5-fold cross validations (cv) in 25 iterations (R package caret). In total, we trained and tested 125 RF models (25 iterations × 5-fold cv) (supplemental subjects and methods). To validate our approach, we trained RF-, support-vector-machine-, and gradient-boosting-machine-based classifiers on our entire dataset and tested the prediction quality of the resulting classifiers on the validation dataset. We then applied the classifiers and assessed their prediction quality separately in hip and knee samples. Prediction accuracies and their 95% confidence intervals were calculated with carets ConfusionMatrix-function.

### Identification of methylation quantitative trait loci

We performed genome-wide *cis*-methylation quantitative trait locus (mQTL) analysis in low-grade (97 samples) and high-grade osteoarthritis cartilage (89 samples) as well as in synovium (78 samples), thus including only samples for which complete covariate information was available. We restricted our analyses to SNPs with a minor allele frequency > 0.05. Furthermore, we defined the *cis*-distance with 1 Mb. We conducted the mQTL analysis by using the R package MatrixEQTL.^38^ We applied linear models and corrected for age, sex, and batch effects (supplemental subjects and methods). We defined two thresholds to identify genome-wide-significant methylation QTL effects.

(1) Bonferroni threshold: genome-wide significance defined by p < 0.05/number of tested SNP-methylation site pairs (low-grade osteoarthritis cartilage: p < 3.05 × 10^−11^, high-grade osteoarthritis cartilage: p < 3.03 × 10^−11^, synovium: p < 3.03 × 10^−11^).
(2) False discovery rate (FDR): we estimated the FDR of mQTL effects by using the MatrixEQTL package. It calculates the FDR considering the total number of tested *cis*-pairs per tissue.

To characterize mQTL architecture in osteoarthritis tissues, we used methylation site annotations of Illumina’s annotation file (version 1.2). For the enrichment approaches, we applied hypergeometric tests (R function phyper). To identify sex-specific *cis*-mQTLs, we applied MatrixEQTL by using an interaction model (supplemental subjects and methods).

### Differential mQTL effects in low-grade and high-grade osteoarthritis cartilage

To calculate differential mQTL effects between low-grade and high-grade osteoarthritis cartilage, we used the software MetaTissue v0.5 (see web resources).^39^ Analogously to our genome-wide, tissue-specific approach to identify mQTLs, we included sex, age, and sequencing batches as covariates in these models. We used the MetaTissue software to calculate posterior probabilities (m values) and focused on genetic variant-methylation site pairs with a significant effect in one tissue (m value > 0.9) but not in the other (m value < 0.1).

### Comparing joint with whole blood methylation QTLs

We compared mQTL effects (Bonferroni correction) of joint tissues (low-grade osteoarthritis cartilage, high-grade osteoarthritis cartilage, and synovium) with the corresponding effects (mQTL effect between the same variant-methylation site pairs) of a mQTL meta-analysis (Genetics of DNA Methylation Consortium, see web resources) of 36 cohorts in whole blood.^40^ We considered results from the fixed-effect models from the whole blood mQTL meta-analysis.

### Summary statistics of GWASs

For the MR approach and the colocalization analysis, we included summary statistics from three osteoarthritis-related phenotypes: (1) osteoarthritis at any site (all OA) and (2) knee osteoarthritis (knee OA) and (3) total knee replacement (TKR). Summary statistics for all OA and knee OA were previously published^41^ and downloaded from the GWAS Catalog. We calculated summary statistics for TKR by meta-analyzing the arcOGEN and UKBB data with the METAL software.^42^

### Two-sample Mendelian randomization

To estimate putative causal effects of methylation, we applied two-sample Mendelian randomization (2SMR) by integrating mQTL data of the three examined joint tissues and GWAS data from three osteoarthritis traits (all OA, knee OA, and TKR). We performed 2SMR following the workflow implemented in the R package TwoSampleMR (version 0.4.25).^43^ In low-grade osteoarthritis cartilage, we tested 3,378 methylation sites for their putative causal effect on osteoarthritis (all OA = 3,378 methylation sites, knee OA = 3,378, and TKR = 3,343). In high-grade osteoarthritis cartilage, we considered 2,042 methylation sites (all OA = 2,042, knee OA = 2,042, and TKR = 2,026). In synovium, we investigated the effect of 1,561 methylation sites (all OA = 1,560, knee OA = 1,560, and TKR = 1,542). In total, we tested 10,099, 6,110, and 4,662 methylation site-osteoarthritis trait combinations in low-grade and high-grade osteoarthritis cartilage and synovium, respectively. Per tissue, we applied the Bonferroni method to correct for the number of performed tests (low-grade osteoarthritis cartilage: p < 4.95 × 10^−6^, high-grade osteoarthritis cartilage: p < 8.18 × 10^−6^, synovium: p < 1.07 × 10^−5^).

We investigated the opposite direction of effect (osteoarthritis causal for methylation changes) for every tested methylation site-osteoarthritis trait combination (using R package TwoSampleMR). We used 27, 10, and 4 SNPs as instrumental variable (IV) for all OA, knee OA, and TKR, respectively. Here, we applied the inverse-variance-weighted (IVW) method.

### Colocalization analysis

We applied colocalization analysis to statistically estimate the overlap of mQTL signals in the three osteoarthritis tissues and GWAS signals.^44^ We examined genome-wide signals for osteoarthritis at any site (all OA, 33 risk loci), knee osteoarthritis (knee OA, 12 risk loci), and total knee replacement (TKR, 5 risk loci). We performed colocalization by applying coloc.fast function (web resources). We conducted the colocalization analysis separately for each GWAS osteoarthritis trait and each tissue (supplemental subjects and methods). We used a posterior probability threshold for having a shared causal variant (“PP4”) of ≥80% (thus indicating colocalization) as previously applied.^3^ Annotated genes and locations of colocalized GWAS signals were extracted from Ensembl Variant Effect Predictor (see web resources).

### Combining colocalization results with eQTL and gene expression data

We combined these colocalization results with previously estimated eQTL data from the same patient cohort.^3^ More specifically, we tested whether the lead SNP of colocalized GWAS OA signals show an eQTL effect on local genes at nominal significance (p < 0.05). We used previously published, matching expression data (low-grade osteoarthritis cartilage: 75 osteoarthritis-affected individuals, high-grade osteoarthritis cartilage: 76, synovium: 70)^3^ of the same osteoarthritis-affected individuals in the same tissue types to test associations between osteoarthritis-linked methylation sites and genes in the same region. We estimated associations between methylation and gene expression by using linear models. We estimated putative causal effects of methylation on gene expression by using one-sample MR with the R package ivreg (supplemental subjects and methods).

### Comparative analysis of colocalization in joint and blood

We tested whether osteoarthritis-risk variant-methylation site pairs that colocalize using joint mQTL data also colocalize when overlapping osteoarthritis GWASs with whole blood mQTL data. For this colocalization approach, we applied the same colocalization method as performed on joint mQTL data. We applied a threshold of PP4 ≥ 80% and PP4 < 20% indicating colocalization and no colocalization, respectively.

## Results

### Methylation profiles differ between tissue types and disease grades

**Figure 1:**
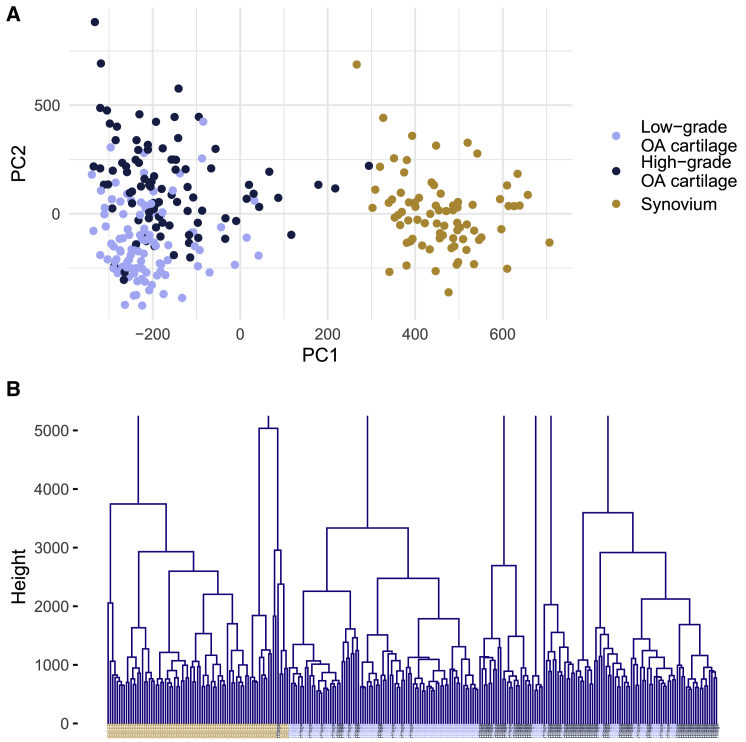
Multivariate analyses of methylation profiles distinguish between different tissues and disease grades (A) In a principal-component analysis, the first PC separates cartilage from synovium, while the second PC is associated with cartilage grades (with overlapping clusters from low-grade and high-grade osteoarthritis cartilage samples). (B) Hierarchical clustering shows a separation of global methylation profiles by tissue type. “Height” on the y axis denotes the distance between clusters. OA denotes osteoarthritis.

To describe distinct methylation profiles in three tissue types (low-grade and high-grade osteoarthritis cartilage and synovium), we first assessed whether tissue and osteoarthritis grade have strong, systematic effects on global variations in the epigenome. We used principal-component analysis to examine variation in global methylation profiles and observed a clear separation between synovium and cartilage samples along the first principal component (PC) and partly overlapping clustering between low-grade and high-grade osteoarthritis cartilage along the second PC (Figure 1A). A linear model confirmed the significant association between the second PC and cartilage grades (p = 1.51 × 10^−^^16^, beta = 253.79, SE = 27.93). Using a hierarchical clustering approach, we observed stratification by tissue type and cartilage degradation state (Figure 1B).

### EWAS reveals widespread, robustly replicating signals

**Figure 2:**
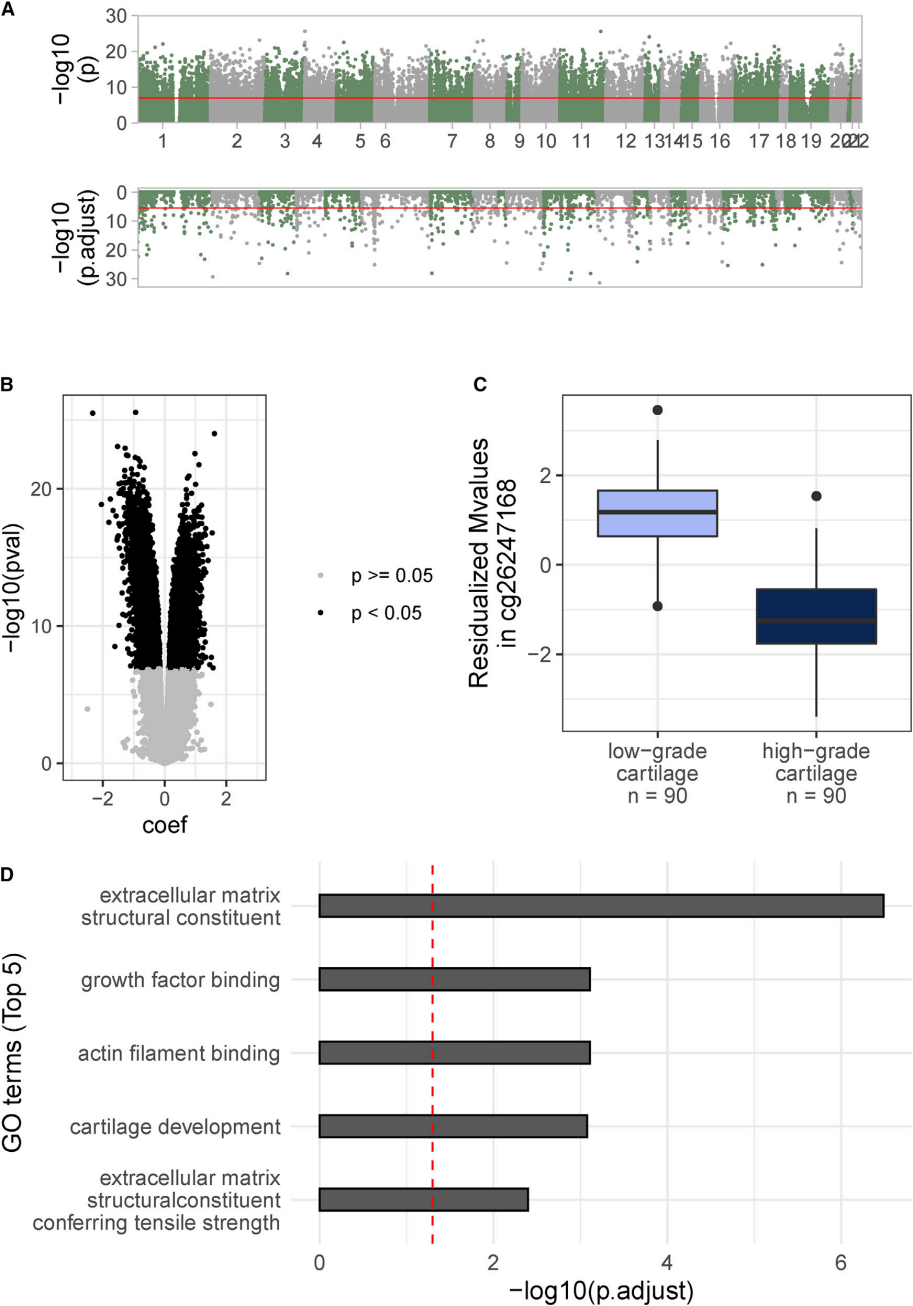
Differential methylation between low-grade and high-grade osteoarthritis cartilage (A) Genome-wide signals for differential methylation sites (top) and regions (bottom) between low-grade and high-grade osteoarthritis cartilage. Red lines indicate genome-wide significance (top: nominal p < 1.24 × 10^−7^, bottom: Bonferroni-adjusted p < 0.05). (B) Volcano plot showing hyper- and hypomethylated sites. (C) An example of hypomethylation in high-grade osteoarthritis cartilage at cg26247168 (beta: −2.32, p = 3.05 × 10^−26^, SE = 0.14). The boxplots represent 25^th^, 50^th^, and 75^th^ percentiles, and whiskers extend to 1.5 times the interquartile range. (D) Most significant Gene Ontology gene annotations enriched in 15,328 DMSs. Red dashed lines indicate the significance threshold (Benjamini-Hochberg-adjusted p < 0.05).

To identify DNA methylation markers of cartilage degeneration, we performed an EWAS on paired low-grade and high-grade osteoarthritis cartilage samples from 90 osteoarthritis-affected individuals across 401,870 methylation sites (supplemental note S2). We identified 15,328 differentially methylated sites (DMSs) distributed across the whole genome (Figures 2A [upper panel], 2B, and 2C and Table S2) by using a significance threshold of p < 1.24 × 10^−7^ (subjects and methods). Furthermore, we identified 2,477 differentially (Bonferroni-adjusted p < 0.05) methylated regions (DMRs) (Figure 2A [bottom panel], Figure S1, and Table S3).

To biologically characterize the DMS, we performed enrichment analyses and identified 29 and 4 Gene Ontology (GO) and Kyoto Encyclopedia of Genes and Genomes (KEGG) terms, respectively (Figure 2D, Figure S2, and Table S4), including pathways linked to osteoarthritis, e.g., terms associated with external matrix organization^45^ and skeletal system development,^10^^,^^13^^,^^14^^,^^45^ as well as the epithelium-related term “positive regulation of epithelial cell migration” in articular cartilage. This term showed limited overlap with other enriched pathways on the constituent gene level (e.g., extracellular matrix structural constituent: two of 93 annotated, differentially methylated genes are also annotated to “positive regulation of epithelial cell migration,” collagen fibril organization: one of 34, integrin-mediated signaling pathway: 11 of 58, cartilage development: seven of 104, chondrocyte differentiation: five of 62) suggesting its distinctness, e.g., to pathways that are linked to the extracellular matrix or cartilage development. This pathway may point to an epithelium-related etiological mechanism.

**Figure 3:**
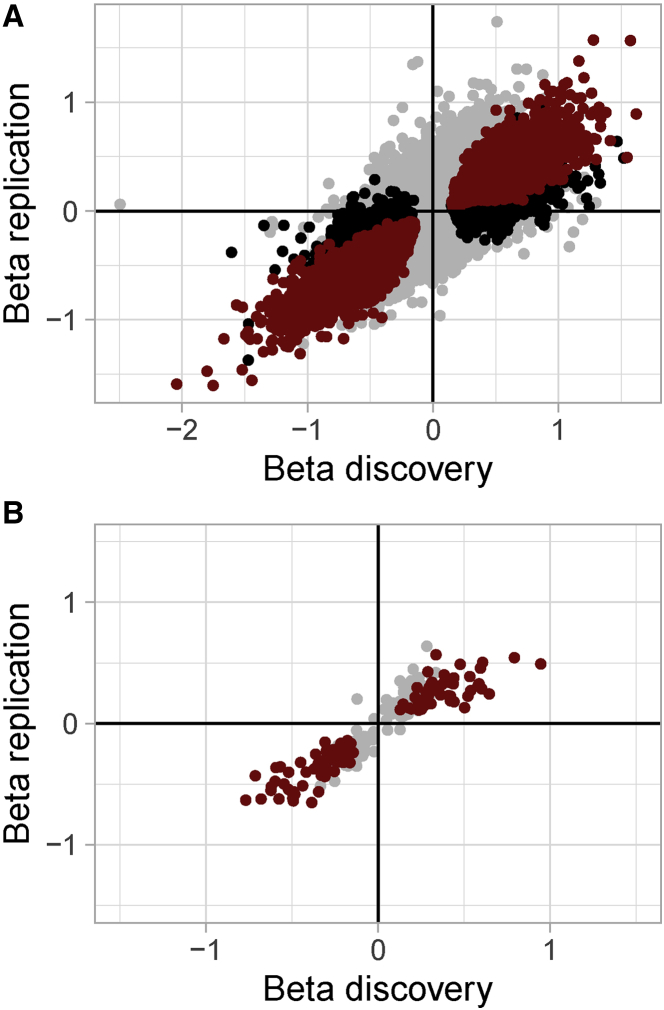
Replication of EWAS results in an independent dataset (A and B) Effects of (A) methylation sites (n = 346,288) and (B) methylation regions (n = 271) present in both of the discovery and replication datasets. Black dots refer to DMSs/DMRs of the discovery set, red dots to DMSs/DMRs that additionally show an effect at nominal significance (nominal p < 0.05) in concordant direction in the replication set.

We used an independent dataset from 17 knee osteoarthritis patients to replicate the epigenetic differences between low-grade and high-grade osteoarthritis cartilage.^11^^,^^30^ We replicated 7,192 DMSs and 105 DMRs (Tables S2, S3, and S5). The effect sizes of replicated DMSs (Pearson r = 0.96, p < 2.2 × 10^−16^) and DMRs (Pearson r = 0.95, p value < 2.2 × 10^−16^) in the discovery and replication datasets were highly correlated (Figures 3A and 3B). These results point to the robustness of the identified methylation changes.

We further performed EWAS separately on paired low-grade and high-grade cartilage in female (n = 52) and male (n = 38) osteoarthritis-affected individuals and identified female- (n = 1,338) and male- (n = 3,316) specific DMSs in cartilage, suggesting sex-specific markers (supplemental note S2).

### Machine-learning models distinguish cartilage grades with high accuracy

Next, we sought to test whether epigenetic changes in different cartilage grades can be harnessed to develop a model that robustly distinguishes low-grade from high-grade osteoarthritis cartilage. First, we constructed RF-based classifiers in the discovery knee osteoarthritis-affected individual cohort in a repeated 5-fold cross-validation approach. Here, we achieved high prediction accuracies (mean accuracy: 90.69%; standard deviation: 4.08, 95% confidence interval [CI] 89.98–91.41). Furthermore, the resulting receiver operating characteristic (ROC) curve revealed an area under the curve of 0.97 (Figure S3), highlighting the high sensitivity and specificity of these classifiers.

To validate these findings, we trained the final RF-based classifier on our entire patient cohort (subjects and methods) and evaluated its accuracy in an external dataset composed of 17 knee and 14 hip osteoarthritis-affected individuals.^30^ In this replication cohort, we achieved an accuracy of 82.35% (95% CI 65.47–93.24) for knee samples, whereas in hip samples the achieved accuracy was lower at 64.29% (95% CI 44.07–81.36). We also observed these differences when using support vector machines (knee: 85.29%, 95% CI 68.94–95.05; hip: 57.14%, 95% CI 37.18–75.54) and gradient-boosting machines (knee: 76.47%, 95% CI 58.83–89.25; hip: 50.00%, 95% CI 30.65–69.35). The lower accuracy achieved in hip samples supports the effect of methylation joint specificity within osteoarthritis.^30^^,^^45^ GO enrichment analysis of the 300 most important methylation sites in the final RF model did not identify any significant enrichments. Of these 300 methylation sites, 99.3% (n = 298) and 77.7% (n = 233) were among the DMS identified in the discovery and replication analysis, respectively. This suggests that epigenetic markers for cartilage degeneration are prioritized predictors in the classifier. External validation of the classifier was somewhat limited by the small sample size of the replication set, resulting in wide confidence intervals. Hence, validation in larger datasets is further warranted.

This model shows that epigenetic differences can be used to distinguish disease stages in cartilage. Samples of more accessible tissue types (such as blood and synovial fluid) need to be included in the model training and testing to develop a clinically relevant tool.

### Genome-wide mQTL maps in osteoarthritis-relevant tissues

**Figure 4:**
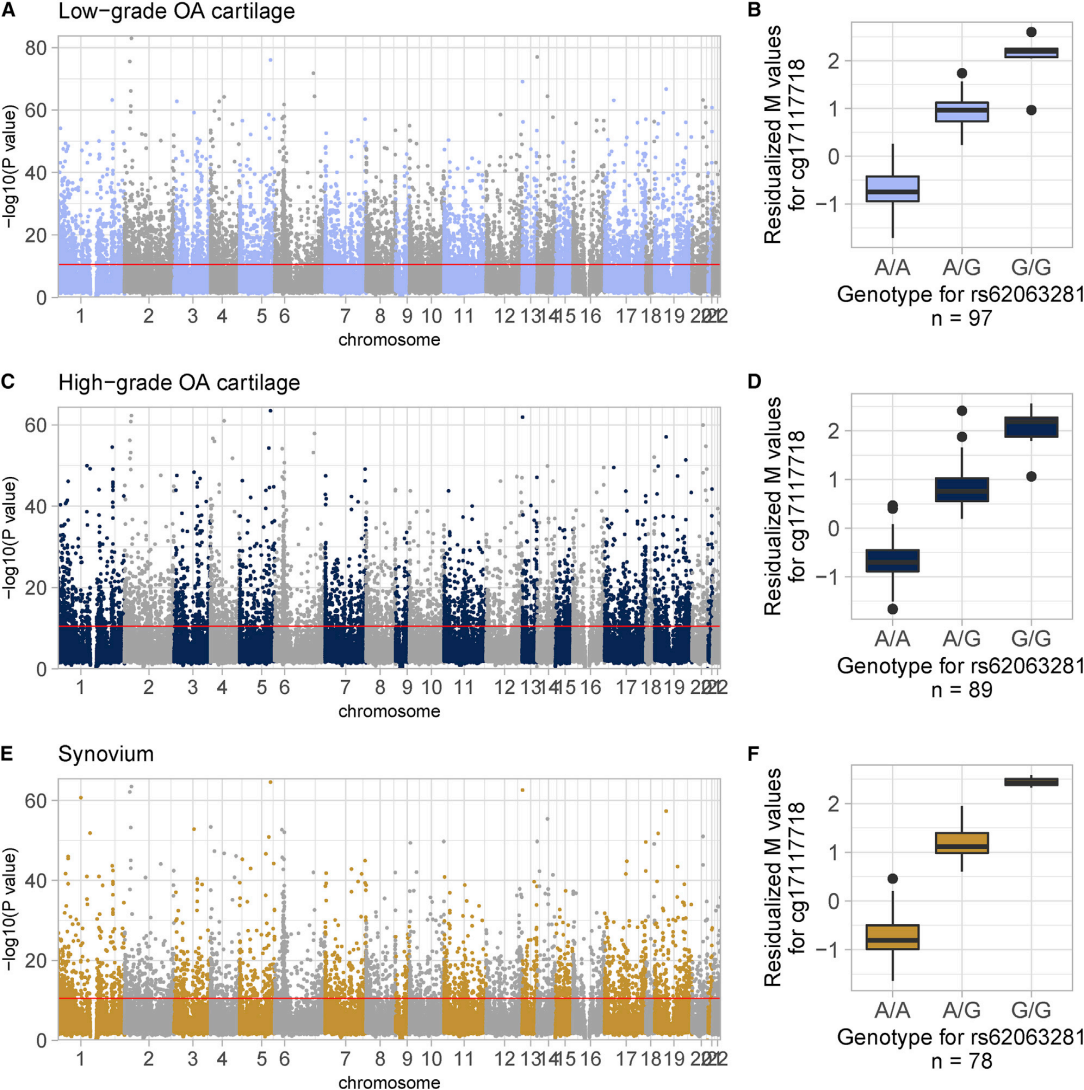
The mQTL landscape in cartilage and synovium (A–F) Manhattan plots depicting the negative log of the p value of the most significant association per methylation site across all variants within 1 Mb in (A) low-grade osteoarthritis cartilage, (C) high-grade osteoarthritis cartilage, and (E) synovium. Red lines indicate genome-wide significance (Bonferroni correction). The boxplots describe the effect of rs62063281 on methylation site cg17117718 in (B) low-grade osteoarthritis cartilage (beta = 1.65, p = 1.19 × 10^−48^, SE = 0.06), (D) high-grade osteoarthritis cartilage (beta = 1.60, p = 3.55 × 10^−37^, SE = 0.07), and (F) synovium (beta = 1.95, p = 2.39 × 10^−44^, SE = 0.06), as an example. The boxplots represent 25^th^, 50^th^, and 75^th^ percentiles, and whiskers extend to 1.5 times the interquartile range.

We combined DNA methylation data with matching genotype data from the same osteoarthritis-affected individuals to identify genetic variants that are significantly associated with methylation levels of proximal methylation sites (<1 Mb; *cis*-mQTLs). We performed this analysis at the genome-wide scale in low-grade (n = 97) and high-grade (n = 89) osteoarthritis cartilage samples as well as in synovium (n = 78 samples), and identified widespread signal in every tissue (Figure 4, Figure S4, and Table S6). Applying a conservative Bonferroni threshold to correct for the number of tested genetic variant-methylation site pairs per tissue (p < 1 × 10^−11^), we identified 10,639, 6,785, and 4,493 methylation sites significantly associated with at least one mQTL in low-grade osteoarthritis cartilage, high-grade osteoarthritis cartilage, and synovium, respectively, and also included genetic-epigenetic effects in loci of previously reported mQTLs in osteoarthritis-relevant tissue (supplemental note S3). This represents a genome-wide map of mQTLs in osteoarthritis tissues. These data are made publicly available (see data and code availability).

Next, we further characterized the architecture of these mQTL maps. In low-grade and high-grade osteoarthritis cartilage, 66.93% (7,121 of 10,639) and 66.44% (4,508 of 6,785) methylation sites with at least one mQTL were annotated to a gene, respectively (Figures S5 and S6). In both cartilage tissue types, we identified significant (Bonferroni p < 0.05) over-representation of intergenic methylation sites or sites within gene bodies and under-representation of methylation sites close to transcription start sites (“TSS200,” “TSS1500”), in untranslated regions (“3' UTR,” “5' UTR”) and first exons (“1^st^ exon”).

In synovium, 67.44% (n = 3,030 of 4,493) methylation sites with at least one mQTL were annotated to a gene (Figure S7). Here, we found significant (Bonferroni p < 0.05) over-representation of intergenic methylation sites and under-representation of methylation sites that are within 200 bp to a transcription start site or in untranslated regions or first exons. These results suggest similar mQTL architectures across osteoarthritis tissues.

Furthermore, we tested whether mQTL effects differ between osteoarthritis-affected individuals of different sexes and identified methylation sites targeted by sex-specific mQTLs (FDR < 0.05) in low-grade (n = 282) and high-grade (n = 337) osteoarthritis cartilage as well as in synovium (n = 874) (Figure S8 and Tables S7, S8, and S9). This suggests sex-specific genetic effects on methylation in osteoarthritis tissues.

### Comparing mQTLs in cartilage and synovium with whole blood

Next, we asked whether the mQTL profiles of primary osteoarthritis tissues differ to those of more easily accessible, peripheral tissue samples. We compared the *cis*-mQTL effects of each of the three examined joint tissues with those of whole blood, which is the most commonly examined tissue type for DNA methylation. To maximize the number of identifiable osteoarthritis-tissue-specific effects, we compared *cis*-mQTL effects in joint-tissue to those of a publicly available, large-scale whole blood meta-analysis including 36 studies (27,750 European ancestry participants).^40^

Because a mQTL can be associated with more than one methylation site (and vice versa), we use the term “mQTL-site pair” to indicate the association between a specific mQTL and a specific methylation site. Of the 482,751 mQTL-site pairs in low-grade osteoarthritis cartilage, information of 365,411 were available in whole blood. Of these, 88.6% (n = 323,863) were significant in blood (p < 10^−11^) with a concordant direction of effect. Notably, 9.61% of overlapping mQTL-site pairs (n = 35,117) were both significant and had an opposite direction of effect in blood. Similarly, we compared 219,661 (of 286,558) mQTL-sites pair identified in high-grade osteoarthritis cartilage and found that 90.53% (n = 198,867) of these had a significant (p < 10^−11^) effect in the same direction in blood. Notably, 7.87% (n = 17,297) of present mQTL-site pair had a significant but opposing direction of effect in blood. In synovium, 78.88% (n = 156,931) of 198,958 mQTL-site pair were available in whole blood, of which 96.87% (n = 152,023) had a significant mQTL effect in blood (p < 10^−11^) in the same direction. 2.29% (n = 3,594) of overlapping mQTL-site pairs showed a significant effect in the opposite direction in blood. In summary, we found that the majority of mQTL effects identified in osteoarthritis-related tissues show the same direction in whole blood but also observed effects in opposing directions in all tested joint tissue types. The latter indicates non-negligible differences in the mQTL profile between osteoarthritis-relevant joint tissues and whole blood.

### Identification of grade-specific mQTLs in cartilage

We compared mQTL effects between the osteoarthritis joint tissues. Comparing low-grade cartilage with synovium, we found 143,258 mQTL-site pairs to be significant in both. The effects showed high correlation (Pearson r = 0.97, p < 10^−16^) and 33 mQTL-site pairs showed an effect in the opposite direction. The effects of the 122,378 mQTL-site pairs that were significant in both high-grade cartilage and synovium also showed high correlation (Pearson r = 0.97, p < 2.2 × 10^−16^), and their effect directions were all concordant. In low-grade and high-grade osteoarthritis cartilage, the effect sizes of mQTL-site pairs were highly correlated (comparing 256,036 mQTL-site pairs that were significant in both low-grade and high-grade osteoarthritis cartilage: Pearson r = 0.99, p < 2.2 × 10^−16^) and showed only concordant effect directions. Overall, our findings point to broadly concordant mQTL effects across osteoarthritis tissues.

**Figure 5:**
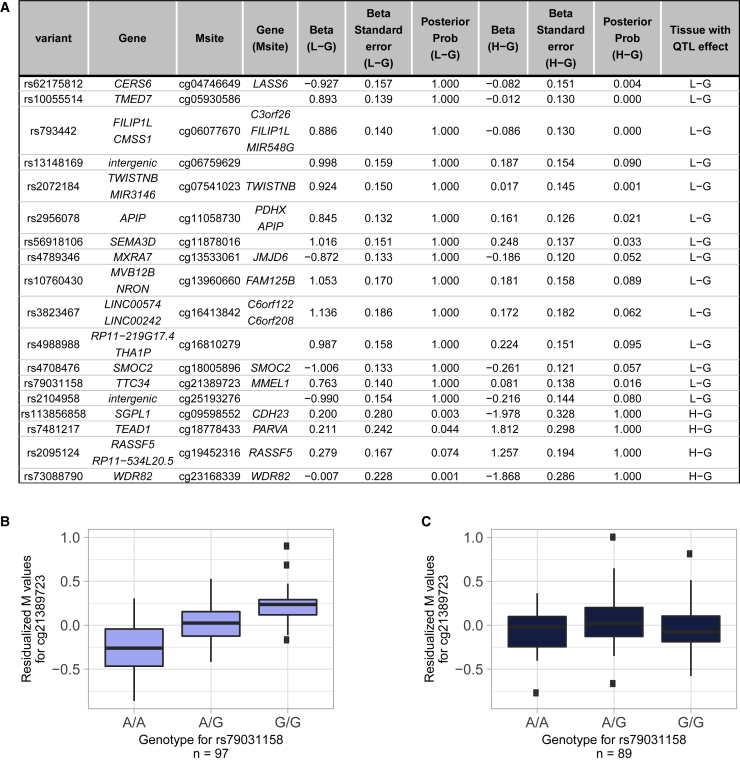
Differential mQTLs (A–C) Each row refers to a variant with a differential mQTL effect. The table reports the genetic variant and the targeted methylation site as well as annotated genes, effect sizes with corresponding standard errors, and posterior probabilities (>0.9 indicate an effect, <0.1 indicate no effect) for the effects in low-grade or high-grade osteoarthritis cartilage. The reported effects were estimated by a meta-analysis approach (subjects and methods). Boxplots (B) and (C) exemplify a differential mQTL: rs79031158 is associated with methylation of cg21389723 in low-grade (B) but not in high-grade osteoarthritis cartilage (C). The boxplots represent 25^th^, 50^th^, and 75^th^ percentiles, and whiskers extend to 1.5 times the interquartile range. Msite, methylation site; L-G, low-grade osteoarthritis cartilage; H-G, high-grade osteoarthritis cartilage; Posterior Prob, posterior probability.

We subsequently sought to identify differential mQTLs, i.e., mQTLs that are present in either low-grade or high-grade cartilage but not in both. This can help identify mQTL effects that are potentially “switched on/off” with increasing cartilage degeneration grade, i.e., with disease stage. To this end, we applied a meta-analysis approach,^39^ which improves power in identifying differential mQTLs by estimating a posterior probability of >0.9 and <0.1 indicating the presence and absence of a mQTL effect, respectively. In total, we identified 195 genetic variants that show a differential mQTL effect on 18 methylation sites (Table S10). Following clumping, one independent differential mQTL was retained per methylation site (Figure 5). Of the 18 targeted methylation sites, 14 and 4 were mQTLs in low-grade and high-grade osteoarthritis cartilage only, respectively. Genes annotated to these methylation sites are linked to osteoarthritis-relevant terms in cartilage, e.g., they encode a matrix metalloproteinase (*MMEL1*) or are involved in cell adhesion (*CDH23* and *PARVA*).

### Assessing the causal role of methylation in osteoarthritis

To identify methylation sites that play a causal role in osteoarthritis progression, we applied two-sample Mendelian randomization (MR) to the methylation sites associated with cartilage degeneration (exposure) and the mQTLs we identified in osteoarthritis-relevant tissues, together with genetic associations from three GWASs: knee osteoarthritis (knee OA), osteoarthritis at any site (all OA), and total knee replacement (TKR). We used the mQTLs as instrumental variables (Figure S9) in the MR analysis.

We identified 6, 8, and 11 significant osteoarthritis trait-methylation site combinations in low-grade and high-grade osteoarthritis cartilage and synovium, respectively. When performing an MR approach to examine causality in the opposite direction, namely the effect of osteoarthritis on methylation (Figure S10), we could not find any evidence for a significant effect for these osteoarthritis trait-methylation site combinations, thus providing further evidence for the causal role of these methylation sites on osteoarthritis (and not vice versa).

**Figure 6:**
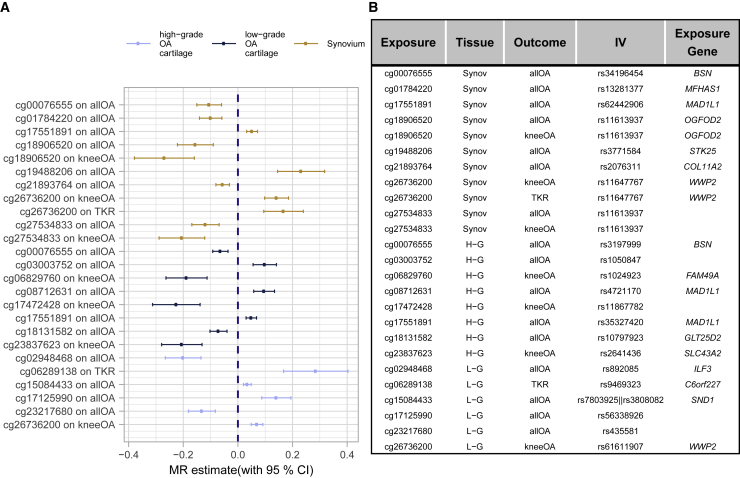
Overview of putative causal effects of methylation on osteoarthritis-related traits (A and B) Forest plot (A) describing the putative causal effect (with 95% confidence interval) of increasing methylation levels in the respective sites on osteoarthritis-related traits. Only significant exposure-outcome associations exceeding tissue-specific Bonferroni thresholds are reported (low-grade osteoarthritis cartilage: p < 4.95 × 10^−6^, high-grade osteoarthritis cartilage: p < 8.18 × 10^−6^, synovium: p < 1.05 × 10^−5^). The table (B) reports the instrumental variable(s) (IV[s]) and annotated genes. We applied the Wald-ratio test in cases of one IV; otherwise the inverse-variance-weighted method was applied.

In total, we identified 19 methylation sites with a putative causal effect on osteoarthritis (Figure 6 and Table S11). In low-grade osteoarthritis cartilage, we identified six methylation sites with a potential causal effect (Bonferroni correction, p < 4.95 × 10^−6^). Four of these showed association with hypermethylation, and two showed associations with hypomethylation and osteoarthritis development. Among the annotated genes is *WWP2* (cg26736200 in gene body), a key regulator in chondrocytes (discussion).

In high-grade cartilage, eight methylation sites were causally linked to osteoarthritis (Bonferroni correction, p < 8.18 × 10^−6^). Of these, five sites showed association of hypermethylation with a protective effect against osteoarthritis development, whereas the other three sites were associated with higher risk. Annotated genes include *COLGALT2* (cg18131582 in gene body), a transferase that catalyzes the transfer of galactose to collagen during collagen synthesis.^46^ A previous study suggests that the expression of this gene in cartilage is influenced by an osteoarthritis-risk variant.^47^

In synovium, we identified 11 significant methylation site-trait combinations, involving eight unique methylation sites (p < 1.07 × 10^−5^). In five of these eight sites, increased methylation levels showed a protective effect against osteoarthritis development, whereas in three sites hypermethylation was associated with higher risk. Annotated genes include *MFHAS1* (cg01784220 in the 1st exon), a gene involved in Toll-like receptor signaling,^48^^,^^49^ which is thought to be centrally involved in the osteoarthritis-related immune response in synovial joints.^50^

We identified one methylation site (cg26736200) in low-grade osteoarthritis cartilage and two methylation sites (cg17551891 and cg00076555) in high-grade osteoarthritis cartilage that were also identified as potentially causal for osteoarthritis in the synovium. For these three methylation sites, the direction of effect was concordant across tissues. Cg26736200 is annotated to the gene body of *WWP2*. Cg1755189 is located in the gene body of *MAD1L1,* a gene involved in cell-cycle regulation, which may point to cell senescence of chondrocytes in osteoarthritis articular cartilage.^51^ Cg00076555 is located in the 3′ UTR of *BSN* (discussion).

### Resolution of GWAS signals

**Figure 7:**
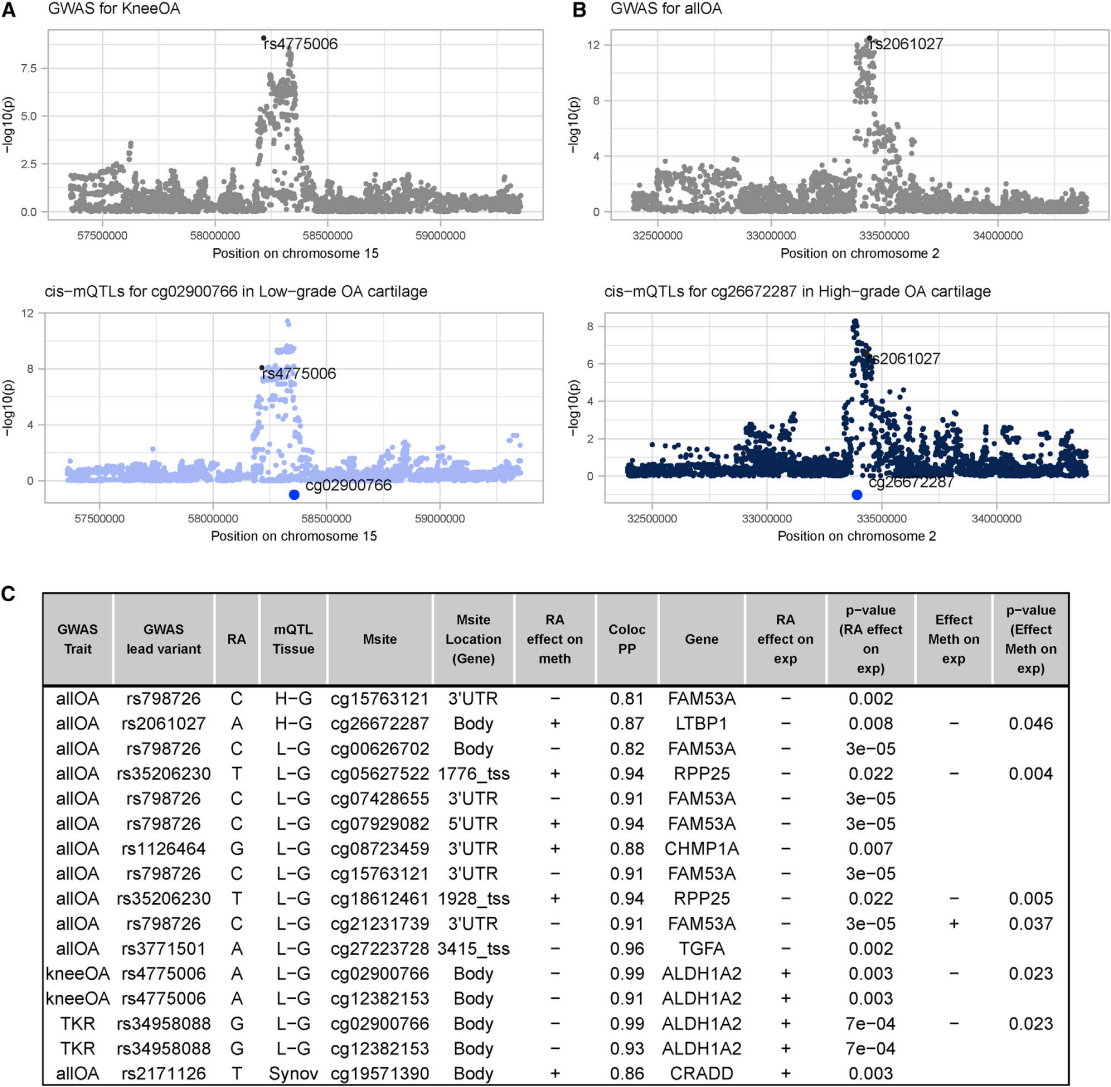
Colocalization reveals overlapping signals in GWAS and mQTL data (A–C) (A) and (B) exemplify colocalization events. In (A), we colocalized signals of *cis*-mQTL for the methylation site cg02900766 (A, bottom) with the GWAS for knee OA in the same genomic region (A, top). Here, we observed a posterior probability (PP) for a shared causal variant of 98.6%. Similarly, (B) visualizes the colocalization (PP = 86.5%) of *cis*-mQTL signals targeting cg26672287 in high-grade osteoarthritis cartilage (bottom) with GWAS signals for all OA (top). The highlighted variant (black) refers to the GWAS index variant in the respective genetic locus. (C) outlines osteoarthritis-linked genetic variants that colocalize with a methylation site and additionally show an eQTL effect at nominal significance (nominal p < 0.05) on the gene annotated to the respective methylation site in the same tissue. For four genes, we also identified an association (at nominal significance) with methylation sites for which *cis*-mQTLs, in turn, colocalize with a GWAS signal. RA, risk allele; Msite, methylation site; Coloc PP, posterior probability for colocalization; exp, gene expression; meth, methylation; _tss (in column Msite Location), methylation sites that are close to a transcript start site of the respective gene. The preceding number refers to the distance in bp.

We performed a colocalization analysis to determine whether osteoarthritis-linked genetic risk variants exert their effect through the regulation of nearby methylation sites. For all OA, 13 of 33 tested GWAS signals colocalized with mQTLs (ten in low-grade osteoarthritis cartilage, seven in high-grade osteoarthritis cartilage, and six in synovium; example in Figure 7B). For knee OA, six of 12 tested GWAS signals colocalized with mQTL signals (five in low-grade osteoarthritis cartilage, four in high-grade osteoarthritis cartilage, and four in synovium; example in Figure 7A). For TKR, one of five tested GWAS signals colocalized with mQTL signals (in low-grade osteoarthritis cartilage). Overall, osteoarthritis-related GWAS signals colocalized with mQTL signals of 32 unique methylation sites in low-grade osteoarthritis cartilage, 29 in high-grade osteoarthritis cartilage, and 17 in synovium. In total, we colocalized mQTL signals of 56 unique methylation sites with osteoarthritis-risk variants across the three affected individual tissues (Table S12).

By comparing the findings from colocalization and causal inference analysis (in the previous section), we identified two methylation sites in low-grade osteoarthritis cartilage (cg17125990 and cg26736200) and one methylation site in synovium (cg26736200) across both approaches, providing further evidence that these methylation sites play a causal role in osteoarthritis in the respective joint tissue.

Next, we combined these findings with results from eQTL data^3^ generated in the same patient cohort. When osteoarthritis GWAS signals colocalized with mQTL data, we tested whether the GWAS signal index variant exerted an effect on the expression levels of any gene close to the relevant methylation site. We found such an eQTL effect below nominal significance levels for five genes in low-grade osteoarthritis cartilage (*ALDH1A2*, *CHMP1A*, *FAM53A*, *RPP25*, and *TGFA*), two genes in high-grade osteoarthritis cartilage (*FAM53A* and *LTBP1*), and one gene in synovium (*CRADD*) (Figure 7C). In total, we identified seven genes linked to an osteoarthritis-risk locus. Given their link to osteoarthritis-risk variants across two molecular layers, these genes are high-confidence effector genes at these osteoarthritis GWAS loci in the respective tissue.

We compared these results with findings from a recent differential expression analysis.^3^ Two high-confidence effector genes were shown to be differentially expressed in high-grade compared to low-grade osteoarthritis cartilage (in high-grade osteoarthritis cartilage, *ALDH1A2* is overexpressed with FDR = 0.0017 and logFC = 0.38 and *CRADD* is underexpressed with FDR = 0.00067 and logFC = −0.24), thus providing additional supportive evidence for a role in osteoarthritis.

Next, we tested whether high-confidence effector genes correlate with nearby methylation sites, which in turn putatively mediate the effect of osteoarthritis-risk variants. Using expression and methylation data of the same osteoarthritis-affected individuals in the same tissue, we identified such expression quantitative trait methylation (eQTM) effects at nominal significance (p < 0.05) for three genes (*ALDH1A2*, *FAM53A*, and *RPP25*) in low-grade cartilage and one gene (*LTBP1*) in high-grade cartilage (Figure 7C). To assess whether these observed associations are solely correlations, or whether methylation levels do have a causal effect on gene expression (by mediating the genetic effect on gene expression), we performed one-sample MR (supplemental subjects and methods). We found evidence (MR p < 0.05) for a causal effect of methylation on gene expression levels for two genes (*ALDH1A2* and *RPP25*) in low-grade osteoarthritis cartilage and one gene (*LTBP1*) in high-grade osteoarthritis cartilage (Table S13). These findings suggest that methylation mediates the effect of genetic variants on expression for these high-confidence effector genes.

### Comparing colocalization of osteoarthritis loci in joint and whole blood mQTL data

To investigate the joint tissue specificity of colocalizing joint mQTL and osteoarthritis GWAS data, we asked whether these results could also be identified in whole blood (supplemental note S4). This would allow us to better understand whether the regulatory effects of osteoarthritis-risk loci mediated by proximal methylation sites are exclusive to disease-affected joint tissues or also observed in peripheral tissues. We tested whether the pairs of risk variant-methylation sites that colocalize in at least one joint tissue also colocalize in a large whole blood mQTL meta-analysis.^40^

Considering all OA-risk variants, we found 15 risk variant-methylation site pairs for which we estimated colocalizing GWAS signals and mQTL in at least one joint tissue (ten and five pairs in low-grade and high-grade osteoarthritis cartilage, respectively), but not in whole blood. These pairs involved eight all OA-risk variants in total (seven and three all OA signals in low-grade and high-grade osteoarthritis cartilage, respectively).

For the knee OA-risk variants, we identified five risk variant-methylation site pairs with colocalizing GWAS signals and mQTL in at least one joint tissue (two, two, and one pairs in low-grade and high-grade osteoarthritis cartilage and synovium, respectively) but not in whole blood. These pairs involved two unique knee OA-risk variants (rs9277552 in low-grade and high-grade osteoarthritis cartilage and rs56116847 in synovium). For the TKR-risk variants, we did not find evidence for joint-tissue-specific colocalizations.

## Discussion

Osteoarthritis is a common disease with a complex polygenic architecture. In this study, we analyzed the genome-wide methylation profile of low-grade osteoarthritis cartilage, high-grade osteoarthritis cartilage, and synovium at unprecedented scale and depth. We identified and biologically characterized DNA methylation markers of osteoarthritis grade and generated genome-wide maps of mQTLs in three understudied osteoarthritis-relevant tissues, which we used to identify mechanistically relevant genes.

Our data revealed global differences in the methylation profile between tissue types (cartilage versus synovium) and cartilage degeneration states (low-grade versus high-grade osteoarthritis cartilage), with robust evidence for replication in an independent dataset despite lower power due to smaller replication sample size. This study represents a large EWAS for knee cartilage degeneration, increasing the number of studied knee osteoarthritis-affected individuals by almost 6-fold, thus providing substantially higher power compared to previous studies. Together, our findings underline the cell type and osteoarthritis-grade specificity of DNA methylation in primary tissues, thus highlighting the importance of expanding molecular studies of complex diseases to multiple relevant tissues and cell types.

Indeed, comparison of our findings with methylation data available in peripheral blood further underlined the value of analyzing primary tissues. Observed differences included mQTLs with opposite directions of effect and evidence for colocalization in joint tissue, but not in whole blood, for genetic variants linked with osteoarthritis. These findings suggest that at least a subset of the regulatory effects conferred by osteoarthritis-linked variants through proximal methylation sites are specific to osteoarthritis-affected tissue. More generally, they emphasize the value of investigating disease-relevant tissues rather than solely relying on molecular data in peripheral tissue types.

Characterization of knee cartilage degeneration methylation markers revealed the involvement of biological processes such as external matrix organization, skeletal system development, and signaling pathways, which mirror the broad spectrum of physiological mechanisms observed during cartilage degeneration.^52^ Our results indicate that the aetiology of osteoarthritis is partly regulated through aberrant DNA methylation. Notably, we report an enrichment of the epithelium-related term “positive regulation of epithelial cell migration.” Given the role of epithelial cells in lining body cavities, in particular blood vessels, this finding may suggest that methylation is involved in the pathogenic release of pro-angiogenic factors. Our findings provide evidence that epithelium-linked mechanisms are relevant in osteoarthritic changes of the articular cartilage in affected joints.

Our study presents a genome-wide map of mQTLs in low-grade and high-grade osteoarthritis cartilage as well as in the synovium of osteoarthritis-affected knees. We identified 18 differential mQTLs between low-grade and high-grade osteoarthritis cartilage. This finding suggests distinct regulatory effects of genetic variants on methylation early and late in the cartilage degeneration process, thus proposing changing genetic influences on epigenetic profiles during osteoarthritis progression.

We identified methylation sites that play a putative causal role in osteoarthritis, for example for the *WWP2*, *BSN*, and *MFHAS1* genes. *WWP2* codes for WW domain-containing E3 ubiquitin protein ligase 2, which is involved in protein ubiquitination. *WWP2* is the host gene of micro RNA 140, a key regulator in chondrocytes, which is targeted by methylation in that region. *Wwp2* has previously been implicated in cartilage homeostasis through regulation of *Adamts5*, a gene encoding an aggrecanase. In addition, *WWP2* demonstrates decreased expression levels in osteoarthritis-affected articular cartilage derived from samples of affected individuals.^53^ Our findings indicate that methylation may be driving this aberrant mechanism. Previously, a study identified an mQTL that targets methylation sites in *WWP2*.^17^ Another study found *WWP2* expression to be significantly associated with proximal genetic variants and methylation levels of close methylation sites.^11^ Together, these results support a role for genetically determined methylation for *WWP2* regulation in osteoarthritis. *BSN* encodes a protein involved in neurotransmission. In the active zone of the synapse, BSN is part of the scaffold of the presynaptic skeleton complex, a structure that assists in the vesicle fusion of synaptic vesicles and presynaptic membranes.^54^ This finding may point to innervation in cartilage and synovium during osteoarthritis. *MFHAS1* plays a role in controlling Toll-like receptors TLR2 and TLR4,^48^^,^^49^ which in turn promote inflammation of the synovium. Toll-like receptors are exposed by cells in the synovium. They bind released matrix molecules of degraded cartilage, which leads to the formation of chemokines and cytokines, in turn leading to the inflammatory cell infiltration of the synovium.^55^

We found evidence for 56 methylation sites mediating the effects of proximal osteoarthritis-linked genetic variants in osteoarthritis-relevant tissue. For seven genes (*ALDH1A2*, *CHMP1A*, *CRADD, FAM53A*, *LTBP1*, *RPP25*, and *TGFA*), we found evidence that GWAS signals for osteoarthritis colocalize with mQTLs in these genes and are additionally associated with gene expression levels in the same tissue. Four of these genes (*ALDH1A2*, *FAM53A*, *LTBP1*, and *RPP25*) showed an association between expression and methylation. Together, our results provide support for a regulatory role of the associated genetic variants across two molecular layers, and altered gene expression is modulated through genetically determined DNA methylation levels.

CRADD is an adapter protein involved in apoptosis and plays a role in the formation of the PIDDosome-complex, which in turn triggers *CASP2*.^56^ A role for dysregulated apoptosis in osteoarthritis synovial tissue has been previously suggested.^57^ Our findings indicate that the apoptosis-contributing factor *CRADD* is regulated through DNA methylation in synovium. *ALDH1A2* codes for an enzyme that catalyzes the reaction from retinaldehyde to retinoic acid, an activated form of vitamin A. Retinoic acid has been linked to the degeneration of collagen in bone^58^ and is further used as an agent to induce matrix degeneration in cartilage samples.^59^ LTBP1 plays an essential role in the regulation of transforming growth factor (TGF) betas, a cytokine class that has been involved in extracellular matrix synthesis and maintenance, but also moderates the effects of inflammation and controls hypertrophy of chondrocytes.^60^ TGF betas are produced by chondrocytes in their inactivated form. LTBP1 binds these inactive TGF-betas to the extracellular matrix in cartilage.^61^

Together, causal inference and colocalization analyses point to methylation sites that putatively contribute to osteoarthritis in synovium as well as in early (low-grade osteoarthritis cartilage) and late disease stages (high-grade cartilage).

In summary, our results highlight the cell type as well as disease-grade specificity of the methylome in osteoarthritis-relevant tissue. We identify evidence for the involvement of epithelium-related pathways and identify likely effector genes for hitherto unresolved osteoarthritis GWAS signals. In several cases, we are able to decipher the molecular mechanism underpinning these associations and demonstrate an important role for DNA methylation in the aetiopathogenesis of this debilitating disease.

## Data Availability

Methylation QTL, Mendelian randomization, and differential methylation results can be obtained online (hmgubox and the Downloads page of the Musculoskeletal Knowledge Portal, see web resources). All software used in this study is available from free repositories or manufacturers as referenced in the web Resources and supplemental subjects and methods.

## Web resources

An epigenome-wide view of osteoarthritis in primary tissues, https://hmgubox.helmholtz-muenchen.de/d/a23fce319fd844d4b293/
coloc.fast function, https://github.com/tobyjohnson/gtx/blob/526120435bb3e29c39fc71604eee03a371ec3753/R/coloc.R
Ensembl Variant Effect Predictor, http://grch37.ensembl.org/Homo_sapiens/Tools/VEP/
Gene Expression Omnibus database, https://www.ncbi.nlm.nih.gov/geo/ (GEO: GSE63106)
Genetics of DNA Methylation Consortium, http://mqtldb.godmc.org.uk/
GWAS catalog, https://www.ebi.ac.uk/gwas
MetaTissue, http://genetics.cs.ucla.edu/metatissue/
Musculoskeletal Knowledge Portal, http://mskkp.org

## Supplemental information

Document S1. Figure S1–10, supplemental notes S1–S4, and supplemental subjects and methods

Data S1. Table S1–S13

Document S3. Article plus supplemental information
