# Supplementary material for: An epigenome-wide view of osteoarthritis in primary tissues
Source: Am J Hum Genet. 2022 Jun 8;109(7):1255–71. doi: 10.1016/j.ajhg.2022.05.010 (PMC9300761; doi:10.1016/j.ajhg.2022.05.010)
Supplement: Document S1. Figure S1–10, supplemental notes S1–S4, and supplemental subjects and methods [file mmc1.pdf]

The American Journal of Human Genetics, Volume *109*

Supplemental information

An epigenome-wide view of osteoarthritis in primary tissues

Peter Kreitmaier, Matthew Suderman, Lorraine Southam, Rodrigo Coutinho de Almeida, Konstantinos Hatzikotoulas, Ingrid Meulenbelt, Julia Steinberg, Caroline L. Relton, J. Mark Wilkinson, and Eleftheria Zeggini

Supplemental Material

Supplemental Figures:

Figure S1

EWAS reveal hyper-and hypomethylated regions Volcano plot visualises the beta coefficients of methylated regions. Black dots indicate significant DMRs (Bonferroni adjusted p values < 0.05) between low-grade and high-grade osteoarthritis cartilage. We identified 1,701 hypermethylated and 776 hypomethylated DMRs.

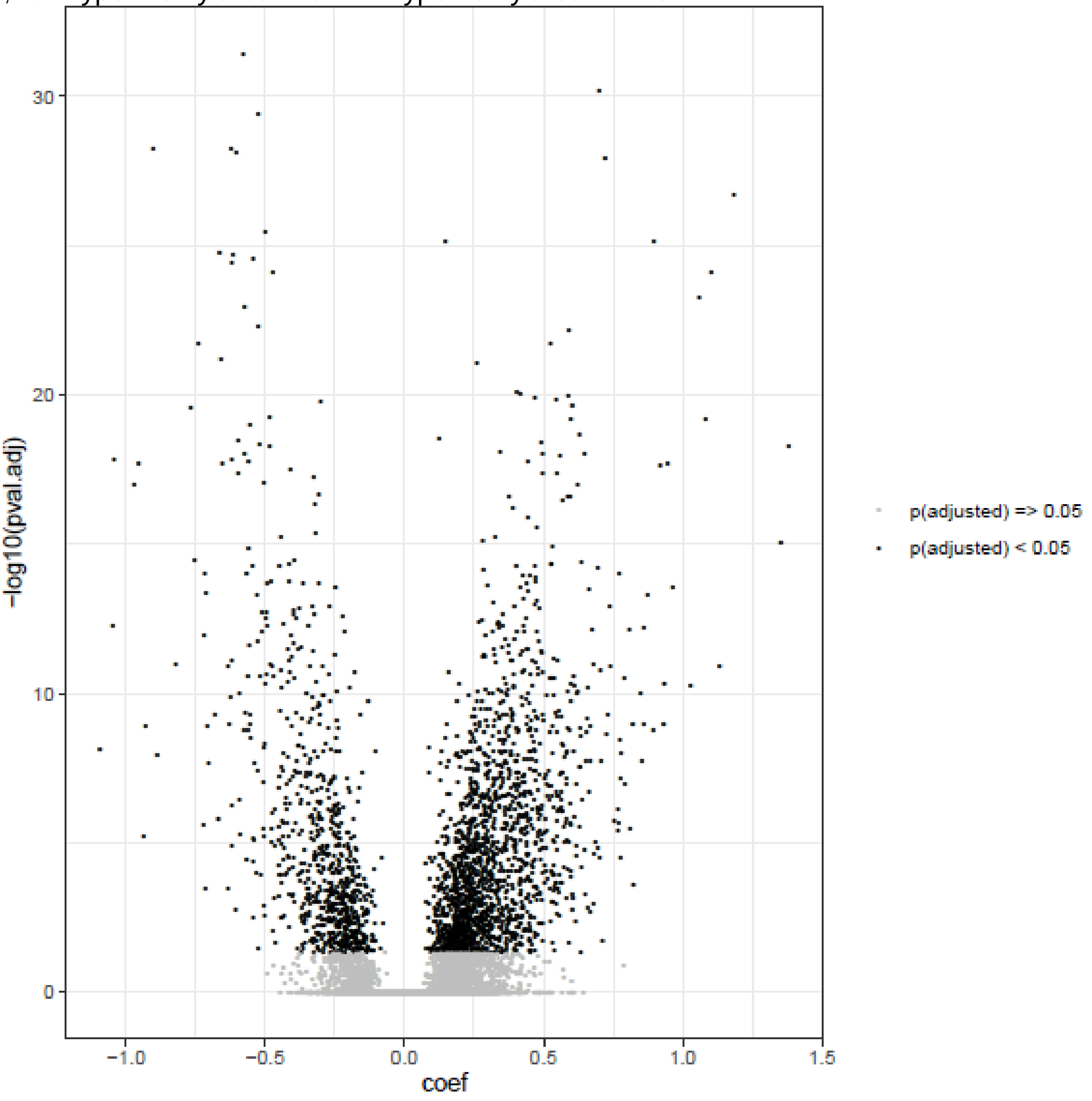

Figure S2

Pathway analysis reveals enrichment of four KEGG terms. Most significant KEGG terms enriched in 15,328 DMSs. Red dashed lines indicate the significance threshold (Benjamini-Hochberg adjusted $p < 0.05$).

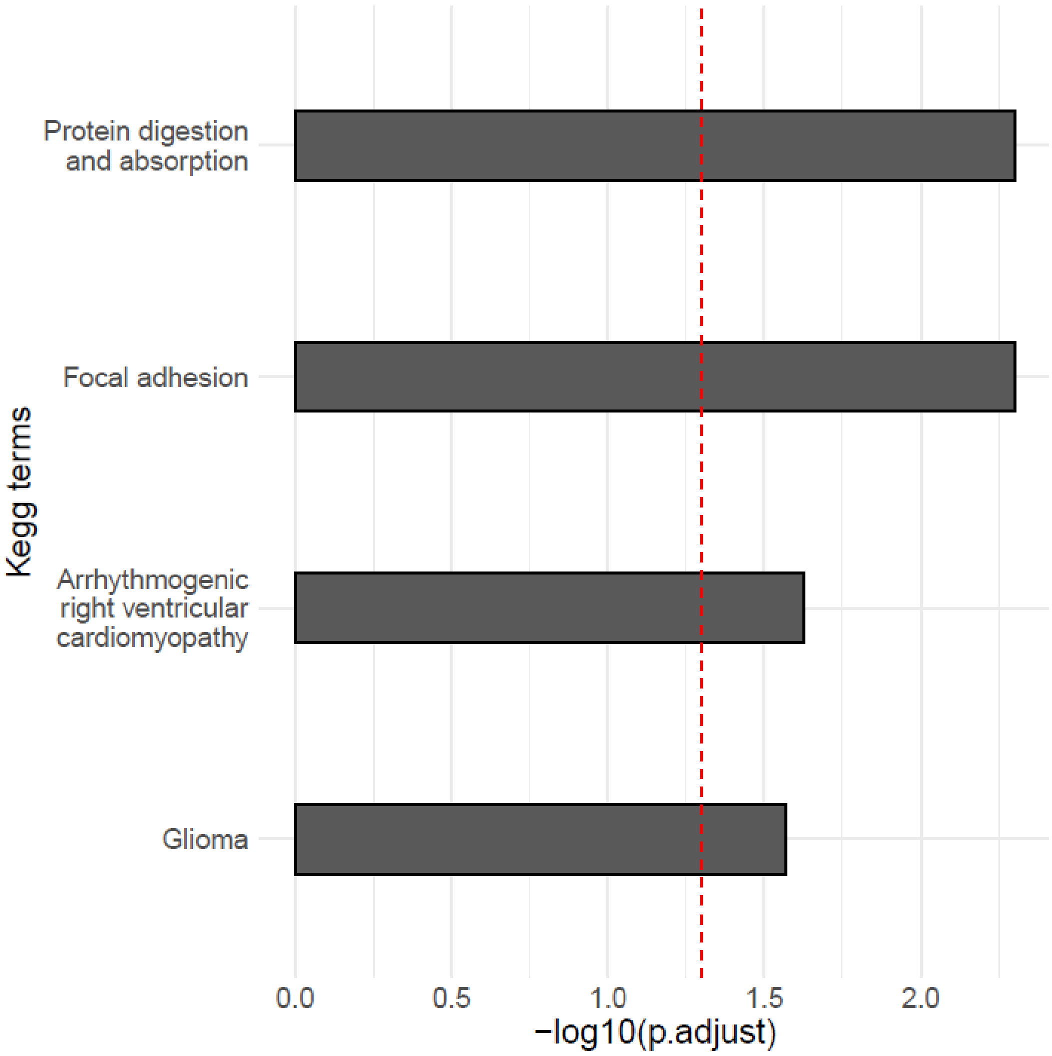

Figure S3

ML-based classifier distinguishes low-grade and high-grade osteoarthritis cartilage

Using cross-validation, we trained and tested Random-Forest based classifiers that distinguish low-grade from high-grade osteoarthritis cartilage samples, the resulting ROC curve is depicted. The area under the curve is 0.967

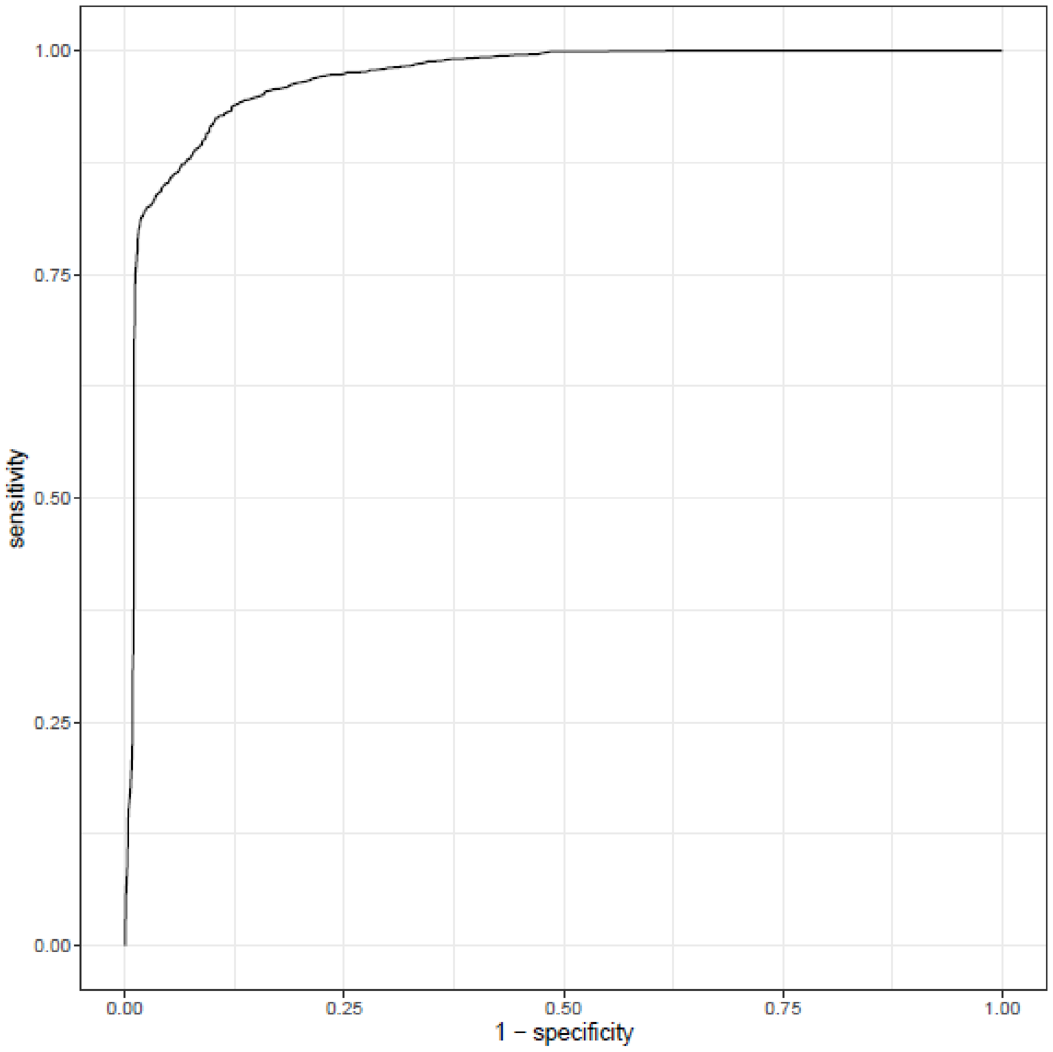

Figure S4

The mQTL landscape in cartilage and synovium Manhattan plots depicting the lowest association p value per SNP with close methylation sites (< 1 Mb) in low-grade osteoarthritis cartilage (A), high-grade osteoarthritis cartilage (B) and synovium (C) on genome-wide scale. The red lines indicate genome-wide significance (Bonferroni p < 0.05).

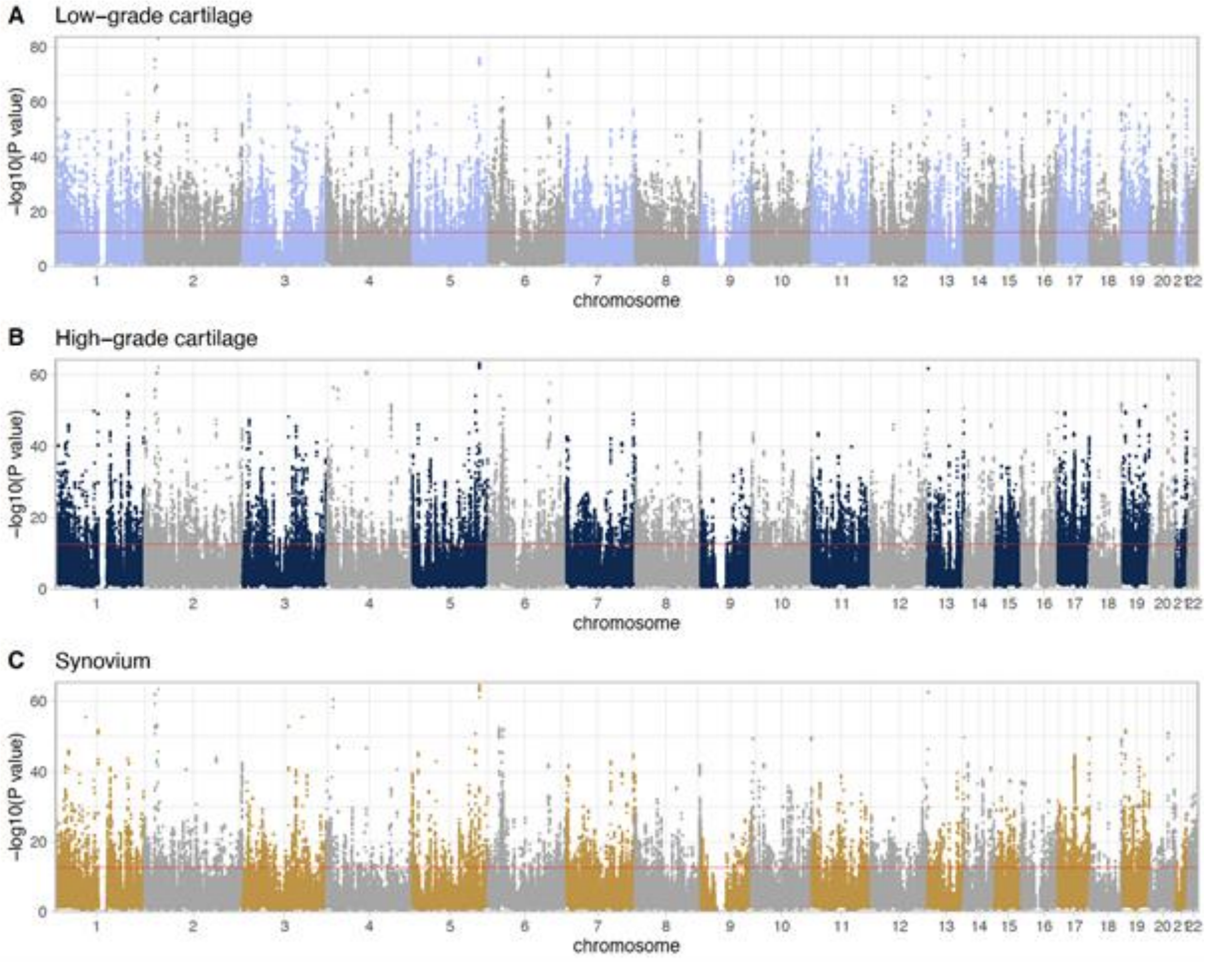

Figure S5

Insights into the mQTL architecture in low-grade osteoarthritis cartilage. Barplots showing the proportions (A) and enrichments (C) of gene annotations of methylation sites with at least one mQTL in low-grade osteoarthritis cartilage. Analogously, barplots B and D describe proportions and enrichments of CpG island annotations, respectively. In plot C and D, all enrichments pass the respective Bonferroni threshold (C: $p < 0.0071$, D: $p < 0.0083$). Plot E describes the number of targeted methylation sites across mQTL, showing that most mQTL are associated with few methylation sites. Similarly, plot F describes the number of mQTL across methylation sites, showing that most methylation sites have a few mQTLs.
The dashed blue line in plots C and D refers to the fold enrichment of 1. Msite = methylation site; TSS: Transcription start site; UTR: Untranslated region; Island: Methylation site within a CpG island; N_Shore and S_Shore: Methylation site within 2kb upstream and downstream around the starting site of a CpG island, respectively; N_Shelf and S_Shelf: Methylation site between 2kb and 4kb upstream and downstream of a CpG island, respectively; OpenSea: Methylation sites further then 4 kb away from a CpG Island.

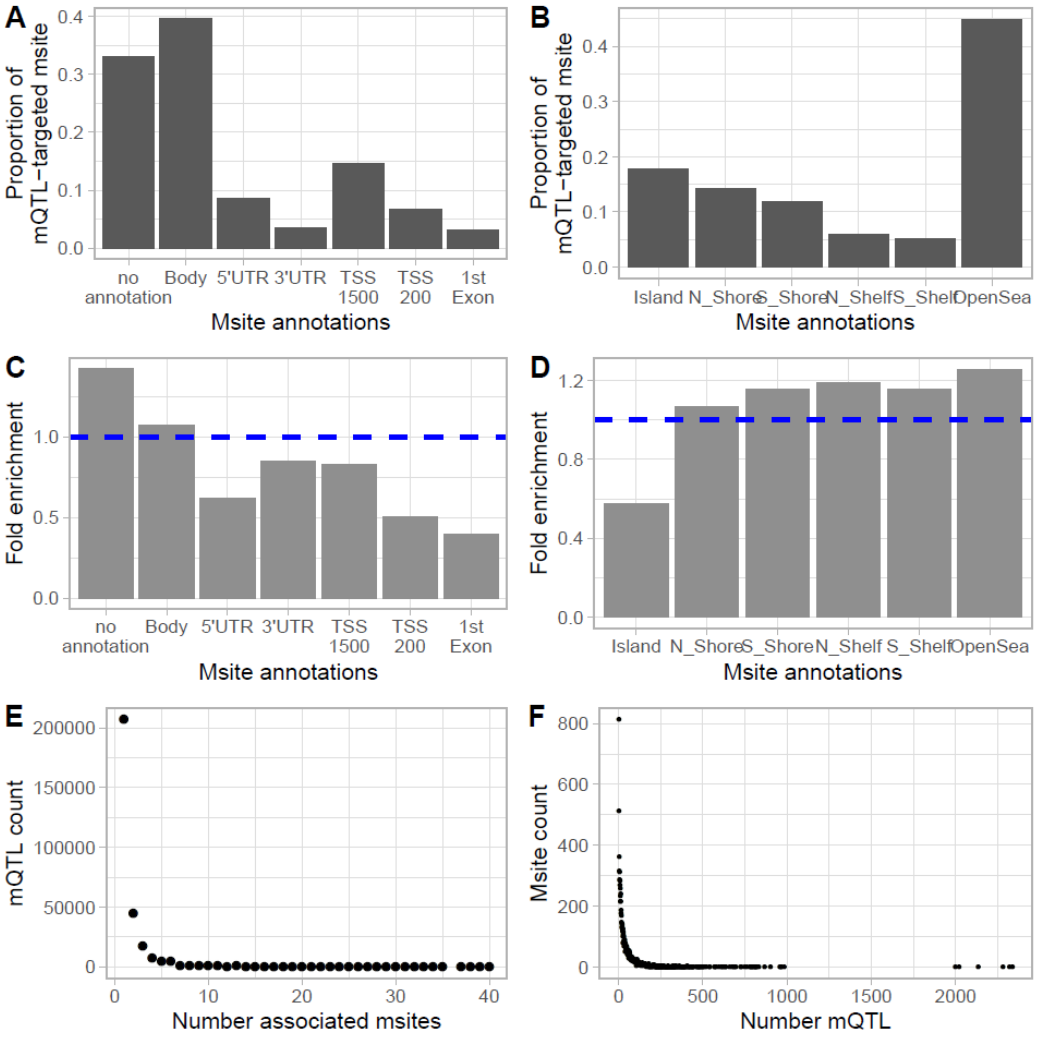

Figure S6

Insights into the mQTL architecture in high-grade osteoarthritis cartilage

Analog to Figure S5, but for the mQTL profile in high-grade osteoarthritis cartilage. In plot C and D, all enrichments pass the respective Bonferroni threshold (C: $p < 0.0071$, D: $p < 0.0083$). Msite = methylation site

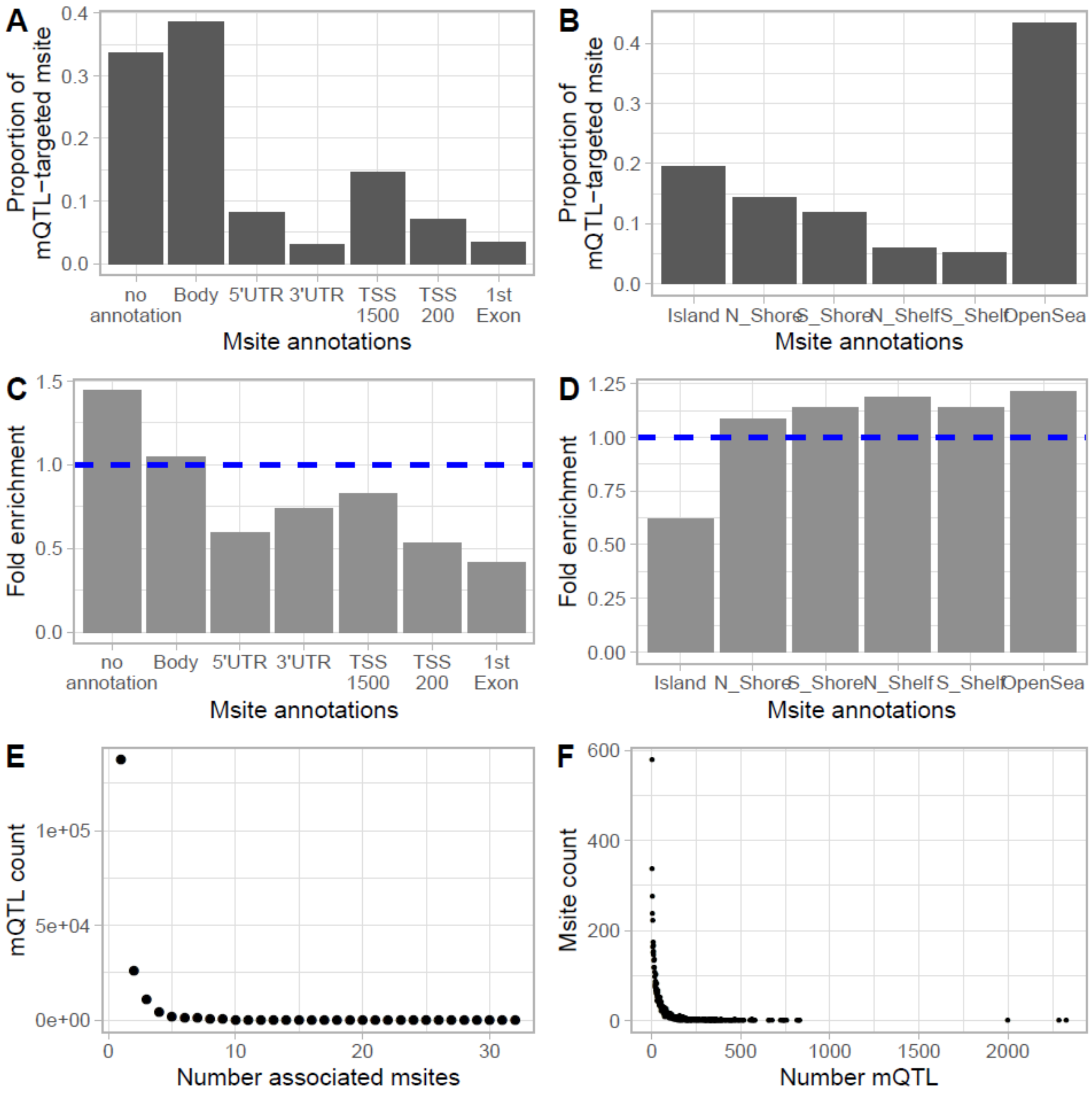

Figure S7

Insights into the mQTL architecture in synovium

Analyses analog to Figure S5, but for methylation sites targeted in synovium. In plots C and D, enrichments passing the respective Bonferroni threshold (C: $p < 0.0071$, D: $p < 0.0083$) are dark grey, otherwise light grey.

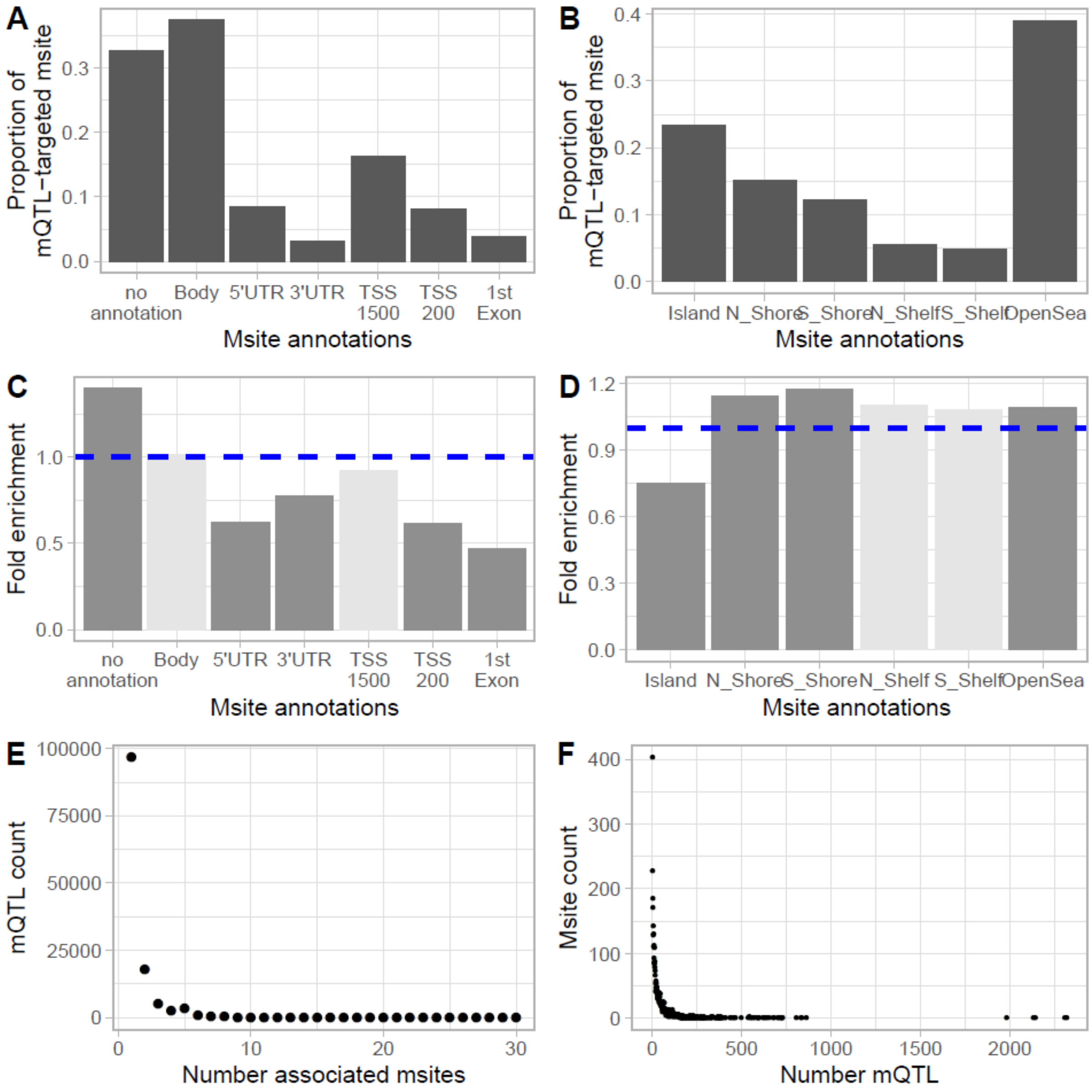

Figure S8

Most significant, sex-specific mQTL effects in thre osteoarthritis tissues. The boxplots describe the most signficiant, sex-specific mQTL in (A) low-grade (beta = 2.32, SE = 0.19, FDR = 6.02 x10^{-12}) and (B) high-grade osteoarthritis cartilage (beta = 3.10, SE = 0.25, FDR = 2.95x10^{-11}) as well as in (C) synovium (beta = 2.90, SE = 0.14, FDR = 1.21x10^{-23}) (C). The boxplots represent 25th, 50th and 75th percentiles, and whiskers extend to 1.5 times the interquartile range.

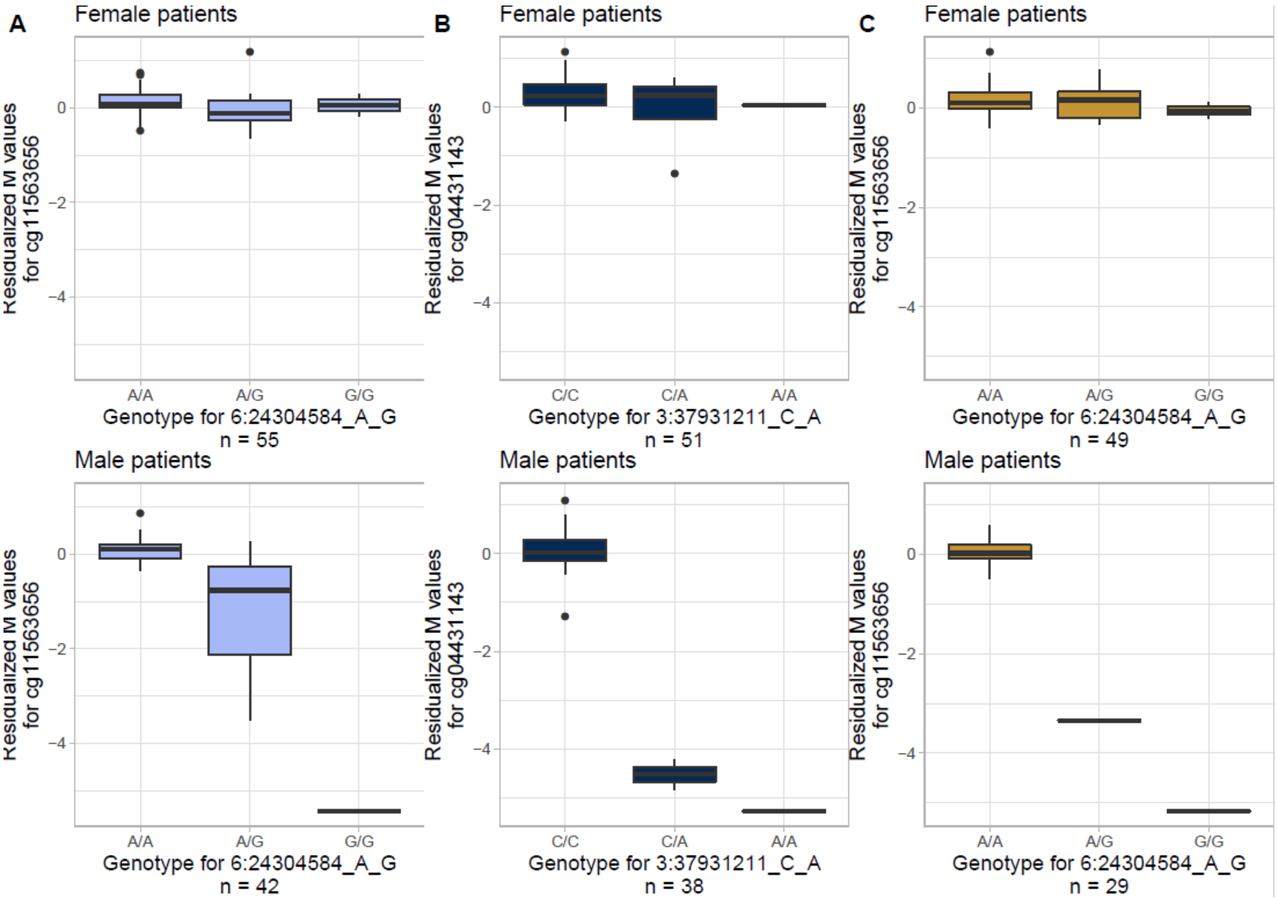

Figure S9

An overview of the Mendelian randomization approach. We estimated the putative causal effect of a methylation site (exposure) on osteoarthritis (outcome). We preselected 15,328 methylation sites which we previously linked to cartilage degeneration in the EWAS. Subsequently, we performed the 2SMR approach per tissue (low-grade osteoarthritis cartilage, high-grade osteoarthritis cartilage and synovium). Of the 15,328 methylation sites, we retained the ones with at least one significant mQTL (FDR < 0.05) and used these mQTLs as instrumental variables (IV) to estimate the putative causal effect of the methylation sites (exposure) on three osteoarthritis-relevant traits. For methylation sites with exactly one independent IV, we performed the Wald-ratio test for methylation sites, otherwise the inverse-variance-weighted (IVW) method

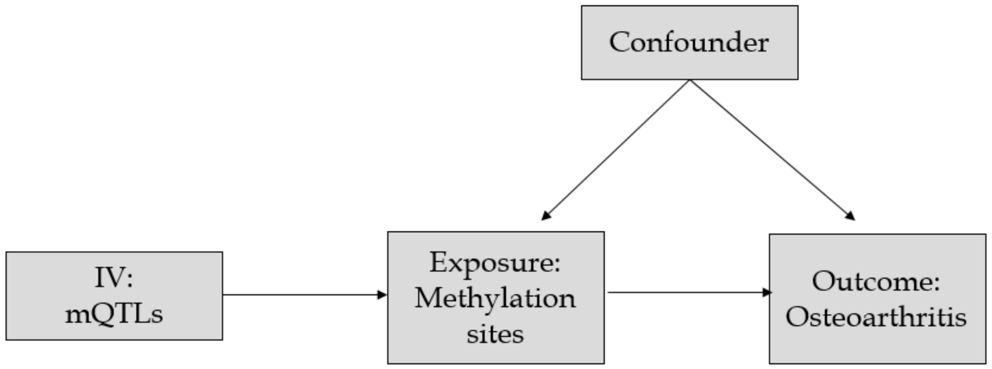

Figure S10
An overview of the Mendelian randomization approach in the reverse direction. We sought to estimate the putative causal effect of osteoarthritis (exposure) on methylation (outcome). We tested every osteoarthritis trait-methylation site trait combination (low-grade osteoarthritis cartilage: n = 10,099, high-grade osteoarthritis cartilage: n = 6,110, synovium: n = 4,662) that we examined in the opposite direction (Figure S9). We used GWAS risk SNPs as instrumental variables (all OA: n = 27, knee OA: n = 10, TKR: n = 4). We applied the IVW method.

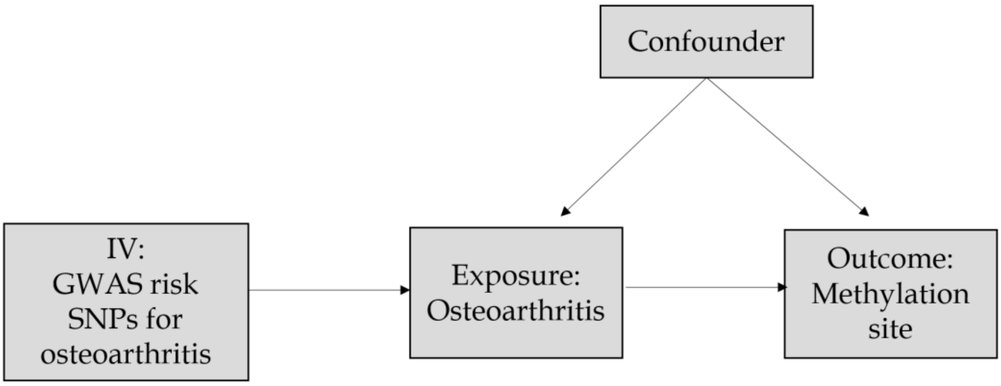

Supplemental Tables:

Table S1. Overview of patient characteristics per tissue, related to the Subjects and Methods
Table S2. List of 15,328 differentially methylated sites, related to Epigenome-wide association study reveals widespread, robustly-replicating signals
Table S3. List of 2,477 differentially methylated regions, related to Epigenome-wide association study reveals widespread, robustly-replicating signals
Table S4. Biological terms that are enriched among 15,328 differentially methylated sites, related to Epigenome-wide association study reveals widespread, robustly-replicating signals
Table S5. Biological terms that are enriched among 7,192 replicated differentially methylated sites, related to Epigenome-wide association study reveals widespread, robustly-replicating signals
Table S6. Numbers of significant mQTL effects per tissue, related to Genome-wide mQTL maps in osteoarthritis-relevant tissues
Table S7. Overview of sex-specific mQTL (FDR < 0.05) in low-grade OA cartilage, related to Genome-wide mQTL maps in osteoarthritis-relevant tissues
Table S8. Overview of sex-specific mQTL (FDR < 0.05) in high-grade OA cartilage, related to Genome-wide mQTL maps in osteoarthritis-relevant tissues
Table S9. Overview of sex-specific mQTL (FDR < 0.05) in synovium, related to Genome-wide mQTL maps in osteoarthritis-relevant tissues
Table S10. List of 195 differential mQTLs, related to figure 5 and Identification of grade-specific mQTLs in cartilage
Table S11. List of significant Mendelian randomization results, related to figure 6 and Assessing the causal role of methylation in osteoarthritis

Table S12. List of colocalising GWAS and mQTL signals, related to figure 7 and Resolution of GWAS signals
Table S13. List of putative causal effects of methylation on gene expression in joint tissues, related to Resolution of GWAS signals

<u>Supplemental Notes</u>

Supplemental Note 1: Power analysis for differential analysis in osteoarthritis cartilage

Based on RNA-seq data of matching low-grade and high-grade OA cartilage samples, the power of this study (n = 90 patients) to detect differentially expressed genes is 70 % at FDR 5% (Steinberg and Zeggini, 2016). This is a substantial improvement compared to the next largest study (which was 17 knee OA samples) with the predicted power of ~20% at the same significance level.

Supplemental Note 2: Epigenome-wide association study reveals widespread, robustly-replicating signals

To identify DNA methylation markers of cartilage degeneration, we performed an epigenome-wide association study (EWAS) on paired low-grade and high-grade osteoarthritis cartilage samples from 90 patients across 401,870 methylation sites. We identified 15,328 differentially methylated sites (DMS) distributed across the whole genome (significance threshold of $p < 1.24x10^{-7}$; Methods), which constitutes 3.81% of all tested sites (Figure 2 A upper panel, Table S1). Of these DMSs, 8,783 (57%) and 6,545 (43%) showed hypermethylation and hypomethylation in high-grade osteoarthritis cartilage, respectively (Figure 2 B and C). We found a significant overrepresentation of hypermethylated sites among DMSs (binomial test $p = 1.57x10^{-73}$).

In epigenome-wide association studies, it is common to estimate differentially methylated regions (DMRs). As methylation levels of proximal cytosines tend to correlate with each other (Eckhardt et al., 2006), this approach enables dimensionality and redundancy reduction and focuses on robust signals. We identified 2,477 DMRs (Bonferroni adjusted $p < 0.05$), each consisting of at least 2 methylation sites (Figure 2 A bottom panel, Table S2). Of these, 1,701 (69%) and 776 (31%) DMRs were hyper- and hypomethylated in high-grade osteoarthritis cartilage, respectively (Figure S1). The mean length of DMRs was 211.74 (sd: 198.36). The shortest and longest DMR is three and 1,608 bp long, respectively.

We conducted pathway enrichment analyses for the 15,328 DMS. We identified significant over-representations (Benjamini-Hochberg adjusted $p < 0.05$) for 29 Gene ontology (GO) and 4 KEGG terms (Table S3). The five most significant GO and four KEGG terms are shown in Figure 2 D and Figure S2. The GO enrichment analysis revealed biological processes that have been previously linked to osteoarthritis, including signals linked to external matrix organization (Rushton et al., 2014), skeletal system development (Bonin et al., 2016; Rushton et al., 2014; Steinberg et al., 2017; Zhang et al., 2016), cell adhesion (Alvarez-Garcia et al., 2016; Steinberg et al., 2017), signaling pathways (e.g. integrin signaling pathway (Steinberg et al., 2017) and platelet activation (Steinberg et al., 2017). In addition, we identified for the first time enrichment of the epithelium-related term "positive regulation of epithelial cell migration" in articular cartilage which may point to a novel etiological mechanism. We did not identify enrichments among 2,477 DMRs.

To replicate our findings, we performed an EWAS between matched low-grade and high-grade osteoarthritis cartilage samples from an independent dataset comprising 17 knee osteoarthritis patients (den Hollander et al., 2014, 2015). As in the discovery analyses, we analysed data on the methylation site and on a regional level. On the site level, 13,420 DMSs

(87.6%) from the discovery data were present in the replication set. Of those, 13,001 (96.9%) showed the same direction of effect in the replication set, 7,192 (53.6%) at nominal significance ($p < 0.05$). The effect sizes of the 7,192 DMSs in the discovery and replication sets were highly correlated (Pearson $r = 0.96$, $p < 2.2 \times 10^{-16}$) (Figure 3A). Of these replicated DMSs, 3,231 (44.92%) and 3,961 (55.07%) were hypermethylated and hypomethylated in high-grade osteoarthritis cartilage, respectively. A binomial test showed a significant overrepresentation of hypomethylated sites among replicated DMSs ($p = 3.87 \times 10^{-18}$). At the region level, we conservatively considered methylated regions that are composed of exactly the same methylation sites in the discovery and replication set. Given this definition, 105 of 2,477 DMRs (4.2%) were present in the replication set. For all of these, the effects in the replication set were in the same direction as the discovery set and all were nominally significant ($p < 0.05$). Furthermore, the effect sizes of these 105 replicating DMRs set were highly correlated between the discovery and replication datasets (Pearson r: 0.95, p-value < 2.2×10^{-16}) (Figure 3B). These results point to the robustness of the identified methylation changes. Performing pathway enrichment analysis on 7,192 replicated DMSs identified 18 GO terms (Table S5) and highlighted pathways related to skeletal system development, cell adhesion and lipid metabolic processes.

To assess sex-specific markers of cartilage degeneration, we performed EWAS separately on paired low-grade and high-grade cartilage in female ($n = 52$) and male ($n = 38$) patients.
In female patients, we identified 49,695 differentially (FDR < 0.05) methylated sites (DMS). A number of 23,494 and 26,201 showed hypo- and hypermethylation in high-grade cartilage samples, respectively. In male patients, we identified 44,059 DMS (21,355 and 22,704 are hypo- and hypermethylated in high-grade cartilage, respectively).
Of note, we identified methylation sites that show sex-specific effects. In female patients, 1,338 methylation sites showed effects with FDR < 0.05, but $p > 0.05$ in men and in the combined analysis. Analogously, we identified 3,316 methylation sites with male-specific effects. These results suggest sex-specific effects in osteoarthritis cartilage.

Supplemental Note 3: Overlap between mQTL maps and loci of previously reported mQTLs

We tested whether the mQTL maps of this study revealed genetic-epigenetic effects in regions of previously reported mQTLs in osteoarthritis-relevant tissue (Aubourg et al., 2021; Boer et al., 2021). More specifically, we sought to overlap the mQTL maps with 25 and 5 regions of previously identified mQTL effects (+/- 500 kb) in cartilage and synovium, respectively.
In low-grade osteoarthritis cartilage, we identified at least one significant mQTL effect in our mQTL map in 24 of 25 tested regions. These regions contained 28 previously reported mQTLs (Bonferroni $p < 0.05$)
In high-grade osteoarthritis cartilage, we found at least one significant mQTL effect in our mQTL map in 20 of 25 tested regions. These regions contained 28 previously reported mQTLs (Bonferroni $p < 0.05$)
In synovium we determined at least one significant mQTL effect in our mQTL map in all 5 tested regions. These regions contained 4 previously reported mQTLs (Bonferroni $p < 0.05$)

Supplemental Note 4: Sensitivity analysis of colocalisation: comparison between osteoarthritis tissues and peripheral blood

Performing the colocalisation between osteoarthritis GWAS and whole blood mQTL data (section “Comparing colocalization of osteoarthritis loci in joint and whole blood mQTL data”) involved fewer variants due to the smaller set of overlapping variants. This could limit the ability to identify colocalising signals, making it unclear whether we can attribute the missing signal to the tissue specificity of the mQTL data. To account for this, we again performed colocalisation between joint mQTL and GWAS data, this time on reduced variant sets (same

variant number as between whole blood mQTL and GWAS data). We then filtered for risk variant-methylation site pairs that showed evidence for colocalisation between osteoarthritis GWAS and (1) joint mQTL data when considering a reduced variant set, but (2) not in whole blood.

Using this strict filtering, we found three all OA variant-methylation site pairs (involving the all OA risk variants rs798726, rs12154055 and rs2856821) and one knee OA variant-methylation site pair (involving the knee OA risk variant rs9277552). Furthermore, we found one knee OA variant-methylation site pair (involving the knee OA risk variant rs56116847) that colocalises in synovium, but not in whole blood.

For example, risk variant rs798726 colocalised with methylation site cg07929082 in low-grade osteoarthritis cartilage with a high probability (93.9% and 97.2% considering the largest possible and a reduced variant set, respectively). However, in whole blood, the colocalisation probability was very low (0.000008%).

Supplemental Subjects and Methods

RESOURCES TABLE

REAGENT or RESOURCE	SOURCE	IDENTIFIER
**Deposited data**		
Summary statistics of differential methylation analysis, Mendelian randomization and methylation QTL data		https://hmgubox.helmholtz-muenchen.de/d/a23fce319fd844d4b293/
Full summary statistics of the methylation QTL analysis		http://mskkp.org
Replication data set	(den Hollander et al., 2014)	https://www.ncbi.nlm.nih.gov/geo/ id: GSE63106
**Software and algorithms**		
R		version 3.5.3
meffil	(Min et al., 2018)	R package, version 1.0.0
minfi	(Aryee et al., 2014; Fortin et al., 2017)	R package, version 1.28.4
IlluminaHumanMethylation450kanno.ilmn12.hg19		R package, version 0.6.0
limma	(Ritchie et al., 2015)	R package, version 3.38.3
sva	(Leek et al., 2012)	R package, version 3.30.1
dmrff	(Suderman et al., 2018)	R package, version 0.0.2
missMethyl	(Maksimovic et al., 2021)	R package, version 1.24.0
MatrixEQTL	(Shabalin, 2012)	R package, version 2.2
vcfR	(Knaus and Grünwald, 2017)	R package, version 1.8.0.
MetaTissue	(Sul et al., 2013)	http://genetics.cs.ucla.edu/metatissue/ Software, version 0.5
GWAS catalog		https://www.ebi.ac.uk/gwas/
GoDMC	(Min et al., 2021)	http://mqtldb.godmc.org.uk/
biomaRt	(Durinck et al., 2009)	R package, version 2.38.0
METAL	(Willer et al., 2010)	
TwoSampleMR	(Hemani et al., 2018)	R package, version 0.4.25

PLINK		Software, version 1.9
coloc.fast		https://github.com/tobyjohnson/gtx/blob/526120435bb3e29c39fc71604eee03a371ec3753/R/coloc.R
ivreg		R package version 0.6-1
FactoMineR	(Lê et al., 2008)	R package, version 2.0
caret		R package, version 6.0.84

Patients and study samples

Samples from osteoarthritis affected knees were collected in 101 patients that underwent total knee replacement due to late-stage osteoarthritis. The patients were collected in 3 cohorts. Cohort1 comprised 13 knee osteoarthritis patients (10 male and 3 female patients) with a mean age of 68, Cohort2 included 18 knee osteoarthritis patients (5 male and 13 female patients) with a mean age of 70 and Cohort 3 consisted of 70 knee osteoarthritis patients (28 male and 42 female patients) with a mean age of 70. Low-grade and high-grade osteoarthritis cartilage samples were collected from each patient, samples from the synovium of patients from Cohort2 and Cohort3. Cartilage samples were graded using the OARSI cartilage classification system (Cohort1) or International Cartilage Repair Society (ICRS) scoring system (Cohort2 and Cohort3; low-grade and high-grade osteoarthritis cartilage is signified by ICRS grades 0 or 1 and 3 or 4, respectively). This work was approved by Oxford NHS REC C (10/H0606/20 and 15/SC/0132), and samples were collected under Human Tissue Authority license 12182, Sheffield Musculoskeletal Biobank, University of Sheffield, UK. Before participating in the study, all patients provided written, informed consent.

Sample extraction

A previous study (Steinberg et al., 2021) reported the isolation of the chondrocytes (section “Isolation of chondrocytes”), the isolation of synoviocytes (section “Isolation of synoviocytes”) and DNA extraction (section “DNA, RNA and protein extraction”) in its methods part.

DNA methylation data

Genome-wide DNA methylation was measured using the Illumina 450k array in three batches. Batch 1 and 2 were generated using the Infinium HumanMethylation450 Bead Chip (450k array) batch 3 using the Illumina Infinium MethylationEPIC Bead Chip (EPIC array). We used the R package minfi to read idat files (Aryee et al., 2014; Fortin et al., 2017) and combined samples measured on 450k and EPIC arrays. We limited the methylation data to 452,832 methylation sites present on both array types. Based on genotype data, we identified three ethnicity outliers and removed methylation samples from these patients. We performed quality control per sample using the qc.summary function in the R package meffil (Min et al., 2018) and removed gender mismatches (2 samples), X-Y ratio outliers and samples with unbalanced ratios between methylated and unmethylated signals (9 samples). Moreover, we applied quality control functions implemented in minfi and discovered 1 additional methylation/unmethylation ratio outlier. To normalise the signal, we applied functional normalisation (Fortin et al., 2014), an approach that corrects for variation measured by the control probes on the array. Here, we considered the first 8 principal components explaining 95% of the variation in the 34 control probes. We removed probes on sex chromosomes, probes with detection pvalues of $p > 0.01$ in more than 5 % of the samples and previously reported cross-reactive probes (Chen et al., 2013; McCartney et al., 2016; Pidsley et al., 2016). Furthermore, we excluded probes that had been reported to overlap with common genetic variants (= polymorphic probes) as the signal of these probes might mirror genetic variation rather than true methylation signal (Chen et al., 2013). More specifically, we removed previously reported polymorphic methylation probes with common SNPs (MAF > 0.05, European population) directly located at the target methylated probe, at the single base

extension or on the probe body within 10 base pairs. Furthermore, we excluded samples that are technical replicates and that are not annotated to patients of cohorts 1-3*.* The resulting data comprised 401,870 methylation loci and 266 samples from 98 patients, including 90, 98 and 78 samples from low-grade osteoarthritis cartilage, high-grade osteoarthritis cartilage and synovium, respectively (Table S1). We conducted downstream statistical analyses on M-values as recommended (Du et al., 2010). To map methylation probe identifiers to their corresponding methylation sites and genomic positions (hg19) as well as close RefSeq genes, we used Illumina's annotation file version 1.2, provided by the R package IlluminaHumanMethylation450kanno.ilmn12.hg19.

DNA methylation data (replication set)
We included published methylation data for low-grade and high-grade osteoarthritis cartilage to replicate the findings of the EWAS and the ML-based classifiers (den Hollander et al., 2014). The data is publicly available in the Gene Expression Omnibus database (Edgar et al., 2002) and accessible through the entry number *GSE63106.* The replication data consists of matching low-grade and high-grade osteoarthritis cartilage samples from 31 patients who underwent total joint replacement to treat primary osteoarthritis (knee: 17 patients, hip: 14 patients). We downloaded the data matrix with the beta values per sample ("GSE63106_series_matrix.txt.gz") for 374,412 methylation sites. To ensure higher comparability, we converted these beta values to M-values and used them for all downstream analyses between discovery and replication analyses.

Genotype data
Genotypes were measured with the InfiniumCoreExome-12v1-1_A array or the InfiniumCoreExome-24v1-1_A array (supplemental subjects and methods). Genotype data were preprocessed as previously described (Steinberg et al., 2021).
Briefly, genotypes for *cohort1* were measured on the array InfiniumCoreExome-12v1-1_A array, *cohort2* and *cohort3* on the InfiniumCoreExome-24v1-1_A array. Genotype data were preprocessed as previously described (Steinberg et al., 2021). Briefly, we called the variants using the GenCall (Illumina) tool and mapped the genotypes to GRC37/hg19 by applying an online software (http://www.well.ox.ac.uk/~wrayner/strand/index.html). We then applied the same quality control procedure for both arrays. In short, we (1) removed samples and variants with a call rate < 90%, (2) correlated measured array genotypes to Fluidigm genotypes (no sample achieved a correlation < 0.95) and (3) removed samples using several filters (call rate < 98%, heterozygosity distribution outliers performed using 2 different minor allele frequency (MAF) bins with ≥ 1% MAF and < 1% MAF and sex discrepancies). Subsequently, we also conducted (4) pairwise identity by descent (IBD) analysis using PLINK software. Here, we only considered variants with MAF < 1% and a pruned dataset (linkage disequilibrium based pruning with R2 < 0.2). We further only kept patients with a pairwise PI_HAT ≤ 0.2. We then combined the data with the individuals from the 10000 Genomes Project and reduced the dataset to overlapping variants. By applying multidimensional scaling with PLINK and visually investigating the results of the first 2 components, we discovered and removed three ethnic outliers. Furthermore, we removed variants with a call rate < 98% or Hardy Weinberg p-value (pHWE) $<10^{-4}$.

We combined QCed data from both array types and applied a checking tool (http://www.well.ox.ac.uk/~wrayner/tools/; v4.2.7) to detect variants to be removed due to diverging strand, position and allele frequency information compared to the Haplotype Reference Consortium (HRC) panel. Subsequently, we performed imputation applying the Michigan imputation server (Das et al., 2016) (https://imputationserver.sph.umich.edu/index.html) with Eagle2 (v2.3) phasing and using the HRC panel(v1.1 2016) as reference. Finally, a post-imputation tool was applied (http://www.well.ox.ac.uk/~wrayner/tools/Post-Imputation.html; v1.0.2) to detect and remove genetic variants with unreliable imputation measures (R2 < 0.3, pHWE <1x10-4). In the end,

the considered genotype data comprised 10,249,108 autosomal variants for 98 patients for which methylation data is available.

Sample stratification using multivariate modelling

To investigate differences between tissues on a global level, we used DNA methylation data (including 98, 90 and 78 samples from low-grade and high-grade osteoarthritis cartilage and synovium, respectively) and removed batch effects using the ComBat function (Johnson et al., 2007) from the R package sva and considered these corrected methylation values for the multivariate models. We applied (1) principal component analysis (R function prcomp) and (2) a follow-up hierarchical clustering approach. To calculate the association between the second principal component (*PC2*) and cartilage grades, we built the following linear model: *PC2 ~ cartilage_type*.

For the hierarchical clustering approach, we applied the HCPC function from the R package FactoMineR (parameter settings: metric = "euclidean", method="ward") on the principal components (Lê et al., 2008).

Differential methylation analysis (discovery)

We sought to discover differentially methylated sites (DMSs) in pairs of low-grade and high-grade osteoarthritis cartilage samples from 90 patients. We performed linear modeling using the function lmFit and eBayes function of limma (Ritchie et al., 2015). We added the factor variable patient ID to ensure paired analysis design and additional 18 surrogate variables (SV) to account for technical confounders as covariates. The SV were estimated using the sva package by protecting the outcome variable tissue_state to conserve variation between low-grade osteoarthritis and high-grade osteoarthritis cartilage samples (Leek et al., 2012). The number of relevant SVs were estimated with the num.sv function (‘be’ method). To assess genome-wide significance in the EWAS, we applied Bonferroni correction considering the number of tested methylation sites: $0.05 / 401{,}870 = 1.24\text{x}10^{-7}$. The standard errors of the coefficients were estimated by multiplying the unscaled standard deviations of the coefficients (“stdev.unscaled”) with the standard deviations of residual variances after Bayes shrinkage (“s2.post”).

To quantify the overrepresentation of hypermethylated sites among DMSs, we performed a binomial test (Number of successes: 8,783, number of trails: 15,328, expected probability of success: 0.5, alternative hypothesis: “greater”).

To identify differentially methylated regions (DMRs), we applied the R package dmrff (Suderman et al., 2018) to the summary statistics of the EWAS including the beta coefficients, their respective standard errors and p values. We applied the default parameter setting for the dmrff function (maxgap = 500, p.cutoff = 0.05). Regions were defined as differentially methylated when being composed of more than 1 methylation site and achieving a Bonferroni adjusted $p < 0.05$.

To identify sex-specific markers of cartilage degeneration, we performed EWAS in paired low-grade and high-grade cartilage samples of female (n = 52) and male (n = 38) patients, separately. We applied the same approach as in the combined EWAS to identify DMS (number of SVs: 14 and 10 in female and male-specific EWAS, respectively).

Differential methylation analysis (replication)

We performed an EWAS on knee samples of the replication data (17 low-grade and high-grade osteoarthritis cartilage samples, respectively) to validate our findings. To determine DMSs between low- and high-grade osteoarthritis cartilage, we applied a mixed-effect model (using the R package *lme4*):

$$methyl\_site = tissue\_status + gender + age + slide + 2\ SVs + (1|ID)$$

with *methyl_site* denoting m-values of a specific methylation site and *tissue_status* discriminating between low-grade osteoarthritis and high-grade osteoarthritis cartilage. We

also included 2 surrogate variables (the number was estimated using the *leek* method in the num.sv function of the sva R package) and *slide* as fixed effects to correct for technical variation, whereas *(1|ID)* accounts for patient-specific variation as random effect. We applied this specific model because a similar approach has been applied to this dataset previously (den Hollander et al., 2015).

Replicated DMSs are defined as (1) showing the same direction of effect in the replication set (2) at nominal significance ($p < 0.05$). We calculated the pvalues using the the Kenward-Roger approximation. To test whether there is an overrepresentation of hypomethylated sites among replicated DMSs, we performed a binomial test (Number of successes: 3,961, number of trails: 7,192, expected probability of success: 0.5, alternative hypothesis: "greater").

We performed the EWAS on a regional level in the replication dataset with dmrff (default settings analog to the discovery analysis) by including summary statistics of the replication EWAS on methylation site level. We regard DMRs as replicated when they are composed of exactly the same sites in the replication set and show the same direction of effect on nominal significance ($p < 0.05$).

Pathway enrichment analysis

We applied the gometh and goregion functions (R package missMethyl) to identify enrichments among DMSs and DMRs (Maksimovic et al., 2021; Phipson et al., 2016). Across all approaches, we used Illumina's manifest file for the 450k array (version 1.2) to annotate the methylation probes (parameter "anno"). We only considered pathways consisting of between 20 and 200 genes. To identify enrichments among the DMS in the discovery analysis, we used the 15,328 DMS as query ("sig.cpg") and 401,870 methylation sites that passed the QC as background set. To investigate enrichments among replicated DMS, we used 7,192 replicated DMS as query and 346,288 methylation sites that passed the QC in the discovery and the replication set as background set. To examine enrichments among DMRs, we used 2,477 DMRs as query and 401,870 methylation sites that passed the QC in the discovery as background set.

Distinguishing cartilage grades using ML

To correct for technical variation in the methylation data (considering 98, 90 and 78 low-grade and high-grade osteoarthritis cartilage as well as synovium), we applied sva and estimated 25 relevant surrogate variables ('be' method) which we regressed out. We retained methylation samples from low-grade and high-grade osteoarthritis cartilage and standardized methylation values per site to reduce the influence of methylation site-specific variance.
Following up, we constructed classifiers that distinguish cartilage grades. We then trained and tested Random Forest (RF)-based classifiers repeatedly in 5-fold cross-validations (cv) in 25 iterations. Thus, we trained and tested 125 RF models (25 iterations * 5- fold cv) in total.

Per iteration, we divided the data randomly into five subsets, each comprising the same number of samples. Per fold, we then trained a Random Forest classifier on four subsets (= training set) and tested it on the hold-out set (= test set). To train one classifier, we conducted a two-step procedure:

(1) In the training data, we performed an EWAS using linear modelling (R function lmfit from the limma package) to assess the association between each methylation site and tissue status:

$$methylation \sim tissue\_status$$

Of the differentially methylated sites, we selected 1000 sites with the largest absolute effect size ($p < 0.05$/number of tested methylation sites). Expressly, we only used the training set here to avoid overfitting.

(2) We built Random Forest-based classifiers considering the set of preselected methylation sites from the previous step. Here, we used the trainControl function of the caret R package (parameter setting: method = "repeatedcv", number = 3, repeats = 3) to define the training procedure. To build the actual model, we used the train function (method = ‘rf’).

We tested the resulting classifier on the hold-out test set by comparing the actual and predicted degradation state (low-grade or high-grade osteoarthritis cartilage) and estimated their respective prediction accuracy.
Across all iterations and folds, we trained and tested 125 classifiers, in total. We then reported the mean (μ) and standard deviation (σ) of the respective accuracy values (n = 125).
We further calculated the 95% Confidence interval (95% CI) of these accuracies with:

$$95\%\ CI = \mu \pm 1.96\ \frac{\sigma}{\sqrt{125}}$$

In addition, we calculated the receiver operating characteristic (ROC)-curve (based on the classifier’s classification probabilities) and estimated its corresponding area under the curve (AUC).

Validating ML classifiers
We sought to further investigate the performance of ML classifiers more generally by training one RF-based classifier on our entire dataset (i.e. not to sample subsets as in cross-validation) and then validating this model in an external dataset. We trained the classifier on the entire patient cohort by applying the same two-step procedure as during cross-validation (First: Performing EWAS to select 1000 methylation sites, second: Constructing the RF model). Subsequently, we tested the prediction quality of the resulting classifiers on the validation dataset which we standardized per methylation site (analog to the data preparation in the discovery analysis). We then applied the classifiers and assessed their prediction quality separately in hip and knee samples. Prediction accuracies and their 95% confidence intervals were calculated with carets ConfusionMatrix-function.
We applied the same training-testing strategy to construct a classifier based on support vector machines (SVM; caret’s train function method parameter: ‘svmLinear2’) and gradient boosting machine (gbm; caret’s train function method parameter: ‘gbm’) to compare the performances of these machine learning models on the validation set. Furthermore, we prioritized 300 methylation sites based on the variable importance (mean decrease in node impurity) in the RF model and overlapped them with DMSs.

Identification of methylation quantitative trait loci
We performed genome-wide cis-methylation quantitative trait locus (mQTL) analysis in low-grade (97 samples), high-grade osteoarthritis cartilage (89 samples) as well as in synovium (78 samples), thus including only samples for which complete covariate information was available. We restricted our analyses to SNPs with a minor allele frequency > 0.05 (low-grade osteoarthritis cartilage: 5,382,160 SNPs, high-grade osteoarthritis cartilage: 5,418,639 SNPs, synovium: 5,407,053 SNPs). Furthermore, we defined the cis-distance with 1 Mb. We used the R package vcfR (Knaus and Grünwald, 2017) to read the genotype files and extract allele dosages. We conducted the mQTL analysis using the R package MatrixEQTL (Shabalin, 2012). We estimated the mQTL effects with linear models:

methylation = genotype + age + sex + sequencing_batch

Methylation and *genotype* denoting m-values of methylation sites and the allele dosage values of genetic variants (a continuous value ranging between 0 and 2), respectively. We also included sex, age and sequencing batch information of each sample (*sequencing_batch*) to

correct for biological and technical variation in DNA methylation data. We defined two thresholds to identify genome-wide significant methylation QTL effects:

(1) Bonferroni threshold: Genome-wide significance defined by p < 0.05/number of tested SNP-methylation site pairs (Low-grade osteoarthritis cartilage: $p < 3.05x10^{-11}$, high-grade osteoarthritis cartilage: $p < 3.03x10^{-11}$, synovium: $p < 3.03x10^{-11}$).
(2) False-discovery rate (FDR): We estimated the FDR of mQTL-effects using the MatrixEQTL package. It calculates the FDR considering the total number of tested cis-pairs per tissue.

Identification of sex-specific methylation quantitative trait loci

We performed cis-mQTL analysis (cis-distance: 1Mb) to identify sex-specific associations between methylation sites and SNPs per tissue (low-grade osteoarthritis cartilage: samples of 42 male and 55 female patients; high-grade osteoarthritis cartilage: 38 and 51; synovium: 29 and 49).

We considered SNPs with a MAF > 0.05 in samples of both sexes (SNP number in low-grade osteoarthritis cartilage: 4,927,666, high-grade osteoarthritis cartilage: 4,950,062, synovium: 5,062,551). Using the R package MatrixEQTL (Shabalin, 2012), we applied the following interaction model:

methylation = age + sequencing_batch + sex * genotype

Methylation and *genotype* are denoting m-values of methylation sites and the allele dosage values, respectively. The term *sex * genotype* refers to the interaction term to test for the equality of the genetic effect between samples of the two genders.

Characterizing the mQTL architecture in osteoarthritis tissues

To characterize the mQTL targeted methylation sites in low-grade (n = 10,639) and high-grade (n = 6,785) osteoarthritis cartilage as well as in synovium (n = 4,493), we used annotations of Illumina's annotation file (version 1.2). For the enrichments approaches, we used methylation sites that (1) passed QC and (2) are within 1Mb to a SNP (thus, can be potentially be targeted by a cis-mQTL effect) as background set (n = 401,844). We performed the actual enrichment by applying hypergeometric tests (R function phyper). We applied the Bonferroni correction (location in genes: p < 0.05/7 categories = 0.0071; relation to CpG island: p < 0.05/6 categories = 0.0083)

Differential mQTL effects in low-grade and high-grade osteoarthritis cartilage

To calculate differential mQTL-effects between low-grade and high-grade osteoarthritis cartilage, we used the software MetaTissue v0.5 (Sul et al., 2013), available under http://genetics.cs.ucla.edu/metatissue/download.html. Analogously to our genome-wide, tissue-specific approach to identify mQTLs, we included sex, age and sequencing_batch as covariates in these models. We used the MetaTissue software to calculate posterior probabilities (m-values) and focused on genetic variant-methylation site pairs with a significant effect in one tissue (m-value > 0.9), but not in the other (m-value < 0.1). We only considered variant-methylation site pairs with a genome-wide significant effect in either low-grade or high-grade osteoarthritis cartilage (Bonferroni correction).

Comparing joint methylation QTLs with a whole blood based meta-study

We compared the effects of significant mQTL associations (Bonferroni correction) that were estimated in osteoarthritis-relevant tissues (low-grade osteoarthritis cartilage, high-grade osteoarthritis cartilage and synovium) with the corresponding effects (mQTL effect between the same variant-methylation site pairs) of a mQTL meta-analysis of 36 cohorts in whole blood. From the whole blood mQTL meta-analysis, we considered results from the fixed effect models.

Summary statistics of genome-wide association studies

For the MR approach and the colocalisation analysis, we included summary statistics from three osteoarthritis-related phenotypes: (1) osteoarthritis at any site (all OA) and (2) knee osteoarthritis (knee OA) and (3) total knee replacement (TKR). Summary statistics for all OA and knee OA were previously published (Tachmazidou et al., 2019) and downloaded from the gwas catalog. Summary statistics for TKR were calculated by meta-analysing the arcOGEN and UKBB data using the METAL software (Willer et al., 2010).

Two-sample Mendelian randomization (2SMR)

To estimate putative causal effects of methylation on osteoarthritis in each of the three examined joint tissues (low-grade and high-grade osteoarthritis cartilage as well as for synovium), we applied 2SMR by integrating mQTL and GWAS data from three osteoarthritis traits (all OA, knee OA, TKR).

We performed 2SMR following the workflow implemented in the R package TwoSampleMR (version 0.4.25) (Hemani et al., 2018). We first prioritized 15,328 methylation sites with significantly different methylation levels between low-grade and high-grade osteoarthritis cartilage. For these methylation sites, we selected methylation site-variant pairs with a significant cis-mQTL association (FDR < 0.05). We then converted the available location-based SNP ids (*chr:pos_allele1_allele2*) to rs-ids using information from dbSNP (version 151). Subsequently, we performed clumping (r2 < 0.01) to filter for independent mQTLs per methylation site. For this step, we applied the function clump_data of the TwoSampleMR package and used the LD structure of the European population of the 1000 genomes project. We then extracted the associations of the relevant mQTL SNPs from the GWAS summary statistics for three outcome traits (all OA, knee OA, TKR).

To estimate the causal effect of hypermethylation at a specific site on osteoarthritis and thus simplify the interpretation of the MR results, we orientated the beta-coefficient of the mQTL association between a SNP and methylation site to a positive effect. Subsequently, we harmonized the data with the function dat_harmonize.

We then conducted MR by integrating methylation sites (= exposure), osteoarthritis relevant outcomes (= outcome) and mQTL (instrumental variables = IV). For methylation sites with exactly 1 IV, we applied the Wald-ratio, otherwise the inverse-variance-weighted (IVW) method.

In low-grade osteoarthritis cartilage, we tested 3,378 methylation sites for their putative causal effect on osteoarthritis (all OA = 3,378 methylation sites, knee OA = 3,378, TKR = 3,343). In high-grade osteoarthritis cartilage, we considered 2,042 methylation sites (all OA = 2,042, knee OA = 2,042, TKR = 2,026). In synovium, we investigated the effect of 1,561 methylation sites (all OA = 1,560, knee OA = 1,560, TKR = 1,542). In total, we tested 10,099, 6,110 and 4,662 methylation site-osteoarthritis trait combinations in low-grade, high-grade osteoarthritis cartilage and synovium, respectively. Per tissue, we used the Bonferroni method (0.05 / total number of tested methylation site-GWAS trait pairs) to correct for the number of conducted tests (low-grade osteoarthritis cartilage: $p < 4.95 \times 10^{-6}$, high-grade osteoarthritis cartilage: $p < 8.18 \times 10^{-6}$, synovium: $p < 1.07 \times 10^{-5}$).

We investigated the opposite direction of effect (osteoarthritis causal for methylation changes) for every tested methylation site-osteoarthritis trait combination, including osteoarthritis trait as exposure and methylation site as outcome into the MR approach. We used independent GWAS risk SNPs as IV. If a risk SNP was not directly available, we included a proxy SNP (with $r^2 > 0.8$) which we estimated based on the LD structure from the UK Biobank. In summary, we applied 27, 10 and 4 SNPs as IV for all OA, knee OA and TKR, respectively. We applied the IVW method.

Colocalisation analysis

We applied colocalisation analysis to estimate the overlap of the mQTL signals and GWAS osteoarthritis signals (Giambartolomei et al., 2014). We examined genome-wide signals for osteoarthritis at any site (all OA, 33 risk loci), knee osteoarthritis (knee OA, 12 risk loci) and

total knee replacement (TKR, 5 risk loci*)* using colocalisation. Statistically independent signals for all OA and knee OA were previously reported (Tachmazidou et al., 2019). To estimate independent signals for TKR, we performed clumping (--clump-kb: 1000, --clump-r2 0.1) on 31 genome-wide significant variants (p < 5e-8) with the PLINK software (version 1.9), here considering the LD structure from the UK Biobank. With this approach, we found five independent signals. We performed colocalisation by applying coloc.fast function (https://github.com/tobyjohnson/gtx/blob/526120435bb3e29c39fc71604eee03a371ec3753/R/coloc.R) using default settings. We conducted the colocalisation analysis separately for each GWAS phenotype and each tissue. To define genomic regions to be tested for colocalisation, we prioritized methylation loci with (1) at least one significant cis-mQTL (FDR < 0.05) and which are in close vicinity (<500 kb distance) to the index variant of a GWAS signal. We then performed colocalisation in the genomic region that is 1Mb upstream and downstream of the methylation site which corresponds to the region in which the cis mQTL analysis was performed. We considered genetic variants present in both the cis mQTL and the GWAS data. As in a previous study in which signals of GWAS for osteoarthritis were overlapped with eQTL results from osteoarthritis-affected tissues (Steinberg et al., 2021), we applied a posterior probability for a shared causal variant (PP4) of >=80% as a threshold indicating colocalisation. Annotated genes and locations of colocalised GWAS signals were extracted from Ensembl Variant Effect Predictor (http://grch37.ensembl.org/Homo_sapiens/Tools/VEP/).

Combining colocalisation results with eQTL data

We combined these colocalisation results with previously estimated eQTL data from the same patient cohort (Steinberg et al., 2021). More specifically, we tested whether the lead SNP of colocalised GWAS OA signals show an eQTL effect on relevant genes at nominal significance (p < 0.05). Here, relevant genes (1) are either directly annotated or (2) have a transcription start site within 20 kb to a methylation site for which *cis*-mQTLs colocalised with the GWAS risk locus.

We further tested whether there are associations between a methylation site and gene of the same region when both were linked to a GWAS risk locus (methylation site by colocalising mQTLs, gene by an eQTL effect of the GWAS lead SNP). We used previously published expression data of the same patient cohort in the same tissue types (low-grade osteoarthritis cartilage: 75 patients, high-grade osteoarthritis cartilage: 76, synovium: 70) (Steinberg et al., 2021). We applied the following linear models:

gene = methylation_site + sex + age + 15 peer_factors + seqbatches

with *gene* and *methylation_site* denoting gene expression values and m-values, respectively. *Peer_factors* refer to peer factors that were estimated in gene expression profiles in the respective tissue. *Seqbatches* denote the sequencing batches of the methylation samples.
For methylation site-gene pairs (four pairs and one pair in low-grade and high-grade osteoarthritis cartilage, respectively) for which we found an association at nominal significance (p < 0.05), we applied one sample MR to test for causal effects of methylation on gene expression levels (exposure: methylation, outcome: gene expression, instrument: mQTL) using the R package ivreg. Analog to the association analysis, we included sex, age, methylation sequencing batches and 15 gene expression PEER factors as covariates into these models.

Comparative analysis of colocalisation in joint and blood

We tested whether osteoarthritis-risk variant-methylation site pairs that colocalise using joint mQTL data also colocalise when overlapping osteoarthritis GWAS with whole blood mQTL data. For this colocalisation approach, we applied the same colocalisation method as performed on joint mQTL data. We applied a threshold of PP4 >=80% indicating colocalisation and a threshold of PP4 < 20% indicating no colocalisation.

To further increase the comparability between joint- and whole blood-based results, we performed a follow-up sensitivity analysis by conducting colocalisation between joint-mQTL and GWAS osteoarthritis signals on a reduced set of variants that are present in blood and joint mQTL data as well as in the relevant GWAS data. Using strict filtering, we then regarded colocalising GWAS and mQTL signals to be joint tissue-specific when achieving a PP4 >=80% in joint mQTL data on the (1) largest possible as well as on the (2) reduced variant set and when (3) achieving a PP4 < 20% when performing colocalisation between GWAS signals and blood mQTL data.

Supplemental references

Alvarez-Garcia, O., Fisch, K.M., Wineinger, N.E., Akagi, R., Saito, M., Sasho, T., Su, A.I., and Lotz, M.K. (2016). Increased DNA Methylation and Reduced Expression of Transcription Factors in Human Osteoarthritis Cartilage. Arthritis & Rheumatology *68*, 1876–1886. https://doi.org/10.1002/art.39643.

Aryee, M.J., Jaffe, A.E., Corrada-Bravo, H., Ladd-Acosta, C., Feinberg, A.P., Hansen, K.D., and Irizarry, R.A. (2014). Minfi: a flexible and comprehensive Bioconductor package for the analysis of Infinium DNA methylation microarrays. Bioinformatics *30*, 1363–1369. https://doi.org/10.1093/bioinformatics/btu049.

Aubourg, G., Rice, S.J., Bruce-Wootton, P., and Loughlin, J. (2021). Genetics of osteoarthritis. Osteoarthritis and Cartilage https://doi.org/10.1016/j.joca.2021.03.002.

Boer, C.G., Yau, M.S., Rice, S.J., Coutinho de Almeida, R., Cheung, K., Styrkarsdottir, U., Southam, L., Broer, L., Wilkinson, J.M., Uitterlinden, A.G., et al. (2021). Genome-wide association of phenotypes based on clustering patterns of hand osteoarthritis identify *WNT9A* as novel osteoarthritis gene. Ann Rheum Dis *80*, 367–375. https://doi.org/10.1136/annrheumdis-2020-217834.

Bonin, C.A., Lewallen, E.A., Baheti, S., Bradley, E.W., Stuart, M.J., Berry, D.J., van Wijnen, A.J., and Westendorf, J.J. (2016). Identification of Differentially Methylated Regions in New Genes Associated with Knee Osteoarthritis. Gene *576*, 312–318. https://doi.org/10.1016/j.gene.2015.10.037.

Chen, Y., Lemire, M., Choufani, S., Butcher, D.T., Grafodatskaya, D., Zanke, B.W., Gallinger, S., Hudson, T.J., and Weksberg, R. (2013). Discovery of cross-reactive probes and polymorphic CpGs in the Illumina Infinium HumanMethylation450 microarray. Epigenetics *8*, 203–209. https://doi.org/10.4161/epi.23470.

Das, S., Forer, L., Schönherr, S., Sidore, C., Locke, A.E., Kwong, A., Vrieze, S.I., Chew, E.Y., Levy, S., McGue, M., et al. (2016). Next-generation genotype imputation service and methods. Nat Genet *48*, 1284–1287. https://doi.org/10.1038/ng.3656.

Du, P., Zhang, X., Huang, C.-C., Jafari, N., Kibbe, W.A., Hou, L., and Lin, S.M. (2010). Comparison of Beta-value and M-value methods for quantifying methylation levels by microarray analysis. BMC Bioinformatics *11*, 587. https://doi.org/10.1186/1471-2105-11-587.

Durinck, S., Spellman, P.T., Birney, E., and Huber, W. (2009). Mapping identifiers for the integration of genomic datasets with the R/Bioconductor package biomaRt. Nat Protoc *4*, 1184–1191. https://doi.org/10.1038/nprot.2009.97.

Eckhardt, F., Lewin, J., Cortese, R., Rakyan, V.K., Attwood, J., Burger, M., Burton, J., Cox, T.V., Davies, R., Down, T.A., et al. (2006). DNA methylation profiling of human chromosomes 6, 20 and 22. Nat Genet *38*, 1378–1385. https://doi.org/10.1038/ng1909.

Edgar, R., Domrachev, M., and Lash, A.E. (2002). Gene Expression Omnibus: NCBI gene expression and hybridization array data repository. Nucleic Acids Res *30*, 207–210. https://doi.org/10.1093/nar/30.1.207.
Fortin, J.-P., Labbe, A., Lemire, M., Zanke, B.W., Hudson, T.J., Fertig, E.J., Greenwood, C.M., and Hansen, K.D. (2014). Functional normalization of 450k methylation array data improves replication in large cancer studies. Genome Biol *15*, 503. https://doi.org/10.1186/s13059-014-0503-2.
Fortin, J.-P., Triche, T.J., and Hansen, K.D. (2017). Preprocessing, normalization and integration of the Illumina HumanMethylationEPIC array with minfi. Bioinformatics *33*, 558–560. https://doi.org/10.1093/bioinformatics/btw691.
Giambartolomei, C., Vukcevic, D., Schadt, E.E., Franke, L., Hingorani, A.D., Wallace, C., and Plagnol, V. (2014). Bayesian test for colocalisation between pairs of genetic association studies using summary statistics. PLoS Genet *10*, e1004383. https://doi.org/10.1371/journal.pgen.1004383.
Hemani, G., Zheng, J., Elsworth, B., Wade, K.H., Haberland, V., Baird, D., Laurin, C., Burgess, S., Bowden, J., Langdon, R., et al. (2018). The MR-Base platform supports systematic causal inference across the human phenome. ELife *7*, e34408. https://doi.org/10.7554/eLife.34408.
den Hollander, W., Ramos, Y.F.M., Bos, S.D., Bomer, N., van der Breggen, R., Lakenberg, N., de Dijcker, W.J., Duijnisveld, B.J., Slagboom, P.E., Nelissen, R.G.H.H., et al. (2014). Knee and hip articular cartilage have distinct epigenomic landscapes: implications for future cartilage regeneration approaches. Ann Rheum Dis *73*, 2208–2212. https://doi.org/10.1136/annrheumdis-2014-205980.
den Hollander, W., Ramos, Y.F.M., Bomer, N., Elzinga, S., van der Breggen, R., Lakenberg, N., de Dijcker, W.J., Suchiman, H.E.D., Duijnisveld, B.J., Houwing-Duistermaat, J.J., et al. (2015). Transcriptional associations of osteoarthritis-mediated loss of epigenetic control in articular cartilage. Arthritis Rheumatol *67*, 2108–2116. https://doi.org/10.1002/art.39162.
Johnson, W.E., Li, C., and Rabinovic, A. (2007). Adjusting batch effects in microarray expression data using empirical Bayes methods. Biostatistics *8*, 118–127. https://doi.org/10.1093/biostatistics/kxj037.
Knaus, B.J., and Grünwald, N.J. (2017). vcfr: a package to manipulate and visualize variant call format data in R. Mol Ecol Resour *17*, 44–53. https://doi.org/10.1111/1755-0998.12549.
Lê, S., Josse, J., and Husson, F. (2008). FactoMineR: An R Package for Multivariate Analysis. Journal of Statistical Software *25*, 1–18. https://doi.org/10.18637/jss.v025.i01.
Leek, J.T., Johnson, W.E., Parker, H.S., Jaffe, A.E., and Storey, J.D. (2012). The sva package for removing batch effects and other unwanted variation in high-throughput experiments. Bioinformatics *28*, 882–883. https://doi.org/10.1093/bioinformatics/bts034.
Maksimovic, J., Oshlack, A., and Phipson, B. (2021). Gene set enrichment analysis for genome-wide DNA methylation data. Genome Biology *22*, 173. https://doi.org/10.1186/s13059-021-02388-x.
McCartney, D.L., Walker, R.M., Morris, S.W., McIntosh, A.M., Porteous, D.J., and Evans, K.L. (2016). Identification of polymorphic and off-target probe binding sites on the Illumina Infinium MethylationEPIC BeadChip. Genom Data *9*, 22–24. https://doi.org/10.1016/j.gdata.2016.05.012.
Min, J.L., Hemani, G., Davey Smith, G., Relton, C., and Suderman, M. (2018). Meffil: efficient normalization and analysis of very large DNA methylation datasets. Bioinformatics *34*, 3983–3989. https://doi.org/10.1093/bioinformatics/bty476.

Min, J.L., Hemani, G., Hannon, E., Dekkers, K.F., Castillo-Fernandez, J., Luijk, R., Carnero-Montoro, E., Lawson, D.J., Burrows, K., Suderman, M., et al. (2021). Genomic and phenotypic insights from an atlas of genetic effects on DNA methylation. Nat Genet *53*, 1311–1321. https://doi.org/10.1038/s41588-021-00923-x.
Phipson, B., Maksimovic, J., and Oshlack, A. (2016). missMethyl: an R package for analyzing data from Illumina's HumanMethylation450 platform. Bioinformatics *32*, 286–288. https://doi.org/10.1093/bioinformatics/btv560.
Pidsley, R., Zotenko, E., Peters, T.J., Lawrence, M.G., Risbridger, G.P., Molloy, P., Van Djik, S., Muhlhausler, B., Stirzaker, C., and Clark, S.J. (2016). Critical evaluation of the Illumina MethylationEPIC BeadChip microarray for whole-genome DNA methylation profiling. Genome Biol *17*, 208. https://doi.org/10.1186/s13059-016-1066-1.
Ritchie, M.E., Phipson, B., Wu, D., Hu, Y., Law, C.W., Shi, W., and Smyth, G.K. (2015). limma powers differential expression analyses for RNA-sequencing and microarray studies. Nucleic Acids Res *43*, e47. https://doi.org/10.1093/nar/gkv007.
Rushton, M.D., Reynard, L.N., Barter, M.J., Refaie, R., Rankin, K.S., Young, D.A., and Loughlin, J. (2014). Characterization of the Cartilage DNA Methylome in Knee and Hip Osteoarthritis. Arthritis & Rheumatology *66*, 2450–2460. https://doi.org/10.1002/art.38713.
Shabalin, A.A. (2012). Matrix eQTL: ultra fast eQTL analysis via large matrix operations. Bioinformatics *28*, 1353–1358. https://doi.org/10.1093/bioinformatics/bts163.
Steinberg, J., and Zeggini, E. (2016). Functional genomics in osteoarthritis: Past, present, and future. J Orthop Res *34*, 1105–1110. https://doi.org/10.1002/jor.23296.
Steinberg, J., Ritchie, G.R.S., Roumeliotis, T.I., Jayasuriya, R.L., Clark, M.J., Brooks, R.A., Binch, A.L.A., Shah, K.M., Coyle, R., Pardo, M., et al. (2017). Integrative epigenomics, transcriptomics and proteomics of patient chondrocytes reveal genes and pathways involved in osteoarthritis. Sci Rep *7*, 8935. https://doi.org/10.1038/s41598-017-09335-6.
Steinberg, J., Southam, L., Roumeliotis, T.I., Clark, M.J., Jayasuriya, R.L., Swift, D., Shah, K.M., Butterfield, N.C., Brooks, R.A., McCaskie, A.W., et al. (2021). A molecular quantitative trait locus map for osteoarthritis. Nature Communications *12*, 1309. https://doi.org/10.1038/s41467-021-21593-7.
Suderman, M., Staley, J.R., French, R., Arathimos, R., Simpkin, A., and Tilling, K. (2018). dmrff: identifying differentially methylated regions efficiently with power and control. BioRxiv 508556. https://doi.org/10.1101/508556.
Sul, J.H., Han, B., Ye, C., Choi, T., and Eskin, E. (2013). Effectively identifying eQTLs from multiple tissues by combining mixed model and meta-analytic approaches. PLoS Genet *9*, e1003491. https://doi.org/10.1371/journal.pgen.1003491.
Tachmazidou, I., Hatzikotoulas, K., Southam, L., Esparza-Gordillo, J., Haberland, V., Zheng, J., Johnson, T., Koprulu, M., Zengini, E., Steinberg, J., et al. (2019). Identification of new therapeutic targets for osteoarthritis through genome-wide analyses of UK Biobank data. Nature Genetics *51*, 230–236. https://doi.org/10.1038/s41588-018-0327-1.
Willer, C.J., Li, Y., and Abecasis, G.R. (2010). METAL: fast and efficient meta-analysis of genomewide association scans. Bioinformatics *26*, 2190–2191. https://doi.org/10.1093/bioinformatics/btq340.
Zhang, Y., Fukui, N., Yahata, M., Katsuragawa, Y., Tashiro, T., Ikegawa, S., and Michael Lee, M.T. (2016). Genome-wide DNA methylation profile implicates potential cartilage regeneration at the late stage of knee osteoarthritis. Osteoarthritis and Cartilage *24*, 835–843. https://doi.org/10.1016/j.joca.2015.12.013.

Email addresses:
Peter Kreitmaier: peter.kreitmaier@helmholtz-muenchen.de
Matthew Suderman: matthew.suderman@bristol.ac.uk
Lorraine Southam: lorraine.southam@helmholtz-muenchen.de
Rodrigo Coutinho de Almeida: R.Coutinho_de_Almeida@lumc.nl
Konstantinos Hatzikotoulas: hatzikotoulas@helmholtz-muenchen.de
Ingrid Meulenbelt: i.meulenbelt@lumc.nl
Julia Steinberg: Julia.Steinberg@nswcc.org.au
Caroline Relton: Caroline.Relton@bristol.ac.uk
J Mark Wilkinson: j.m.wilkinson@sheffield.ac
Eleftheria Zeggini: eleftheria.zeggini@helmholtz-muenchen.de
