# Supplementary material for: An epigenome-wide view of osteoarthritis in primary tissues
Source: Am J Hum Genet. 2022 Jun 8;109(7):1255–71. doi: 10.1016/j.ajhg.2022.05.010 (PMC9300761; doi:10.1016/j.ajhg.2022.05.010)
Supplement: Document S3. Article plus supplemental information [file mmc3.pdf]

An epigenome-wide view of osteoarthritis in primary tissues

Authors

Peter Kreitmaier, Matthew Suderman, Lorraine Southam, ..., Caroline L. Relton, J. Mark Wilkinson, Eleftheria Zeggini

Correspondence

j.m.wilkinson@sheffield.ac.uk (J.M.W.), eleftheria.zeggini@helmholtz-muenchen.de (E.Z.)

Kreitmaier et al., 2022, The American Journal of Human Genetics *109*, 1255–1271
July 7, 2022 © 2022 The Author(s).
https://doi.org/10.1016/j.ajhg.2022.05.010

CellPress

An epigenome-wide view of osteoarthritis in primary tissues

Peter Kreitmaier,[1,2] Matthew Suderman,[3] Lorraine Southam,[1] Rodrigo Coutinho de Almeida,[4] Konstantinos Hatzikotoulas,[1] Ingrid Meulenbelt,[4] Julia Steinberg,[1,5] Caroline L. Relton,[3] J. Mark Wilkinson,[6,8,*] and Eleftheria Zeggini[1,7,8,*]

Summary

Osteoarthritis is a complex degenerative joint disease. Here, we investigate matched genotype and methylation profiles of primary chondrocytes from macroscopically intact (low-grade) and degraded (high-grade) osteoarthritis cartilage and from synoviocytes collected from 98 osteoarthritis-affected individuals undergoing knee replacement surgery. We perform an epigenome-wide association study of knee cartilage degeneration and report robustly replicating methylation markers, which reveal an etiologic mechanism linked to the migration of epithelial cells. Using machine learning, we derive methylation models of cartilage degeneration, which we validate with 82% accuracy in independent data. We report a genome-wide methylation quantitative trait locus (mQTL) map of articular cartilage and synovium and identify 18 disease-grade-specific mQTLs in osteoarthritis cartilage. We resolve osteoarthritis GWAS loci through causal inference and colocalization analyses and decipher the epigenetic mechanisms that mediate the effect of genotype on disease risk. Together, our findings provide enhanced insights into epigenetic mechanisms underlying osteoarthritis in primary tissues.

Introduction

Osteoarthritis (MIM: 165720) is a complex degenerative joint disease characterized by chronic pain and stiffness. It affects more than 40% of people over the age of 70 and is a leading cause of disability worldwide.[1] In spite of its high prevalence, treatment methods are limited to pain management and total joint replacement (TJR). To drive the development of novel and personalized treatments, it is necessary to understand the genetic and genomic architecture underlying osteoarthritis. Genome-wide association studies (GWASs) have determined around 150 independent osteoarthritis-linked single-nucleotide variants.[2] For the most part, it is unknown which variants and genes at these loci are causal to disease development and along which molecular pathways they exert their osteoarthritis-promoting effect. To identify these mechanisms, studies using relevant tissues are necessary, and TJR surgeries provide an opportunity to molecularly profile relevant tissues from osteoarthritis-affected individuals.[3]

DNA methylation in promoter regions and particularly around the transcription start site is strongly associated with gene downregulation, whereas its effect in gene bodies or other regulatory regions remains less predictable. DNA methylation is dynamic, with highly tissue-specific patterns,[4] and can interact with a multitude of factors such as genotype, age, sex, or environment.[5] The methylation profiles of relevant tissues and cell types in complex diseases can further our understanding of disease etiology, for example by generating insights into perturbed regulatory mechanisms and by revealing epigenetic markers of disease development or progression. Given the importance of tissue-specific molecular patterns, initiatives such as GTEx,[6] ENCODE,[7] ROADMAP,[8] and BLUEPRINT[9] have generated large publicly available resources that have made molecular datasets broadly accessible. However, these datasets do not include osteoarthritis-affected tissues.

To fill this gap, a small number of studies have investigated DNA methylation profiles of articular cartilage, typically comparing methylation profiles between macroscopically intact (low-grade) and degraded (high-grade) osteoarthritis cartilage to identify epigenetic markers of cartilage degeneration. Previous epigenome-wide association studies (EWASs) of this type have been limited in size, with a maximum of 17 knee osteoarthritis-affected individuals studied to date.[10–14] There is a need for better powered studies to improve our understanding of the role of DNA methylation in osteoarthritis (supplemental note S1).

Combining DNA methylation data with matched genotypes enables the detection of genetic variants associated with differential methylation levels at cytosine-guanine dinucleotides (CpGs), i.e., methylation quantitative trait loci (mQTLs). Characterizing these associations can help elucidate effector genes through which disease-associated genetic risk variants may exert their biological effect. To date, studies seeking to investigate mQTL effects in joint

[1]Institute of Translational Genomics, Helmholtz Zentrum München, German Research Center for Environmental Health, 85764 Neuherberg, Germany; [2]Graduate School of Experimental Medicine, TUM School of Medicine, Technical University of Munich, 81675 Munich, Germany; [3]MRC Integrative Epidemiology Unit, Population Health Sciences, University of Bristol, Bristol BS8 2BN, UK; [4]Department of Biomedical Data Sciences, Section Molecular Epidemiology, Leiden University Medical Center, 2333 ZC Leiden, the Netherlands; [5]The Daffodil Centre, The University of Sydney, a Joint Venture with Cancer Council NSW, Sydney, NSW 1340, Australia; [6]Department of Oncology and Metabolism, The University of Sheffield, Sheffield S10 2RX, UK; [7]TUM School of Medicine, Technical University of Munich and Klinikum Rechts der Isar, 81675 Munich, Germany
[8]These authors contributed equally
*Correspondence: j.m.wilkinson@sheffield.ac.uk (J.M.W.), eleftheria.zeggini@helmholtz-muenchen.de (E.Z.)
https://doi.org/10.1016/j.ajhg.2022.05.010.
© 2022 The Author(s). This is an open access article under the CC BY license (http://creativecommons.org/licenses/by/4.0/).

tissues have mostly focused on a candidate gene[15] or single genetic variants previously linked to osteoarthritis.[16–19] One study has investigated the association of genome-wide methylation with gene expression in osteoarthritis-affected cartilage in 31 osteoarthritis-affected individuals (17 knee and 14 hip osteoarthritis patients).[11] They reported 87 methylation sites that were correlated with the expression of 70 genes, where both gene and methylation site were linked to cartilage degeneration. Of these, 36 were targeted by *cis*-mQTLs. There remains a need to comprehensively map the mQTL landscape on a genome-wide scale and, in better-powered sample sizes, to generate comprehensive insights into the interplay between genetic variation and epigenetic changes in osteoarthritis tissues, and to provide a resource to help elucidate the mechanism for novel genetic risk loci discovered in GWASs.

To date, molecular studies of osteoarthritis have mainly focused on articular cartilage, the most prominent osteoarthritis-affected tissue. However, osteoarthritis is regarded as a disease of the whole joint, affecting multiple tissues within the synovial joint. Therefore, expanding genomic analyses to include other joint tissues has the potential to reveal novel insights into disease progression. The synovium, a connective tissue that lines the joint capsule separating the synovial cavity from neighboring tissues, undergoes pathological alterations during osteoarthritis. There is well-documented evidence of synovial inflammation in osteoarthritis-affected joints, referred to as synovitis.[20] Several studies have compared mQTL effects across tissues but have focused only on specific, osteoarthritis-linked loci.[16,19,21,22]

In this study, we have analyzed genome-wide methylation profiles from up to 98 osteoarthritis-affected individuals undergoing TJR due to knee osteoarthritis (matched low-grade and high-grade cartilage and synovium). We enhance our understanding of osteoarthritis aetiopathogenesis by (1) identifying methylation markers for cartilage degeneration, (2) building machine-learning-based models to distinguish between low-grade and high-grade osteoarthritis cartilage samples, (3) determining genome-wide methylation quantitative trait loci (mQTLs) in osteoarthritis tissues (cartilage and synovium), and (4) resolving high-confidence effector genes for osteoarthritis GWAS signals.

Subjects and methods

For full details of methods, see supplemental subjects and methods.

Osteoarthritis-affected individuals and study samples

Samples from osteoarthritis-affected knees were collected in 101 osteoarthritis-affected individuals that underwent total knee replacement due to late-stage osteoarthritis. Cartilage samples were graded with the OARSI cartilage classification system (cohort1) or International Cartilage Repair Society (ICRS) scoring system (cohort2 and cohort3). This work was approved by Oxford NHS REC C (10/H0606/20 and 15/SC/0132), and samples were collected under Human Tissue Authority license 12182, Sheffield Musculoskeletal Biobank, University of Sheffield, UK. Before participating in the study, all osteoarthritis-affected individuals provided written, informed consent.

Sample extraction

A previous study[3] reported the isolation of the chondrocytes (section "Isolation of chondrocytes"), the isolation of synoviocytes (section "Isolation of synoviocytes"), and DNA extraction (section "DNA, RNA and protein extraction") in its methods part.

DNA methylation data

Genome-wide DNA methylation was measured with the Illumina 450k or EPIC array in three sequencing batches. We used the R package minfi to read idat files.[23,24] We removed samples of three ethnicity outliers, gender mismatches (two samples), X-Y ratio outliers, and samples with unbalanced ratios between methylated and unmethylated signals (ten samples). To normalize methylation signals, we applied functional normalization,[25] We removed probes on sex chromosomes, probes with detection p values of $p > 0.01$ in more than 5% of the samples, and previously reported cross-reactive probes.[26–28] Furthermore, we excluded probes that had been reported to overlap with common genetic variants, as the signal of these probes might solely reflect genetic variation rather than true methylation signal.[26] The resulting data comprised 401,870 methylation loci and 266 samples from 98 osteoarthritis-affected individuals (56 female and 42 male patients, age range: 38–88, age mean: 69.6, age sd: 9.72, Table S1), including 98, 90, and 78 samples from low-grade osteoarthritis cartilage, high-grade osteoarthritis cartilage, and synovium, respectively. We conducted downstream statistical analyses on M values as recommended.[29]

DNA methylation data (replication set)

We used published methylation data for low-grade and high-grade osteoarthritis cartilage to replicate the findings of the EWAS and the machine-learning-based classifiers.[30]

The data is publicly available in the Gene Expression Omnibus database[31] and accessible through the entry number GEO: GSE63106. The replication data comprises methylation data of matching low-grade and high-grade osteoarthritis cartilage samples from 31 patients who underwent total joint replacement to treat primary osteoarthritis (knee: 17 osteoarthritis-affected individuals, hip: 14 osteoarthritis-affected individuals).

Genotype data

Genotypes were measured with the InfiniumCoreExome-12v1-1_A array or the InfiniumCoreExome-24v1-1_A array (supplemental subjects and methods). Genotype data were preprocessed as previously described.[3]

Sample stratification with multivariate modelling

To investigate differences between tissues on a global level, we used DNA methylation data (including 98, 90, and 78 samples from low-grade and high-grade osteoarthritis cartilage and synovium, respectively) corrected for batch effects with the ComBat function[32] from the R package sva and considered these corrected methylation. We applied (1) principal-component analysis (R function prcomp) and (2) a follow-up hierarchical clustering (R package FactoMineR).[33]

Differential methylation analysis (discovery)

To identify differentially methylated sites (DMSs) in pairs of low-grade and high-grade osteoarthritis cartilage samples from 90

osteoarthritis-affected individuals, we performed linear modeling by using the function lmFit and eBayes function of limma.[34] We added the factor variable patient ID to ensure paired analysis design and 18 surrogate variables (SVs) to account for technical confounders as covariates. To assess genome-wide significance in the EWAS, we applied Bonferroni correction considering the number of tested methylation sites: $0.05/401{,}870 = 1.24 \times 10^{-7}$. To identify differentially methylated regions (DMRs), we applied the R package dmrff.[35] Regions were defined as differentially methylated when composed of more than one methylation site and achieving a Bonferroni-adjusted $p < 0.05$. To identify sex-specific markers of cartilage degeneration, we used a similar approach as in the combined analysis (supplemental subjects and methods).

Differential methylation analysis (replication)

We performed an EWAS on knee samples of the replication data (17 low-grade and high-grade osteoarthritis cartilage samples, respectively) to validate our findings. To determine DMSs between low- and high-grade osteoarthritis cartilage, we applied a mixed-effect model, which is similar to what has been applied to this dataset previously[11] (supplemental subjects and methods). Replicated DMSs are defined as (1) showing the same direction of effect in the replication set (2) at nominal significance ($p < 0.05$). We performed the EWAS on a regional level in the replication dataset with dmrff (default settings analog to the discovery analysis). We defined DMRs as replicated when they are composed of exactly the same methylation sites in the replication set and show the same direction of effect on nominal significance.

Pathway enrichment analysis

We used the gometh and goregion functions (available through R package missMethyl) to identify enrichments among DMSs and DMRs.[36,37] We considered pathways consisting of between 20 and 200 genes.

Distinguishing cartilage grades with machine learning

We constructed classifiers that distinguish cartilage grades. More specifically, we trained and tested random forest (RF)-based classifiers repeatedly in 5-fold cross validations (cv) in 25 iterations (R package caret). In total, we trained and tested 125 RF models (25 iterations × 5-fold cv) (supplemental subjects and methods). To validate our approach, we trained RF-, support-vector-machine-, and gradient-boosting-machine-based classifiers on our entire dataset and tested the prediction quality of the resulting classifiers on the validation dataset. We then applied the classifiers and assessed their prediction quality separately in hip and knee samples. Prediction accuracies and their 95% confidence intervals were calculated with carets ConfusionMatrix-function.

Identification of methylation quantitative trait loci

We performed genome-wide *cis*-methylation quantitative trait locus (mQTL) analysis in low-grade (97 samples) and high-grade osteoarthritis cartilage (89 samples) as well as in synovium (78 samples), thus including only samples for which complete covariate information was available. We restricted our analyses to SNPs with a minor allele frequency > 0.05. Furthermore, we defined the *cis*-distance with 1 Mb. We conducted the mQTL analysis by using the R package MatrixEQTL.[38] We applied linear models and corrected for age, sex, and batch effects (supplemental subjects and methods). We defined two thresholds to identify genome-wide-significant methylation QTL effects.

(1) Bonferroni threshold: genome-wide significance defined by $p < 0.05$/number of tested SNP-methylation site pairs (low-grade osteoarthritis cartilage: $p < 3.05 \times 10^{-11}$, high-grade osteoarthritis cartilage: $p < 3.03 \times 10^{-11}$, synovium: $p < 3.03 \times 10^{-11}$).
(2) False discovery rate (FDR): we estimated the FDR of mQTL effects by using the MatrixEQTL package. It calculates the FDR considering the total number of tested *cis*-pairs per tissue.

To characterize mQTL architecture in osteoarthritis tissues, we used methylation site annotations of Illumina's annotation file (version 1.2). For the enrichment approaches, we applied hypergeometric tests (R function phyper). To identify sex-specific *cis*-mQTLs, we applied MatrixEQTL by using an interaction model (supplemental subjects and methods).

Differential mQTL effects in low-grade and high-grade osteoarthritis cartilage

To calculate differential mQTL effects between low-grade and high-grade osteoarthritis cartilage, we used the software MetaTissue v0.5 (see web resources).[39] Analogously to our genome-wide, tissue-specific approach to identify mQTLs, we included sex, age, and sequencing batches as covariates in these models. We used the MetaTissue software to calculate posterior probabilities (m values) and focused on genetic variant-methylation site pairs with a significant effect in one tissue (m value > 0.9) but not in the other (m value < 0.1).

Comparing joint with whole blood methylation QTLs

We compared mQTL effects (Bonferroni correction) of joint tissues (low-grade osteoarthritis cartilage, high-grade osteoarthritis cartilage, and synovium) with the corresponding effects (mQTL effect between the same variant-methylation site pairs) of a mQTL meta-analysis (Genetics of DNA Methylation Consortium, see web resources) of 36 cohorts in whole blood.[40] We considered results from the fixed-effect models from the whole blood mQTL meta-analysis.

Summary statistics of GWASs

For the MR approach and the colocalization analysis, we included summary statistics from three osteoarthritis-related phenotypes: (1) osteoarthritis at any site (all OA) and (2) knee osteoarthritis (knee OA) and (3) total knee replacement (TKR). Summary statistics for all OA and knee OA were previously published[41] and downloaded from the GWAS Catalog. We calculated summary statistics for TKR by meta-analyzing the arcOGEN and UKBB data with the METAL software.[42]

Two-sample Mendelian randomization

To estimate putative causal effects of methylation, we applied two-sample Mendelian randomization (2SMR) by integrating mQTL data of the three examined joint tissues and GWAS data from three osteoarthritis traits (all OA, knee OA, and TKR). We performed 2SMR following the workflow implemented in the R package TwoSampleMR (version 0.4.25).[43] In low-grade osteoarthritis cartilage, we tested 3,378 methylation sites for their putative causal effect on osteoarthritis (all OA = 3,378 methylation sites, knee OA = 3,378, and TKR = 3,343). In high-grade osteoarthritis cartilage, we considered 2,042 methylation sites (all OA = 2,042, knee OA = 2,042, and TKR = 2,026). In synovium, we investigated the effect of 1,561 methylation sites (all OA = 1,560, knee OA = 1,560, and TKR = 1,542). In total, we tested 10,099, 6,110, and 4,662

methylation site-osteoarthritis trait combinations in low-grade and high-grade osteoarthritis cartilage and synovium, respectively. Per tissue, we applied the Bonferroni method to correct for the number of performed tests (low-grade osteoarthritis cartilage: $p < 4.95 \times 10^{-6}$, high-grade osteoarthritis cartilage: $p < 8.18 \times 10^{-6}$, synovium: $p < 1.07 \times 10^{-5}$).

We investigated the opposite direction of effect (osteoarthritis causal for methylation changes) for every tested methylation site-osteoarthritis trait combination (using R package TwoSampleMR). We used 27, 10, and 4 SNPs as instrumental variable (IV) for all OA, knee OA, and TKR, respectively. Here, we applied the inverse-variance-weighted (IVW) method.

Colocalization analysis

We applied colocalization analysis to statistically estimate the overlap of mQTL signals in the three osteoarthritis tissues and GWAS signals.[44] We examined genome-wide signals for osteoarthritis at any site (all OA, 33 risk loci), knee osteoarthritis (knee OA, 12 risk loci), and total knee replacement (TKR, 5 risk loci). We performed colocalization by applying coloc.fast function (web resources). We conducted the colocalization analysis separately for each GWAS osteoarthritis trait and each tissue (supplemental subjects and methods). We used a posterior probability threshold for having a shared causal variant ("PP4") of ≥80% (thus indicating colocalization) as previously applied.[3] Annotated genes and locations of colocalized GWAS signals were extracted from Ensembl Variant Effect Predictor (see web resources).

Combining colocalization results with eQTL and gene expression data

We combined these colocalization results with previously estimated eQTL data from the same patient cohort.[3] More specifically, we tested whether the lead SNP of colocalized GWAS OA signals show an eQTL effect on local genes at nominal significance ($p < 0.05$). We used previously published, matching expression data (low-grade osteoarthritis cartilage: 75 osteoarthritis-affected individuals, high-grade osteoarthritis cartilage: 76, synovium: 70)[3] of the same osteoarthritis-affected individuals in the same tissue types to test associations between osteoarthritis-linked methylation sites and genes in the same region. We estimated associations between methylation and gene expression by using linear models. We estimated putative causal effects of methylation on gene expression by using one-sample MR with the R package ivreg (supplemental subjects and methods).

Comparative analysis of colocalization in joint and blood

We tested whether osteoarthritis-risk variant-methylation site pairs that colocalize using joint mQTL data also colocalize when overlapping osteoarthritis GWASs with whole blood mQTL data. For this colocalization approach, we applied the same colocalization method as performed on joint mQTL data. We applied a threshold of PP4 ≥ 80% and PP4 < 20% indicating colocalization and no colocalization, respectively.

Results

Methylation profiles differ between tissue types and disease grades

To describe distinct methylation profiles in three tissue types (low-grade and high-grade osteoarthritis cartilage and synovium), we first assessed whether tissue and osteoarthritis grade have strong, systematic effects on global variations in the epigenome. We used principal-component analysis to examine variation in global methylation profiles and observed a clear separation between synovium and cartilage samples along the first principal component (PC) and partly overlapping clustering between low-grade and high-grade osteoarthritis cartilage along the second PC (Figure 1A). A linear model confirmed the significant association between the second PC and cartilage grades ($p = 1.51 \times 10^{-16}$, beta = 253.79, SE = 27.93). Using a hierarchical clustering approach, we observed stratification by tissue type and cartilage degradation state (Figure 1B).

EWAS reveals widespread, robustly replicating signals

To identify DNA methylation markers of cartilage degeneration, we performed an EWAS on paired low-grade and high-grade osteoarthritis cartilage samples from 90 osteoarthritis-affected individuals across 401,870 methylation sites (supplemental note S2). We identified 15,328 differentially methylated sites (DMSs) distributed across the whole genome (Figures 2A [upper panel], 2B, and 2C and Table S2) by using a significance threshold of $p < 1.24 \times 10^{-7}$ (subjects and methods). Furthermore, we identified 2,477 differentially (Bonferroni-adjusted $p < 0.05$) methylated regions (DMRs) (Figure 2A [bottom panel], Figure S1, and Table S3).

To biologically characterize the DMS, we performed enrichment analyses and identified 29 and 4 Gene Ontology (GO) and Kyoto Encyclopedia of Genes and Genomes (KEGG) terms, respectively (Figure 2D, Figure S2, and Table S4), including pathways linked to osteoarthritis, e.g., terms associated with external matrix organization[45] and skeletal system development,[10,13,14,45] as well as the epithelium-related term "positive regulation of epithelial cell migration" in articular cartilage. This term showed limited overlap with other enriched pathways on the constituent gene level (e.g., extracellular matrix structural constituent: two of 93 annotated, differentially methylated genes are also annotated to "positive regulation of epithelial cell migration," collagen fibril organization: one of 34, integrin-mediated signaling pathway: 11 of 58, cartilage development: seven of 104, chondrocyte differentiation: five of 62) suggesting its distinctness, e.g., to pathways that are linked to the extracellular matrix or cartilage development. This pathway may point to an epithelium-related etiological mechanism.

We used an independent dataset from 17 knee osteoarthritis patients to replicate the epigenetic differences between low-grade and high-grade osteoarthritis cartilage.[11,30] We replicated 7,192 DMSs and 105 DMRs (Tables S2, S3, and S5). The effect sizes of replicated DMSs (Pearson $r = 0.96$, $p < 2.2 \times 10^{-16}$) and DMRs (Pearson $r = 0.95$, p value $< 2.2 \times 10^{-16}$) in the discovery and replication datasets were highly correlated (Figures 3A and 3B). These results point to the robustness of the identified methylation changes.

We further performed EWAS separately on paired low-grade and high-grade cartilage in female ($n = 52$) and

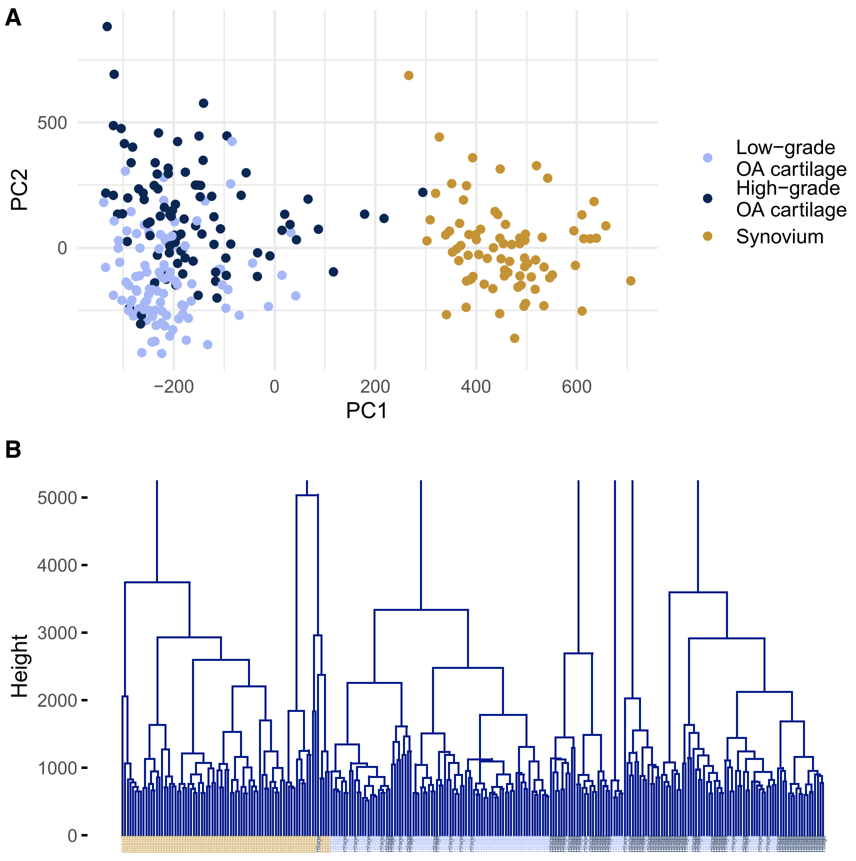

Figure 1. Multivariate analyses of methylation profiles distinguish between different tissues and disease grades
(A) In a principal-component analysis, the first PC separates cartilage from synovium, while the second PC is associated with cartilage grades (with overlapping clusters from low-grade and high-grade osteoarthritis cartilage samples).
(B) Hierarchical clustering shows a separation of global methylation profiles by tissue type. "Height" on the y axis denotes the distance between clusters. OA denotes osteoarthritis.

male (n = 38) osteoarthritis-affected individuals and identified female- (n = 1,338) and male- (n = 3,316) specific DMSs in cartilage, suggesting sex-specific markers (supplemental note S2).

Machine-learning models distinguish cartilage grades with high accuracy

Next, we sought to test whether epigenetic changes in different cartilage grades can be harnessed to develop a model that robustly distinguishes low-grade from high-grade osteoarthritis cartilage. First, we constructed RF-based classifiers in the discovery knee osteoarthritis-affected individual cohort in a repeated 5-fold cross-validation approach. Here, we achieved high prediction accuracies (mean accuracy: 90.69%; standard deviation: 4.08, 95% confidence interval [CI] 89.98–91.41). Furthermore, the resulting receiver operating characteristic (ROC) curve revealed an area under the curve of 0.97 (Figure S3), highlighting the high sensitivity and specificity of these classifiers.

To validate these findings, we trained the final RF-based classifier on our entire patient cohort (subjects and methods) and evaluated its accuracy in an external dataset composed of 17 knee and 14 hip osteoarthritis-affected individuals.[30] In this replication cohort, we achieved an accuracy of 82.35% (95% CI 65.47–93.24) for knee samples, whereas in hip samples the achieved accuracy was lower at 64.29% (95% CI 44.07–81.36). We also observed these differences when using support vector machines (knee: 85.29%, 95% CI 68.94–95.05; hip: 57.14%, 95% CI 37.18–75.54) and gradient-boosting machines (knee: 76.47%, 95% CI 58.83–89.25; hip: 50.00%, 95% CI 30.65–69.35). The lower accuracy achieved in hip samples supports the effect of methylation joint specificity within osteoarthritis.[30,45] GO enrichment analysis of the 300 most important methylation sites in the final RF model did not identify any significant enrichments. Of these 300 methylation sites, 99.3% (n = 298) and 77.7% (n = 233) were among the DMS identified in the discovery and replication analysis, respectively. This suggests that epigenetic markers for cartilage degeneration are prioritized predictors in the classifier. External validation of the classifier was somewhat limited by the small sample size of the replication set, resulting in wide confidence intervals. Hence, validation in larger datasets is further warranted.

This model shows that epigenetic differences can be used to distinguish disease stages in cartilage. Samples of more accessible tissue types (such as blood and synovial fluid) need to be included in the model training and testing to develop a clinically relevant tool.

Genome-wide mQTL maps in osteoarthritis-relevant tissues

We combined DNA methylation data with matching genotype data from the same osteoarthritis-affected individuals to identify genetic variants that are significantly associated with methylation levels of proximal methylation sites (<1 Mb; *cis*-mQTLs). We performed this analysis at the genome-wide scale in low-grade (n = 97) and high-grade (n = 89) osteoarthritis cartilage samples as well as in synovium (n = 78 samples), and identified widespread signal in every tissue (Figure 4, Figure S4, and Table S6). Applying a conservative Bonferroni threshold to correct for the number of tested genetic variant-methylation site pairs per tissue ($p < 1 \times 10^{-11}$), we identified 10,639, 6,785, and 4,493

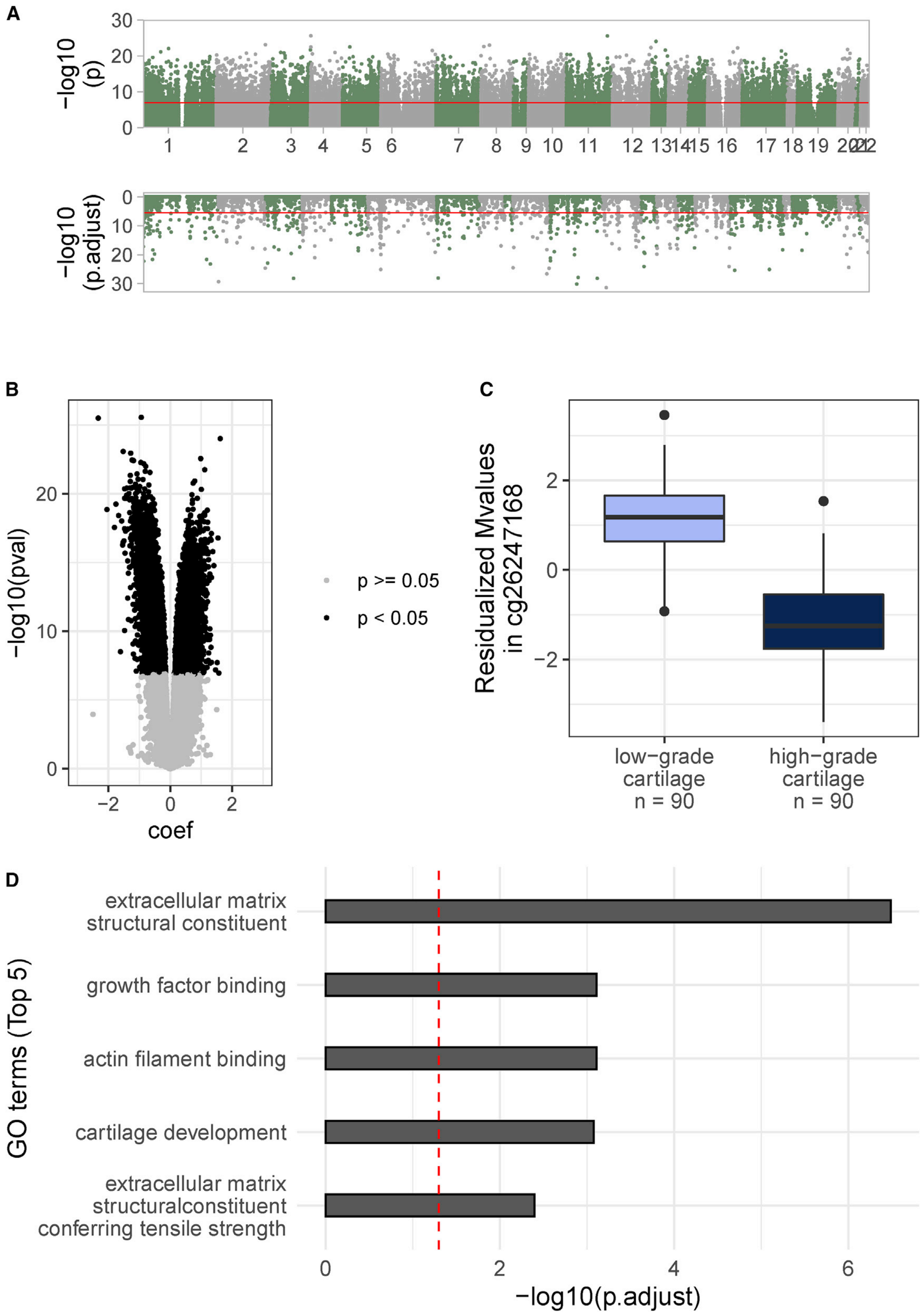

Figure 2. Differential methylation between low-grade and high-grade osteoarthritis cartilage
(A) Genome-wide signals for differential methylation sites (top) and regions (bottom) between low-grade and high-grade osteoarthritis cartilage. Red lines indicate genome-wide significance (top: nominal $p < 1.24 \times 10^{-7}$, bottom: Bonferroni-adjusted $p < 0.05$).
(B) Volcano plot showing hyper- and hypomethylated sites.

(legend continued on next page)

methylation sites significantly associated with at least one mQTL in low-grade osteoarthritis cartilage, high-grade osteoarthritis cartilage, and synovium, respectively, and also included genetic-epigenetic effects in loci of previously reported mQTLs in osteoarthritis-relevant tissue (supplemental note S3). This represents a genome-wide map of mQTLs in osteoarthritis tissues. These data are made publicly available (see data and code availability).

Next, we further characterized the architecture of these mQTL maps. In low-grade and high-grade osteoarthritis cartilage, 66.93% (7,121 of 10,639) and 66.44% (4,508 of 6,785) methylation sites with at least one mQTL were annotated to a gene, respectively (Figures S5 and S6). In both cartilage tissue types, we identified significant (Bonferroni $p < 0.05$) over-representation of intergenic methylation sites or sites within gene bodies and under-representation of methylation sites close to transcription start sites ("TSS200," "TSS1500"), in untranslated regions ("3′ UTR," "5′ UTR") and first exons ("1st exon").

In synovium, 67.44% (n = 3,030 of 4,493) methylation sites with at least one mQTL were annotated to a gene (Figure S7). Here, we found significant (Bonferroni $p < 0.05$) over-representation of intergenic methylation sites and under-representation of methylation sites that are within 200 bp to a transcription start site or in untranslated regions or first exons. These results suggest similar mQTL architectures across osteoarthritis tissues.

Furthermore, we tested whether mQTL effects differ between osteoarthritis-affected individuals of different sexes and identified methylation sites targeted by sex-specific mQTLs (FDR < 0.05) in low-grade (n = 282) and high-grade (n = 337) osteoarthritis cartilage as well as in synovium (n = 874) (Figure S8 and Tables S7, S8, and S9). This suggests sex-specific genetic effects on methylation in osteoarthritis tissues.

Comparing mQTLs in cartilage and synovium with whole blood

Next, we asked whether the mQTL profiles of primary osteoarthritis tissues differ to those of more easily accessible, peripheral tissue samples. We compared the *cis*-mQTL effects of each of the three examined joint tissues with those of whole blood, which is the most commonly examined tissue type for DNA methylation. To maximize the number of identifiable osteoarthritis-tissue-specific effects, we compared *cis*-mQTL effects in joint-tissue to those of a publicly available, large-scale whole blood meta-analysis including 36 studies (27,750 European ancestry participants).[40]

Because a mQTL can be associated with more than one methylation site (and vice versa), we use the term "mQTL-site pair" to indicate the association between a specific mQTL and a specific methylation site. Of the 482,751 mQTL-site pairs in low-grade osteoarthritis cartilage, information of 365,411 were available in whole blood. Of these, 88.6% (n = 323,863) were significant in blood ($p < 10^{-11}$) with a concordant direction of effect. Notably, 9.61% of overlapping mQTL-site pairs (n = 35,117) were both significant and had an opposite direction of effect in blood. Similarly, we compared 219,661 (of 286,558) mQTL-sites pair identified in high-grade osteoarthritis cartilage and found that 90.53% (n = 198,867) of these had a significant ($p < 10^{-11}$) effect in the same direction in blood. Notably, 7.87% (n = 17,297) of present mQTL-site pair had a significant but opposing direction of effect in blood. In synovium, 78.88% (n = 156,931) of 198,958 mQTL-site pair were available in whole blood, of which 96.87% (n = 152,023) had a significant mQTL effect in blood ($p < 10^{-11}$) in the same direction. 2.29% (n = 3,594) of overlapping mQTL-site pairs showed a significant effect in the opposite direction in blood. In summary, we found that the majority of mQTL effects identified in osteoarthritis-related tissues show the same direction in whole blood but also observed effects in opposing directions in all tested joint tissue types. The latter indicates non-negligible differences in the mQTL profile between osteoarthritis-relevant joint tissues and whole blood.

Identification of grade-specific mQTLs in cartilage

We compared mQTL effects between the osteoarthritis joint tissues. Comparing low-grade cartilage with synovium, we found 143,258 mQTL-site pairs to be significant in both. The effects showed high correlation (Pearson r = 0.97, $p < 10^{-16}$) and 33 mQTL-site pairs showed an effect in the opposite direction. The effects of the 122,378 mQTL-site pairs that were significant in both high-grade cartilage and synovium also showed high correlation (Pearson r = 0.97, $p < 2.2 \times 10^{-16}$), and their effect directions were all concordant. In low-grade and high-grade osteoarthritis cartilage, the effect sizes of mQTL-site pairs were highly correlated (comparing 256,036 mQTL-site pairs that were significant in both low-grade and high-grade osteoarthritis cartilage: Pearson r = 0.99, $p < 2.2 \times 10^{-16}$) and showed only concordant effect directions. Overall, our findings point to broadly concordant mQTL effects across osteoarthritis tissues.

We subsequently sought to identify differential mQTLs, i.e., mQTLs that are present in either low-grade or high-grade cartilage but not in both. This can help identify mQTL effects that are potentially "switched on/off" with increasing cartilage degeneration grade, i.e., with disease stage. To this end, we applied a meta-analysis approach,[39] which improves power in identifying differential mQTLs by estimating a posterior probability of >0.9 and <0.1

(C) An example of hypomethylation in high-grade osteoarthritis cartilage at cg26247168 (beta: −2.32, $p = 3.05 \times 10^{-26}$, SE = 0.14). The boxplots represent 25th, 50th, and 75th percentiles, and whiskers extend to 1.5 times the interquartile range.
(D) Most significant Gene Ontology gene annotations enriched in 15,328 DMSs. Red dashed lines indicate the significance threshold (Benjamini-Hochberg-adjusted $p < 0.05$).

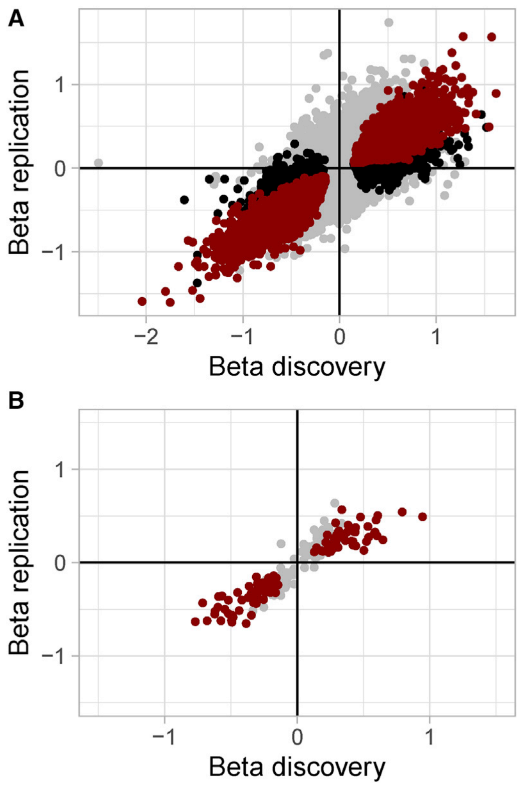

Figure 3. Replication of EWAS results in an independent dataset
(A and B) Effects of (A) methylation sites (n = 346,288) and (B) methylation regions (n = 271) present in both of the discovery and replication datasets. Black dots refer to DMSs/DMRs of the discovery set, red dots to DMSs/DMRs that additionally show an effect at nominal significance (nominal $p < 0.05$) in concordant direction in the replication set.

indicating the presence and absence of a mQTL effect, respectively. In total, we identified 195 genetic variants that show a differential mQTL effect on 18 methylation sites (Table S10). Following clumping, one independent differential mQTL was retained per methylation site (Figure 5). Of the 18 targeted methylation sites, 14 and 4 were mQTLs in low-grade and high-grade osteoarthritis cartilage only, respectively. Genes annotated to these methylation sites are linked to osteoarthritis-relevant terms in cartilage, e.g., they encode a matrix metalloproteinase (*MMEL1*) or are involved in cell adhesion (*CDH23* and *PARVA*).

Assessing the causal role of methylation in osteoarthritis

To identify methylation sites that play a causal role in osteoarthritis progression, we applied two-sample Mendelian randomization (MR) to the methylation sites associated with cartilage degeneration (exposure) and the mQTLs we identified in osteoarthritis-relevant tissues, together with genetic associations from three GWASs: knee osteoarthritis (knee OA), osteoarthritis at any site (all OA), and total knee replacement (TKR). We used the mQTLs as instrumental variables (Figure S9) in the MR analysis.

We identified 6, 8, and 11 significant osteoarthritis trait-methylation site combinations in low-grade and high-grade osteoarthritis cartilage and synovium, respectively. When performing an MR approach to examine causality in the opposite direction, namely the effect of osteoarthritis on methylation (Figure S10), we could not find any evidence for a significant effect for these osteoarthritis trait-methylation site combinations, thus providing further evidence for the causal role of these methylation sites on osteoarthritis (and not vice versa).

In total, we identified 19 methylation sites with a putative causal effect on osteoarthritis (Figure 6 and Table S11). In low-grade osteoarthritis cartilage, we identified six methylation sites with a potential causal effect (Bonferroni correction, $p < 4.95 \times 10^{-6}$). Four of these showed association with hypermethylation, and two showed associations with hypomethylation and osteoarthritis development. Among the annotated genes is *WWP2* (cg26736200 in gene body), a key regulator in chondrocytes (discussion).

In high-grade cartilage, eight methylation sites were causally linked to osteoarthritis (Bonferroni correction, $p < 8.18 \times 10^{-6}$). Of these, five sites showed association of hypermethylation with a protective effect against osteoarthritis development, whereas the other three sites were associated with higher risk. Annotated genes include *COLGALT2* (cg18131582 in gene body), a transferase that catalyzes the transfer of galactose to collagen during collagen synthesis.[46] A previous study suggests that the expression of this gene in cartilage is influenced by an osteoarthritis-risk variant.[47]

In synovium, we identified 11 significant methylation site-trait combinations, involving eight unique methylation sites ($p < 1.07 \times 10^{-5}$). In five of these eight sites, increased methylation levels showed a protective effect against osteoarthritis development, whereas in three sites hypermethylation was associated with higher risk. Annotated genes include *MFHAS1* (cg01784220 in the 1st exon), a gene involved in Toll-like receptor signaling,[48,49] which is thought to be centrally involved in the osteoarthritis-related immune response in synovial joints.[50]

We identified one methylation site (cg26736200) in low-grade osteoarthritis cartilage and two methylation sites (cg17551891 and cg00076555) in high-grade osteoarthritis cartilage that were also identified as potentially causal for osteoarthritis in the synovium. For these three methylation sites, the direction of effect was concordant across tissues. Cg26736200 is annotated to the gene body of *WWP2*. Cg1755189 is located in the gene body of *MAD1L1,* a gene involved in cell-cycle regulation, which may point to cell senescence of chondrocytes in osteoarthritis articular cartilage.[51] Cg00076555 is located in the 3′ UTR of *BSN* (discussion).

Resolution of GWAS signals

We performed a colocalization analysis to determine whether osteoarthritis-linked genetic risk variants exert their effect through the regulation of nearby methylation sites. For all OA, 13 of 33 tested GWAS signals colocalized

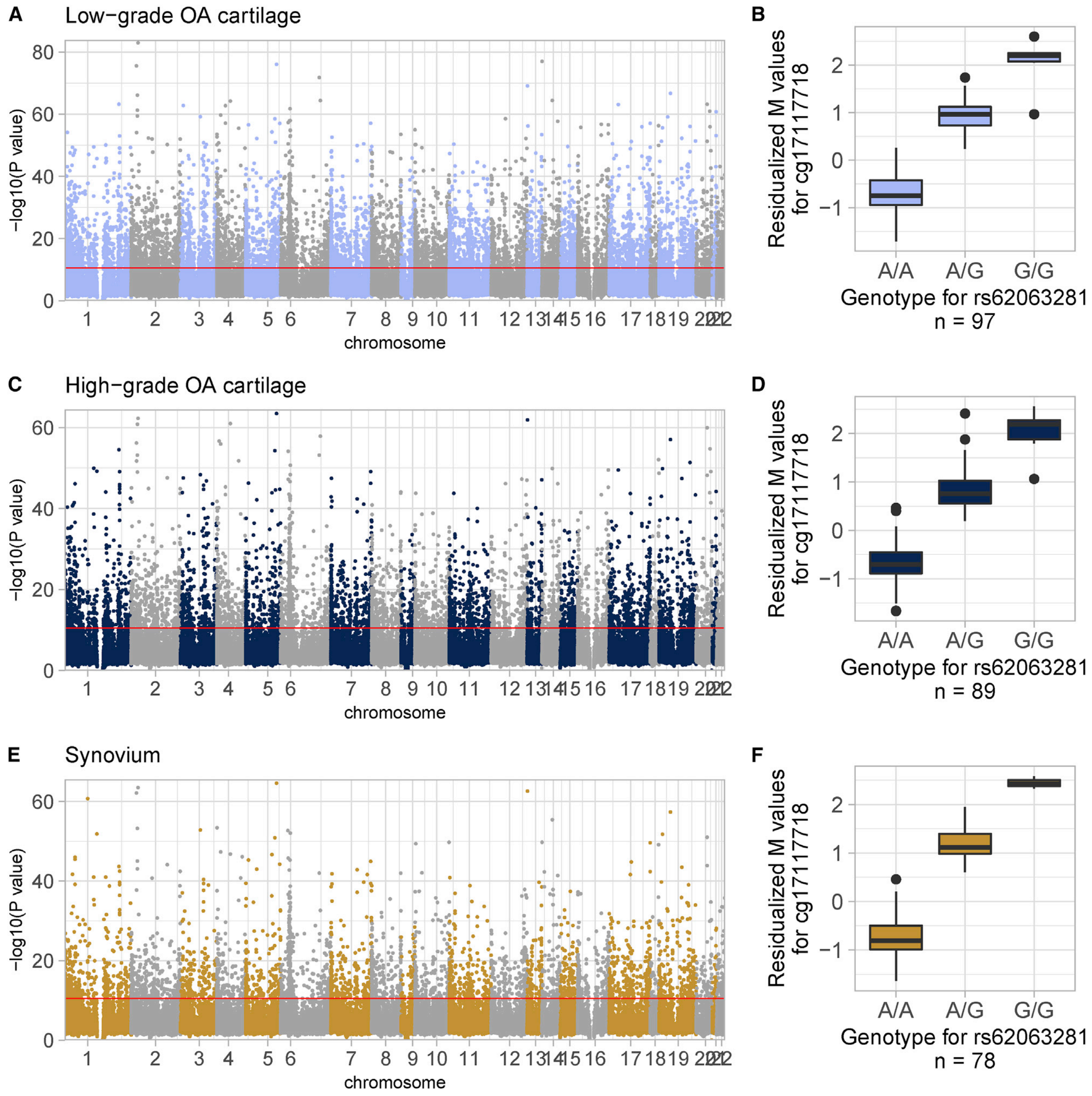

Figure 4. The mQTL landscape in cartilage and synovium
(A–F) Manhattan plots depicting the negative log of the p value of the most significant association per methylation site across all variants within 1 Mb in (A) low-grade osteoarthritis cartilage, (C) high-grade osteoarthritis cartilage, and (E) synovium. Red lines indicate genome-wide significance (Bonferroni correction). The boxplots describe the effect of rs62063281 on methylation site cg17117718 in (B) low-grade osteoarthritis cartilage (beta = 1.65, $p = 1.19 \times 10^{-48}$, SE = 0.06), (D) high-grade osteoarthritis cartilage (beta = 1.60, $p = 3.55 \times 10^{-37}$, SE = 0.07), and (F) synovium (beta = 1.95, $p = 2.39 \times 10^{-44}$, SE = 0.06), as an example. The boxplots represent 25th, 50th, and 75th percentiles, and whiskers extend to 1.5 times the interquartile range.

with mQTLs (ten in low-grade osteoarthritis cartilage, seven in high-grade osteoarthritis cartilage, and six in synovium; example in Figure 7B). For knee OA, six of 12 tested GWAS signals colocalized with mQTL signals (five in low-grade osteoarthritis cartilage, four in high-grade osteoarthritis cartilage, and four in synovium; example in Figure 7A). For TKR, one of five tested GWAS signals colocalized with mQTL signals (in low-grade osteoarthritis cartilage). Overall, osteoarthritis-related GWAS signals colocalized with mQTL signals of 32 unique methylation sites in low-grade osteoarthritis cartilage, 29 in high-grade osteoarthritis cartilage, and 17 in synovium. In total, we colocalized mQTL signals of 56 unique methylation sites with osteoarthritis-risk variants across the three affected individual tissues (Table S12).

By comparing the findings from colocalization and causal inference analysis (in the previous section), we identified two methylation sites in low-grade osteoarthritis

A

variant	Gene	Msite	Gene (Msite)	Beta (L−G)	Beta Standard error (L−G)	Posterior Prob (L−G)	Beta (H−G)	Beta Standard error (H−G)	Posterior Prob (H−G)	Tissue with QTL effect
rs62175812	*CERS6*	cg04746649	*LASS6*	−0.927	0.157	1.000	−0.082	0.151	0.004	L−G
rs10055514	*TMED7*	cg05930586		0.893	0.139	1.000	−0.012	0.130	0.000	L−G
rs793442	*FILIP1L*<br>*CMSS1*	cg06077670	*C3orf26*<br>*FILIP1L*<br>*MIR548G*	0.886	0.140	1.000	−0.086	0.130	0.000	L−G
rs13148169	*intergenic*	cg06759629		0.998	0.159	1.000	0.187	0.154	0.090	L−G
rs2072184	*TWISTNB*<br>*MIR3146*	cg07541023	*TWISTNB*	0.924	0.150	1.000	0.017	0.145	0.001	L−G
rs2956078	*APIP*	cg11058730	*PDHX*<br>*APIP*	0.845	0.132	1.000	0.161	0.126	0.021	L−G
rs56918106	*SEMA3D*	cg11878016		1.016	0.151	1.000	0.248	0.137	0.033	L−G
rs4789346	*MXRA7*	cg13533061	*JMJD6*	−0.872	0.133	1.000	−0.186	0.120	0.052	L−G
rs10760430	*MVB12B*<br>*NRON*	cg13960660	*FAM125B*	1.053	0.170	1.000	0.181	0.158	0.089	L−G
rs3823467	*LINC00574*<br>*LINC00242*	cg16413842	*C6orf122*<br>*C6orf208*	1.136	0.186	1.000	0.172	0.182	0.062	L−G
rs4988988	*RP11−219G17.4*<br>*THA1P*	cg16810279		0.987	0.158	1.000	0.224	0.151	0.095	L−G
rs4708476	*SMOC2*	cg18005896	*SMOC2*	−1.006	0.133	1.000	−0.261	0.121	0.057	L−G
rs79031158	*TTC34*	cg21389723	*MMEL1*	0.763	0.140	1.000	0.081	0.138	0.016	L−G
rs2104958	*intergenic*	cg25193276		−0.990	0.154	1.000	−0.216	0.144	0.080	L−G
rs113856858	*SGPL1*	cg09598552	*CDH23*	0.200	0.280	0.003	−1.978	0.328	1.000	H−G
rs7481217	*TEAD1*	cg18778433	*PARVA*	0.211	0.242	0.044	1.812	0.298	1.000	H−G
rs2095124	*RASSF5*<br>*RP11−534L20.5*	cg19452316	*RASSF5*	0.279	0.167	0.074	1.257	0.194	1.000	H−G
rs73088790	*WDR82*	cg23168339	*WDR82*	−0.007	0.228	0.001	−1.868	0.286	1.000	H−G

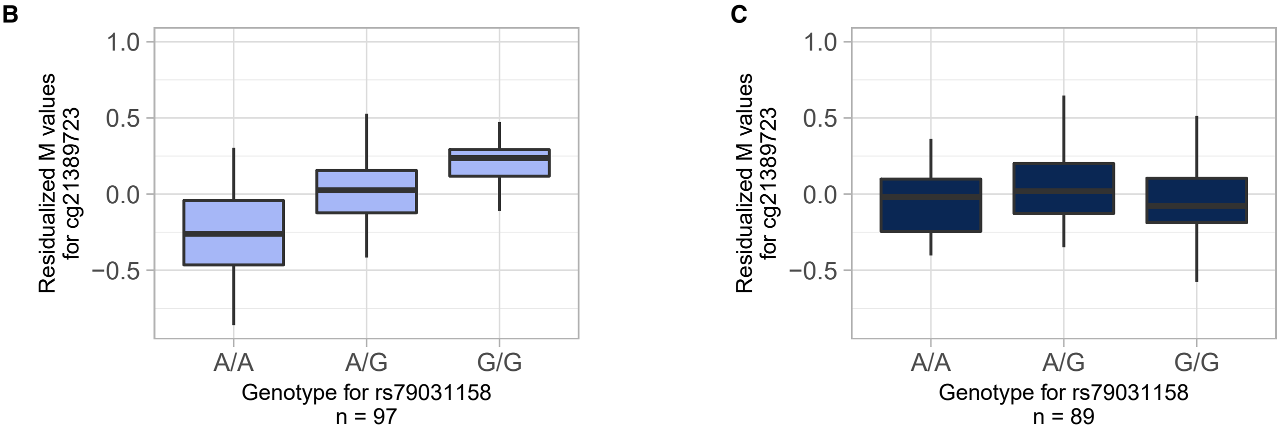

Figure 5. Differential mQTLs

(A–C) Each row refers to a variant with a differential mQTL effect. The table reports the genetic variant and the targeted methylation site as well as annotated genes, effect sizes with corresponding standard errors, and posterior probabilities (>0.9 indicate an effect, <0.1 indicate no effect) for the effects in low-grade or high-grade osteoarthritis cartilage. The reported effects were estimated by a meta-analysis approach (subjects and methods). Boxplots (B) and (C) exemplify a differential mQTL: rs79031158 is associated with methylation of cg21389723 in low-grade (B) but not in high-grade osteoarthritis cartilage (C). The boxplots represent 25th, 50th, and 75th percentiles, and whiskers extend to 1.5 times the interquartile range. Msite, methylation site; L-G, low-grade osteoarthritis cartilage; H-G, high-grade osteoarthritis cartilage; Posterior Prob, posterior probability.

cartilage (cg17125990 and cg26736200) and one methylation site in synovium (cg26736200) across both approaches, providing further evidence that these methylation sites play a causal role in osteoarthritis in the respective joint tissue.

Next, we combined these findings with results from eQTL data[3] generated in the same patient cohort. When osteoarthritis GWAS signals colocalized with mQTL data, we tested whether the GWAS signal index variant exerted an effect on the expression levels of any gene close to the relevant methylation site. We found such an eQTL effect below nominal significance levels for five genes in low-grade osteoarthritis cartilage (*ALDH1A2*, *CHMP1A*, *FAM53A*, *RPP25*, and *TGFA*), two genes in high-grade

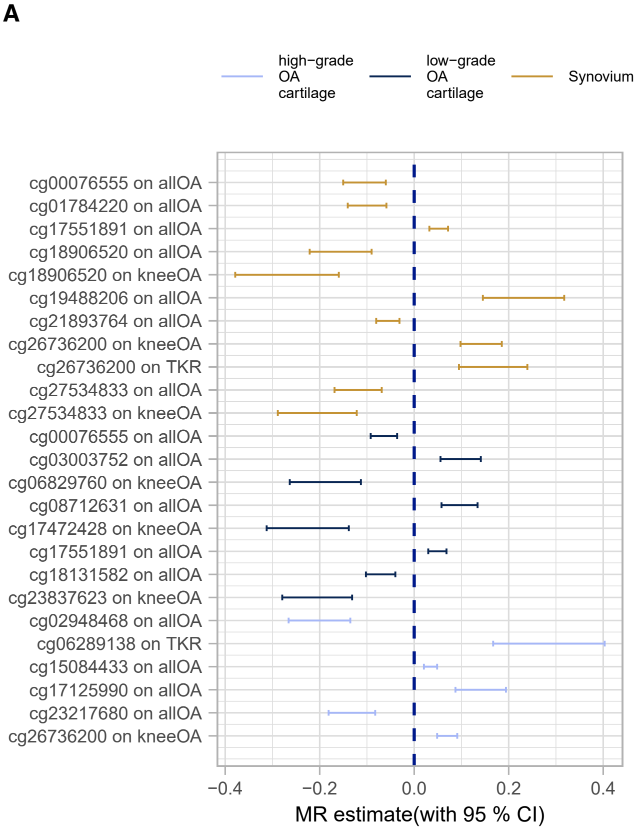

B

Exposure	Tissue	Outcome	IV	Exposure Gene
cg00076555	Synov	allOA	rs34196454	*BSN*
cg01784220	Synov	allOA	rs13281377	*MFHAS1*
cg17551891	Synov	allOA	rs62442906	*MAD1L1*
cg18906520	Synov	allOA	rs11613937	*OGFOD2*
cg18906520	Synov	kneeOA	rs11613937	*OGFOD2*
cg19488206	Synov	allOA	rs3771584	*STK25*
cg21893764	Synov	allOA	rs2076311	*COL11A2*
cg26736200	Synov	kneeOA	rs11647767	*WWP2*
cg26736200	Synov	TKR	rs11647767	*WWP2*
cg27534833	Synov	allOA	rs11613937	
cg27534833	Synov	kneeOA	rs11613937	
cg00076555	H-G	allOA	rs3197999	*BSN*
cg03003752	H-G	allOA	rs1050847	
cg06829760	H-G	kneeOA	rs1024923	*FAM49A*
cg08712631	H-G	allOA	rs4721170	*MAD1L1*
cg17472428	H-G	kneeOA	rs11867782	
cg17551891	H-G	allOA	rs35327420	*MAD1L1*
cg18131582	H-G	allOA	rs10797923	*GLT25D2*
cg23837623	H-G	kneeOA	rs2641436	*SLC43A2*
cg02948468	L-G	allOA	rs892085	*ILF3*
cg06289138	L-G	TKR	rs9469323	*C6orf227*
cg15084433	L-G	allOA	rs7803925||rs3808082	*SND1*
cg17125990	L-G	allOA	rs56338926	
cg23217680	L-G	allOA	rs435581	
cg26736200	L-G	kneeOA	rs61611907	*WWP2*

Figure 6. Overview of putative causal effects of methylation on osteoarthritis-related traits
(A and B) Forest plot (A) describing the putative causal effect (with 95% confidence interval) of increasing methylation levels in the respective sites on osteoarthritis-related traits. Only significant exposure-outcome associations exceeding tissue-specific Bonferroni thresholds are reported (low-grade osteoarthritis cartilage: $p < 4.95 \times 10^{-6}$, high-grade osteoarthritis cartilage: $p < 8.18 \times 10^{-6}$, synovium: $p < 1.05 \times 10^{-5}$). The table (B) reports the instrumental variable(s) (IV[s]) and annotated genes. We applied the Wald-ratio test in cases of one IV; otherwise the inverse-variance-weighted method was applied.

osteoarthritis cartilage (*FAM53A* and *LTBP1*), and one gene in synovium (*CRADD*) (Figure 7C). In total, we identified seven genes linked to an osteoarthritis-risk locus. Given their link to osteoarthritis-risk variants across two molecular layers, these genes are high-confidence effector genes at these osteoarthritis GWAS loci in the respective tissue.

We compared these results with findings from a recent differential expression analysis.[3] Two high-confidence effector genes were shown to be differentially expressed in high-grade compared to low-grade osteoarthritis cartilage (in high-grade osteoarthritis cartilage, *ALDH1A2* is overexpressed with FDR = 0.0017 and logFC = 0.38 and *CRADD* is underexpressed with FDR = 0.00067 and logFC = −0.24), thus providing additional supportive evidence for a role in osteoarthritis.

Next, we tested whether high-confidence effector genes correlate with nearby methylation sites, which in turn putatively mediate the effect of osteoarthritis-risk variants. Using expression and methylation data of the same osteoarthritis-affected individuals in the same tissue, we identified such expression quantitative trait methylation (eQTM) effects at nominal significance ($p < 0.05$) for three genes (*ALDH1A2*, *FAM53A*, and *RPP25*) in low-grade cartilage and one gene (*LTBP1*) in high-grade cartilage (Figure 7C). To assess whether these observed associations are solely correlations, or whether methylation levels do have a causal effect on gene expression (by mediating the genetic effect on gene expression), we performed one-sample MR (supplemental subjects and methods). We found evidence (MR $p < 0.05$) for a causal effect of methylation on gene expression levels for two genes (*ALDH1A2* and *RPP25*) in low-grade osteoarthritis cartilage and one gene (*LTBP1*) in high-grade osteoarthritis cartilage (Table S13). These findings suggest that methylation mediates the effect of genetic variants on expression for these high-confidence effector genes.

Comparing colocalization of osteoarthritis loci in joint and whole blood mQTL data

To investigate the joint tissue specificity of colocalizing joint mQTL and osteoarthritis GWAS data, we asked whether these results could also be identified in whole blood (supplemental note S4). This would allow us to better understand whether the regulatory effects of osteoarthritis-risk loci mediated by proximal methylation sites are exclusive to disease-affected joint tissues or also observed in peripheral tissues. We tested whether the pairs of risk variant-methylation sites that colocalize in at least

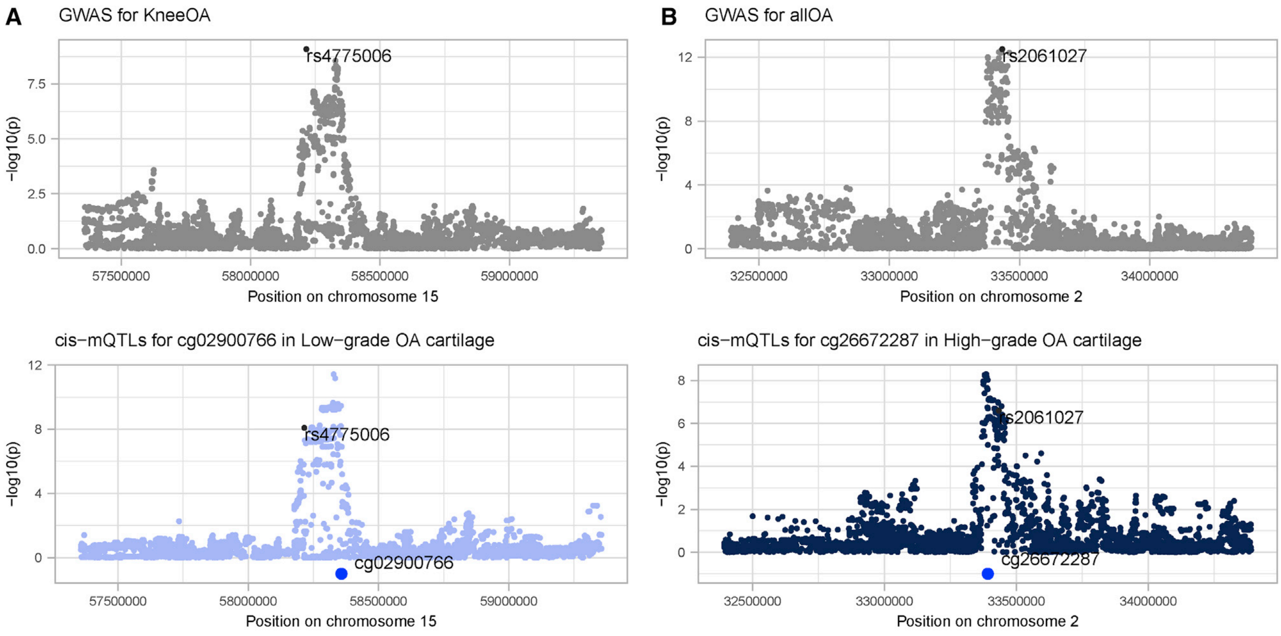

C

GWAS Trait	GWAS lead variant	RA	mQTL Tissue	Msite	Msite Location (Gene)	RA effect on meth	Coloc PP	Gene	RA effect on exp	p−value (RA effect on exp)	Effect Meth on exp	p−value (Effect Meth on exp)
allOA	rs798726	C	H−G	cg15763121	3'UTR	−	0.81	FAM53A	−	0.002		
allOA	rs2061027	A	H−G	cg26672287	Body	+	0.87	LTBP1	−	0.008	−	0.046
allOA	rs798726	C	L−G	cg00626702	Body	−	0.82	FAM53A	−	3e−05		
allOA	rs35206230	T	L−G	cg05627522	1776_tss	+	0.94	RPP25	−	0.022	−	0.004
allOA	rs798726	C	L−G	cg07428655	3'UTR	−	0.91	FAM53A	−	3e−05		
allOA	rs798726	C	L−G	cg07929082	5'UTR	+	0.94	FAM53A	−	3e−05		
allOA	rs1126464	G	L−G	cg08723459	3'UTR	+	0.88	CHMP1A	−	0.007		
allOA	rs798726	C	L−G	cg15763121	3'UTR	−	0.91	FAM53A	−	3e−05		
allOA	rs35206230	T	L−G	cg18612461	1928_tss	+	0.94	RPP25	−	0.022	−	0.005
allOA	rs798726	C	L−G	cg21231739	3'UTR	−	0.91	FAM53A	−	3e−05	+	0.037
allOA	rs3771501	A	L−G	cg27223728	3415_tss	−	0.96	TGFA	−	0.002		
kneeOA	rs4775006	A	L−G	cg02900766	Body	−	0.99	ALDH1A2	+	0.003	−	0.023
kneeOA	rs4775006	A	L−G	cg12382153	Body	−	0.91	ALDH1A2	+	0.003		
TKR	rs34958088	G	L−G	cg02900766	Body	−	0.99	ALDH1A2	+	7e−04	−	0.023
TKR	rs34958088	G	L−G	cg12382153	Body	−	0.93	ALDH1A2	+	7e−04		
allOA	rs2171126	T	Synov	cg19571390	Body	+	0.86	CRADD	+	0.003		

Figure 7. Colocalization reveals overlapping signals in GWAS and mQTL data
(A–C) (A) and (B) exemplify colocalization events. In (A), we colocalized signals of *cis*-mQTL for the methylation site cg02900766 (A, bottom) with the GWAS for knee OA in the same genomic region (A, top). Here, we observed a posterior probability (PP) for a shared causal variant of 98.6%. Similarly, (B) visualizes the colocalization (PP = 86.5%) of *cis*-mQTL signals targeting cg26672287 in high-grade osteoarthritis cartilage (bottom) with GWAS signals for all OA (top). The highlighted variant (black) refers to the GWAS index variant in the respective genetic locus. (C) outlines osteoarthritis-linked genetic variants that colocalize with a methylation site and additionally show an eQTL effect at nominal significance (nominal $p < 0.05$) on the gene annotated to the respective methylation site in the same tissue. For four genes, we also identified an association (at nominal significance) with methylation sites for which *cis*-mQTLs, in turn, colocalize with a GWAS signal. RA, risk allele; Msite, methylation site; Coloc PP, posterior probability for colocalization; exp, gene expression; meth, methylation; _tss (in column Msite Location), methylation sites that are close to a transcript start site of the respective gene. The preceding number refers to the distance in bp.

one joint tissue also colocalize in a large whole blood mQTL meta-analysis.[40]

Considering all OA-risk variants, we found 15 risk variant-methylation site pairs for which we estimated co-localizing GWAS signals and mQTL in at least one joint tissue (ten and five pairs in low-grade and high-grade osteoarthritis cartilage, respectively), but not in whole blood. These pairs involved eight all OA-risk variants in total (seven and three all OA signals in low-grade and high-grade osteoarthritis cartilage, respectively).

For the knee OA-risk variants, we identified five risk variant-methylation site pairs with colocalizing GWAS

signals and mQTL in at least one joint tissue (two, two, and one pairs in low-grade and high-grade osteoarthritis cartilage and synovium, respectively) but not in whole blood. These pairs involved two unique knee OA-risk variants (rs9277552 in low-grade and high-grade osteoarthritis cartilage and rs56116847 in synovium). For the TKR-risk variants, we did not find evidence for joint-tissue-specific colocalizations.

Discussion

Osteoarthritis is a common disease with a complex polygenic architecture. In this study, we analyzed the genome-wide methylation profile of low-grade osteoarthritis cartilage, high-grade osteoarthritis cartilage, and synovium at unprecedented scale and depth. We identified and biologically characterized DNA methylation markers of osteoarthritis grade and generated genome-wide maps of mQTLs in three understudied osteoarthritis-relevant tissues, which we used to identify mechanistically relevant genes.

Our data revealed global differences in the methylation profile between tissue types (cartilage versus synovium) and cartilage degeneration states (low-grade versus high-grade osteoarthritis cartilage), with robust evidence for replication in an independent dataset despite lower power due to smaller replication sample size. This study represents a large EWAS for knee cartilage degeneration, increasing the number of studied knee osteoarthritis-affected individuals by almost 6-fold, thus providing substantially higher power compared to previous studies. Together, our findings underline the cell type and osteoarthritis-grade specificity of DNA methylation in primary tissues, thus highlighting the importance of expanding molecular studies of complex diseases to multiple relevant tissues and cell types.

Indeed, comparison of our findings with methylation data available in peripheral blood further underlined the value of analyzing primary tissues. Observed differences included mQTLs with opposite directions of effect and evidence for colocalization in joint tissue, but not in whole blood, for genetic variants linked with osteoarthritis. These findings suggest that at least a subset of the regulatory effects conferred by osteoarthritis-linked variants through proximal methylation sites are specific to osteoarthritis-affected tissue. More generally, they emphasize the value of investigating disease-relevant tissues rather than solely relying on molecular data in peripheral tissue types.

Characterization of knee cartilage degeneration methylation markers revealed the involvement of biological processes such as external matrix organization, skeletal system development, and signaling pathways, which mirror the broad spectrum of physiological mechanisms observed during cartilage degeneration.[52] Our results indicate that the aetiology of osteoarthritis is partly regulated through aberrant DNA methylation. Notably, we report an enrichment of the epithelium-related term "positive regulation of epithelial cell migration." Given the role of epithelial cells in lining body cavities, in particular blood vessels, this finding may suggest that methylation is involved in the pathogenic release of pro-angiogenic factors. Our findings provide evidence that epithelium-linked mechanisms are relevant in osteoarthritic changes of the articular cartilage in affected joints.

Our study presents a genome-wide map of mQTLs in low-grade and high-grade osteoarthritis cartilage as well as in the synovium of osteoarthritis-affected knees. We identified 18 differential mQTLs between low-grade and high-grade osteoarthritis cartilage. This finding suggests distinct regulatory effects of genetic variants on methylation early and late in the cartilage degeneration process, thus proposing changing genetic influences on epigenetic profiles during osteoarthritis progression.

We identified methylation sites that play a putative causal role in osteoarthritis, for example for the *WWP2*, *BSN*, and *MFHAS1* genes. *WWP2* codes for WW domain-containing E3 ubiquitin protein ligase 2, which is involved in protein ubiquitination. *WWP2* is the host gene of micro RNA 140, a key regulator in chondrocytes, which is targeted by methylation in that region. *Wwp2* has previously been implicated in cartilage homeostasis through regulation of *Adamts5*, a gene encoding an aggrecanase. In addition, *WWP2* demonstrates decreased expression levels in osteoarthritis-affected articular cartilage derived from samples of affected individuals.[53] Our findings indicate that methylation may be driving this aberrant mechanism. Previously, a study identified an mQTL that targets methylation sites in *WWP2*.[17] Another study found *WWP2* expression to be significantly associated with proximal genetic variants and methylation levels of close methylation sites.[11] Together, these results support a role for genetically determined methylation for *WWP2* regulation in osteoarthritis. *BSN* encodes a protein involved in neurotransmission. In the active zone of the synapse, BSN is part of the scaffold of the presynaptic skeleton complex, a structure that assists in the vesicle fusion of synaptic vesicles and presynaptic membranes.[54] This finding may point to innervation in cartilage and synovium during osteoarthritis. *MFHAS1* plays a role in controlling Toll-like receptors TLR2 and TLR4,[48,49] which in turn promote inflammation of the synovium. Toll-like receptors are exposed by cells in the synovium. They bind released matrix molecules of degraded cartilage, which leads to the formation of chemokines and cytokines, in turn leading to the inflammatory cell infiltration of the synovium.[55]

We found evidence for 56 methylation sites mediating the effects of proximal osteoarthritis-linked genetic variants in osteoarthritis-relevant tissue. For seven genes (*ALDH1A2*, *CHMP1A*, *CRADD, FAM53A*, *LTBP1*, *RPP25*, and *TGFA*), we found evidence that GWAS signals for osteoarthritis colocalize with mQTLs in these genes and are additionally associated with gene expression levels in the same tissue. Four of these genes (*ALDH1A2*, *FAM53A*, *LTBP1*, and *RPP25*) showed an association between

expression and methylation. Together, our results provide support for a regulatory role of the associated genetic variants across two molecular layers, and altered gene expression is modulated through genetically determined DNA methylation levels.

CRADD is an adapter protein involved in apoptosis and plays a role in the formation of the PIDDosome-complex, which in turn triggers *CASP2*.[56] A role for dysregulated apoptosis in osteoarthritis synovial tissue has been previously suggested.[57] Our findings indicate that the apoptosis-contributing factor *CRADD* is regulated through DNA methylation in synovium. *ALDH1A2* codes for an enzyme that catalyzes the reaction from retinaldehyde to retinoic acid, an activated form of vitamin A. Retinoic acid has been linked to the degeneration of collagen in bone[58] and is further used as an agent to induce matrix degeneration in cartilage samples.[59] LTBP1 plays an essential role in the regulation of transforming growth factor (TGF) betas, a cytokine class that has been involved in extracellular matrix synthesis and maintenance, but also moderates the effects of inflammation and controls hypertrophy of chondrocytes.[60] TGF betas are produced by chondrocytes in their inactivated form. LTBP1 binds these inactive TGF-betas to the extracellular matrix in cartilage.[61]

Together, causal inference and colocalization analyses point to methylation sites that putatively contribute to osteoarthritis in synovium as well as in early (low-grade osteoarthritis cartilage) and late disease stages (high-grade cartilage).

In summary, our results highlight the cell type as well as disease-grade specificity of the methylome in osteoarthritis-relevant tissue. We identify evidence for the involvement of epithelium-related pathways and identify likely effector genes for hitherto unresolved osteoarthritis GWAS signals. In several cases, we are able to decipher the molecular mechanism underpinning these associations and demonstrate an important role for DNA methylation in the aetiopathogenesis of this debilitating disease.

Data and code availability

Methylation QTL, Mendelian randomization, and differential methylation results can be obtained online (hmgubox and the Downloads page of the Musculoskeletal Knowledge Portal, see web resources). All software used in this study is available from free repositories or manufacturers as referenced in the web Resources and supplemental subjects and methods.

Supplemental information

Supplemental information can be found online at https://doi.org/10.1016/j.ajhg.2022.05.010.

Acknowledgments

We are grateful to Georgia Katsoula, Arthur Gilly, Andrei Barysenka, and Iris Fischer for helpful contributions. This work was funded by the Wellcome Trust (206194).

Author contributions

Study design: E.Z., J.M.W.; Clinical collection: J.M.W.; Data Analysis: P.K., K.H., L.S., J.S.; Interpretation of results: P.K., E.Z., M.S., J.M.W., C.L.R.; Replication data: R.C.A., I.M.; Manuscript drafting: P.K., E.Z.; Manuscript reviewing and editing: all authors.

Declaration of interests

The authors declare no competing interests.

Received: February 2, 2021
Accepted: May 11, 2022
Published: June 8, 2022

Web resources

An epigenome-wide view of osteoarthritis in primary tissues, https://hmgubox.helmholtz-muenchen.de/d/a23fce319fd844d4b293/

coloc.fast function, https://github.com/tobyjohnson/gtx/blob/526120435bb3e29c39fc71604eee03a371ec3753/R/coloc.R

Ensembl Variant Effect Predictor, http://grch37.ensembl.org/Homo_sapiens/Tools/VEP/

Gene Expression Omnibus database, https://www.ncbi.nlm.nih.gov/geo/ (GEO: GSE63106)

Genetics of DNA Methylation Consortium, http://mqtldb.godmc.org.uk/

GWAS catalog, https://www.ebi.ac.uk/gwas

MetaTissue, http://genetics.cs.ucla.edu/metatissue/

Musculoskeletal Knowledge Portal, http://mskkp.org

References

1. Vos, T., Flaxman, A.D., Naghavi, M., Lozano, R., Michaud, C., Ezzati, M., Shibuya, K., Salomon, J.A., Abdalla, S., Aboyans, V., et al. (2012). Years lived with disability (YLDs) for 1160 sequelae of 289 diseases and injuries 1990–2010: a systematic analysis for the Global Burden of Disease Study 2010. Lancet *380*, 2163–2196. https://doi.org/10.1016/S0140-6736(12)61729-2.
2. Boer, C.G., Hatzikotoulas, K., Southam, L., Stefánsdóttir, L., Zhang, Y., Coutinho de Almeida, R., Wu, T.T., Zheng, J., Hartley, A., Teder-Laving, M., et al. (2021). Deciphering osteoarthritis genetics across 826,690 individuals from 9 populations. Cell *184*, 4784–4818.e17. https://doi.org/10.1016/j.cell.2021.07.038.
3. Steinberg, J., Southam, L., Roumeliotis, T.I., Clark, M.J., Jayasuriya, R.L., Swift, D., Shah, K.M., Butterfield, N.C., Brooks, R.A., McCaskie, A.W., et al. (2021). A molecular quantitative trait locus map for osteoarthritis. Nat. Commun. *12*, 1309. https://doi.org/10.1038/s41467-021-21593-7.
4. Brena, R.M., Huang, T.H.-M., and Plass, C. (2006). Toward a human epigenome. Nat. Genet. *38*, 1359–1360. https://doi.org/10.1038/ng1206-1359.
5. van Dongen, J., Nivard, M.G., Willemsen, G., Hottenga, J.-J., Helmer, Q., Dolan, C.V., Ehli, E.A., Davies, G.E., van Iterson, M., Breeze, C.E., et al. (2016). Genetic and environmental influences interact with age and sex in shaping the human methylome. Nat. Commun. *7*, 11115. https://doi.org/10.1038/ncomms11115.

6. GTEx Consortium (2020). The GTEx Consortium atlas of genetic regulatory effects across human tissues. Science *369*, 1318–1330. https://doi.org/10.1126/science.aaz1776.
7. Dunham, I., Kundaje, A., Aldred, S.F., Collins, P.J., Davis, C.A., Doyle, F., Epstein, C.B., Frietze, S., Harrow, J., Kaul, R., et al. (2012). An integrated encyclopedia of DNA elements in the human genome. Nature *489*, 57–74. https://doi.org/10.1038/nature11247.
8. Kundaje, A., Meuleman, W., Ernst, J., Bilenky, M., Yen, A., Heravi-Moussavi, A., Kheradpour, P., Zhang, Z., Wang, J., Ziller, M.J., et al. (2015). Integrative analysis of 111 reference human epigenomes. Nature *518*, 317–330. https://doi.org/10.1038/nature14248.
9. Stunnenberg, H.G., and Hirst, M. (2016). The international human epigenome Consortium: a blueprint for scientific collaboration and discovery. Cell *167*, 1897. https://doi.org/10.1016/j.cell.2016.12.002.
10. Bonin, C.A., Lewallen, E.A., Baheti, S., Bradley, E.W., Stuart, M.J., Berry, D.J., van Wijnen, A.J., and Westendorf, J.J. (2016). Identification of differentially methylated regions in new genes associated with knee osteoarthritis. Gene *576*, 312–318. https://doi.org/10.1016/j.gene.2015.10.037.
11. den Hollander, W., Ramos, Y.F.M., Bomer, N., Elzinga, S., van der Breggen, R., Lakenberg, N., de Dijcker, W.J., Suchiman, H.E.D., Duijnisveld, B.J., Houwing-Duistermaat, J.J., et al. (2015). Transcriptional associations of osteoarthritis-mediated loss of epigenetic control in articular cartilage. Arthritis Rheumatol. *67*, 2108–2116. https://doi.org/10.1002/art.39162.
12. Moazedi-Fuerst, F.C., Hofner, M., Gruber, G., Weinhaeusel, A., Stradner, M.H., Angerer, H., Peischler, D., Lohberger, B., Glehr, M., Leithner, A., et al. (2014). Epigenetic differences in human cartilage between mild and severe OA. J. Orthop. Res. *32*, 1636–1645. https://doi.org/10.1002/jor.22722.
13. Steinberg, J., Ritchie, G.R.S., Roumeliotis, T.I., Jayasuriya, R.L., Clark, M.J., Brooks, R.A., Binch, A.L.A., Shah, K.M., Coyle, R., Pardo, M., et al. (2017). Integrative epigenomics, transcriptomics and proteomics of patient chondrocytes reveal genes and pathways involved in osteoarthritis. Sci. Rep. *7*, 8935. https://doi.org/10.1038/s41598-017-09335-6.
14. Zhang, Y., Fukui, N., Yahata, M., Katsuragawa, Y., Tashiro, T., Ikegawa, S., and Michael Lee, M.T. (2016). Genome-wide DNA methylation profile implicates potential cartilage regeneration at the late stage of knee osteoarthritis. Osteoarthritis Cartilage *24*, 835–843. https://doi.org/10.1016/j.joca.2015.12.013.
15. Bomer, N., den Hollander, W., Ramos, Y.F.M., Bos, S.D., van der Breggen, R., Lakenberg, N., Pepers, B.A., van Eeden, A.E., Darvishan, A., Tobi, E.W., et al. (2015). Underlying molecular mechanisms of DIO2 susceptibility in symptomatic osteoarthritis. Ann. Rheum. Dis. *74*, 1571–1579. https://doi.org/10.1136/annrheumdis-2013-204739.
16. Rice, S.J., Aubourg, G., Sorial, A.K., Almarza, D., Tselepi, M., Deehan, D.J., Reynard, L.N., and Loughlin, J. (2018). Identification of a novel, methylation-dependent, RUNX2 regulatory region associated with osteoarthritis risk. Hum. Mol. Genet. *27*, 3464–3474. https://doi.org/10.1093/hmg/ddy257.
17. Rice, S.J., Cheung, K., Reynard, L.N., and Loughlin, J. (2019a). Discovery and analysis of methylation quantitative trait loci (mQTLs) mapping to novel osteoarthritis genetic risk signals. Osteoarthritis Cartilage *27*, 1545–1556. https://doi.org/10.1016/j.joca.2019.05.017.
18. Rice, S.J., Tselepi, M., Sorial, A.K., Aubourg, G., Shepherd, C., Almarza, D., Skelton, A.J., Pangou, I., Deehan, D., Reynard, L.N., and Loughlin, J. (2019b). Prioritization of PLEC and GRINA as Osteoarthritis Risk Genes Through the Identification and Characterization of Novel Methylation Quantitative Trait Loci. Arthritis Rheumatol. *71*, 1285–1296. https://doi.org/10.1002/art.40849.
19. Rushton, M.D., Reynard, L.N., Young, D.A., Shepherd, C., Aubourg, G., Gee, F., Darlay, R., Deehan, D., Cordell, H.J., and Loughlin, J. (2015). Methylation quantitative trait locus analysis of osteoarthritis links epigenetics with genetic risk. Hum. Mol. Genet. *24*, 7432–7444. https://doi.org/10.1093/hmg/ddv433.
20. Mathiessen, A., and Conaghan, P.G. (2017). Synovitis in osteoarthritis: current understanding with therapeutic implications. Arthritis Res. Ther. *19*, 18. https://doi.org/10.1186/s13075-017-1229-9.
21. Parker, E., Hofer, I.M.J., Rice, S.J., Earl, L., Anjum, S.A., Deehan, D.J., and Loughlin, J. (2021). Multi-tissue epigenetic and gene expression analysis combined with epigenome modulation identifies RWDD2B as a target of osteoarthritis susceptibility. Arthritis Rheumatol. *73*, 100–109. https://doi.org/10.1002/art.41473.
22. Sorial, A.K., Hofer, I.M.J., Tselepi, M., Cheung, K., Parker, E., Deehan, D.J., Rice, S.J., and Loughlin, J. (2020). Multi-tissue epigenetic analysis of the osteoarthritis susceptibility locus mapping to the plectin gene PLEC. Osteoarthritis Cartilage *28*, 1448–1458. https://doi.org/10.1016/j.joca.2020.06.001.
23. Aryee, M.J., Jaffe, A.E., Corrada-Bravo, H., Ladd-Acosta, C., Feinberg, A.P., Hansen, K.D., and Irizarry, R.A. (2014). Minfi: a flexible and comprehensive Bioconductor package for the analysis of Infinium DNA methylation microarrays. Bioinformatics *30*, 1363–1369. https://doi.org/10.1093/bioinformatics/btu049.
24. Fortin, J.-P., Triche, T.J., and Hansen, K.D. (2017). Preprocessing, normalization and integration of the Illumina HumanMethylationEPIC array with minfi. Bioinformatics *33*, 558–560. https://doi.org/10.1093/bioinformatics/btw691.
25. Fortin, J.-P., Labbe, A., Lemire, M., Zanke, B.W., Hudson, T.J., Fertig, E.J., Greenwood, C.M., and Hansen, K.D. (2014). Functional normalization of 450k methylation array data improves replication in large cancer studies. Genome Biol. *15*, 503. https://doi.org/10.1186/s13059-014-0503-2.
26. Chen, Y., Lemire, M., Choufani, S., Butcher, D.T., Grafodatskaya, D., Zanke, B.W., Gallinger, S., Hudson, T.J., and Weksberg, R. (2013). Discovery of cross-reactive probes and polymorphic CpGs in the illumina infinium humanMethylation450 microarray. Epigenetics *8*, 203–209. https://doi.org/10.4161/epi.23470.
27. McCartney, D.L., Walker, R.M., Morris, S.W., McIntosh, A.M., Porteous, D.J., and Evans, K.L. (2016). Identification of polymorphic and off-target probe binding sites on the illumina infinium methylationEPIC BeadChip. Genom. Data *9*, 22–24. https://doi.org/10.1016/j.gdata.2016.05.012.
28. Pidsley, R., Zotenko, E., Peters, T.J., Lawrence, M.G., Risbridger, G.P., Molloy, P., Van Djik, S., Muhlhausler, B., Stirzaker, C., and Clark, S.J. (2016). Critical evaluation of the Illumina MethylationEPIC BeadChip microarray for whole-genome DNA methylation profiling. Genome Biol. *17*, 208. https://doi.org/10.1186/s13059-016-1066-1.
29. Du, P., Zhang, X., Huang, C.-C., Jafari, N., Kibbe, W.A., Hou, L., and Lin, S.M. (2010). Comparison of Beta-value and

M-value methods for quantifying methylation levels by microarray analysis. BMC Bioinf. *11*, 587. https://doi.org/10.1186/1471-2105-11-587.

30. den Hollander, W., Ramos, Y.F.M., Bos, S.D., Bomer, N., van der Breggen, R., Lakenberg, N., de Dijcker, W.J., Duijnisveld, B.J., Slagboom, P.E., Nelissen, R.G.H.H., and Meulenbelt, I. (2014). Knee and hip articular cartilage have distinct epigenomic landscapes: implications for future cartilage regeneration approaches. Ann. Rheum. Dis. *73*, 2208–2212. https://doi.org/10.1136/annrheumdis-2014-205980.
31. Edgar, R., Domrachev, M., and Lash, A.E. (2002). Gene Expression Omnibus: NCBI gene expression and hybridization array data repository. Nucleic Acids Res. *30*, 207–210. https://doi.org/10.1093/nar/30.1.207.
32. Johnson, W.E., Li, C., and Rabinovic, A. (2007). Adjusting batch effects in microarray expression data using empirical Bayes methods. Biostatistics *8*, 118–127. https://doi.org/10.1093/biostatistics/kxj037.
33. Lê, S., Josse, J., and Husson, F. (2008). FactoMineR: an R package for multivariate analysis. J. Stat. Software *25*, 1–18. https://doi.org/10.18637/jss.v025.i01.
34. Ritchie, M.E., Phipson, B., Wu, D., Hu, Y., Law, C.W., Shi, W., and Smyth, G.K. (2015). Limma powers differential expression analyses for RNA-sequencing and microarray studies. Nucleic Acids Res. *43*, e47. https://doi.org/10.1093/nar/gkv007.
35. Suderman, M., Staley, J.R., French, R., Arathimos, R., Simpkin, A., and Tilling, K. (2018). dmrff: identifying differentially methylated regions efficiently with power and control. Preprint at bioRxiv. https://doi.org/10.1101/508556.
36. Maksimovic, J., Oshlack, A., and Phipson, B. (2021). Gene set enrichment analysis for genome-wide DNA methylation data. Genome Biol. *22*, 173. https://doi.org/10.1186/s13059-021-02388-x.
37. Phipson, B., Maksimovic, J., and Oshlack, A. (2016). missMethyl: an R package for analyzing data from Illumina's HumanMethylation450 platform. Bioinformatics *32*, 286–288. https://doi.org/10.1093/bioinformatics/btv560.
38. Shabalin, A.A. (2012). Matrix eQTL: ultra fast eQTL analysis via large matrix operations. Bioinformatics *28*, 1353–1358. https://doi.org/10.1093/bioinformatics/bts163.
39. Sul, J.H., Han, B., Ye, C., Choi, T., and Eskin, E. (2013). Effectively identifying eQTLs from multiple tissues by combining mixed model and meta-analytic approaches. PLoS Genet. *9*, e1003491. https://doi.org/10.1371/journal.pgen.1003491.
40. Min, J.L., Hemani, G., Hannon, E., Dekkers, K.F., Castillo-Fernandez, J., Luijk, R., Carnero-Montoro, E., Lawson, D.J., Burrows, K., Suderman, M., et al.; BIOS Consortium (2021). Genomic and phenotypic insights from an atlas of genetic effects on DNA methylation. Nat. Genet. *53*, 1311–1321. https://doi.org/10.1038/s41588-021-00923-x.
41. Tachmazidou, I., Hatzikotoulas, K., Southam, L., Esparza-Gordillo, J., Haberland, V., Zheng, J., Johnson, T., Koprulu, M., Zengini, E., Steinberg, J., et al. (2019). Identification of new therapeutic targets for osteoarthritis through genome-wide analyses of UK Biobank data. Nat. Genet. *51*, 230–236. https://doi.org/10.1038/s41588-018-0327-1.
42. Willer, C.J., Li, Y., and Abecasis, G.R. (2010). METAL: fast and efficient meta-analysis of genomewide association scans. Bioinformatics *26*, 2190–2191. https://doi.org/10.1093/bioinformatics/btq340.
43. Hemani, G., Zheng, J., Elsworth, B., Wade, K.H., Haberland, V., Baird, D., Laurin, C., Burgess, S., Bowden, J., Langdon, R., et al. (2018). The MR-Base platform supports systematic causal inference across the human phenome. Elife *7*, e34408. https://doi.org/10.7554/eLife.34408.
44. Giambartolomei, C., Vukcevic, D., Schadt, E.E., Franke, L., Hingorani, A.D., Wallace, C., and Plagnol, V. (2014). Bayesian test for colocalisation between pairs of genetic association studies using summary statistics. PLoS Genet. *10*, e1004383. https://doi.org/10.1371/journal.pgen.1004383.
45. Rushton, M.D., Reynard, L.N., Barter, M.J., Refaie, R., Rankin, K.S., Young, D.A., and Loughlin, J. (2014). Characterization of the cartilage DNA methylome in knee and hip osteoarthritis. Arthritis Rheumatol. *66*, 2450–2460. https://doi.org/10.1002/art.38713.
46. Schegg, B., Hülsmeier, A.J., Rutschmann, C., Maag, C., and Hennet, T. (2009). Core glycosylation of collagen is initiated by two β(1-O)Galactosyltransferases. Mol. Cell Biol. *29*, 943–952. https://doi.org/10.1128/MCB.02085-07.
47. Kehayova, Y.S., Watson, E., Wilkinson, J.M., Loughlin, J., and Rice, S.J. (2021). Genetic and Epigenetic Interplay Within a COLGALT2 Enhancer Associated With Osteoarthritis. Arthritis Rheumatol. https://doi.org/10.1002/art.41738.
48. Shi, Q., Xiong, B., Zhong, J., Wang, H., Ma, D., and Miao, C. (2017). MFHAS1 suppresses TLR4 signaling pathway via induction of PP2A C subunit cytoplasm translocation and inhibition of c-Jun dephosphorylation at Thr239. Mol. Immunol. *88*, 79–88. https://doi.org/10.1016/j.molimm.2017.06.017.
49. Zhong, J., Shi, Q.-Q., Zhu, M.-M., Shen, J., Wang, H.-H., Ma, D., and Miao, C.-H. (2015). MFHAS1 is associated with sepsis and stimulates TLR2/NF-κB signaling pathway following negative regulation. PLoS One *10*, e0143662. https://doi.org/10.1371/journal.pone.0143662.
50. Liu-Bryan, R. (2013). Synovium and the innate inflammatory network in osteoarthritis progression. Curr. Rheumatol. Rep. *15*, 323. https://doi.org/10.1007/s11926-013-0323-5.
51. Price, J.S., Waters, J.G., Darrah, C., Pennington, C., Edwards, D.R., Donell, S.T., and Clark, I.M. (2002). The role of chondrocyte senescence in osteoarthritis. Aging Cell *1*, 57–65. https://doi.org/10.1046/j.1474-9728.2002.00008.x.
52. Goldring, S.R., and Goldring, M.B. (2016). Changes in the osteochondral unit during osteoarthritis: structure, function and cartilage-bone crosstalk. Nat. Rev. Rheumatol. *12*, 632–644. https://doi.org/10.1038/nrrheum.2016.148.
53. Mokuda, S., Nakamichi, R., Matsuzaki, T., Ito, Y., Sato, T., Miyata, K., Inui, M., Olmer, M., Sugiyama, E., Lotz, M., and Asahara, H. (2019). Wwp2 maintains cartilage homeostasis through regulation of Adamts5. Nat. Commun. *10*, 2429. https://doi.org/10.1038/s41467-019-10177-1.
54. Südhof, T.C. (2012). The presynaptic active zone. Neuron *75*, 11–25. https://doi.org/10.1016/j.neuron.2012.06.012.
55. Scanzello, C.R., and Goldring, S.R. (2012). The role of synovitis in osteoarthritis pathogenesis. Bone *51*, 249–257. https://doi.org/10.1016/j.bone.2012.02.012.
56. Ahmad, M., Srinivasula, S.M., Wang, L., Talanian, R.V., Litwack, G., Fernandes-Alnemri, T., and Alnemri, E.S. (1997). CRADD, a novel human apoptotic adaptor molecule for caspase-2, and FasL/tumor necrosis factor receptor-interacting protein RIP. Cancer Res. *57*, 615–619.
57. Huang, H., Zheng, J., Shen, N., Wang, G., Zhou, G., Fang, Y., Lin, J., and Zhao, J. (2018). Identification of pathways and genes associated with synovitis in osteoarthritis using

bioinformatics analyses. Sci. Rep. *8*, 10050. https://doi.org/10.1038/s41598-018-28280-6.

58. Varghese, S., Rydziel, S., Jeffrey, J.J., and Canalis, E. (1994). Regulation of interstitial collagenase expression and collagen degradation by retinoic acid in bone cells. Endocrinology *134*, 2438–2444. https://doi.org/10.1210/endo.134.6.8194470.
59. Shlopov, B.V., Lie, W.R., Mainardi, C.L., Cole, A.A., Chubinskaya, S., and Hasty, K.A. (1997). Osteoarthritic lesions: involvement of three different collagenases. Arthritis Rheum. *40*, 2065–2074. https://doi.org/10.1002/art.1780401120.
60. Thielen, N.G.M., van der Kraan, P.M., and van Caam, A.P.M. (2019). TGFβ/BMP signaling pathway in cartilage homeostasis. Cells *8*, 969. https://doi.org/10.3390/cells8090969.
61. Saharinen, J., Taipale, J., and Keski-Oja, J. (1996). Association of the small latent transforming growth factor-beta with an eight cysteine repeat of its binding protein LTBP-1. EMBO J. https://doi.org/10.1002/j.1460-2075.1996.tb00355.x.

The American Journal of Human Genetics, Volume *109*

Supplemental information

An epigenome-wide view of osteoarthritis

in primary tissues

Peter Kreitmaier, Matthew Suderman, Lorraine Southam, Rodrigo Coutinho de Almeida, Konstantinos Hatzikotoulas, Ingrid Meulenbelt, Julia Steinberg, Caroline L. Relton, J. Mark Wilkinson, and Eleftheria Zeggini

Supplemental Material

Supplemental Figures:

Figure S1

EWAS reveal hyper-and hypomethylated regions Volcano plot visualises the beta coefficients of methylated regions. Black dots indicate significant DMRs (Bonferroni adjusted p values < 0.05) between low-grade and high-grade osteoarthritis cartilage. We identified 1,701 hypermethylated and 776 hypomethylated DMRs.

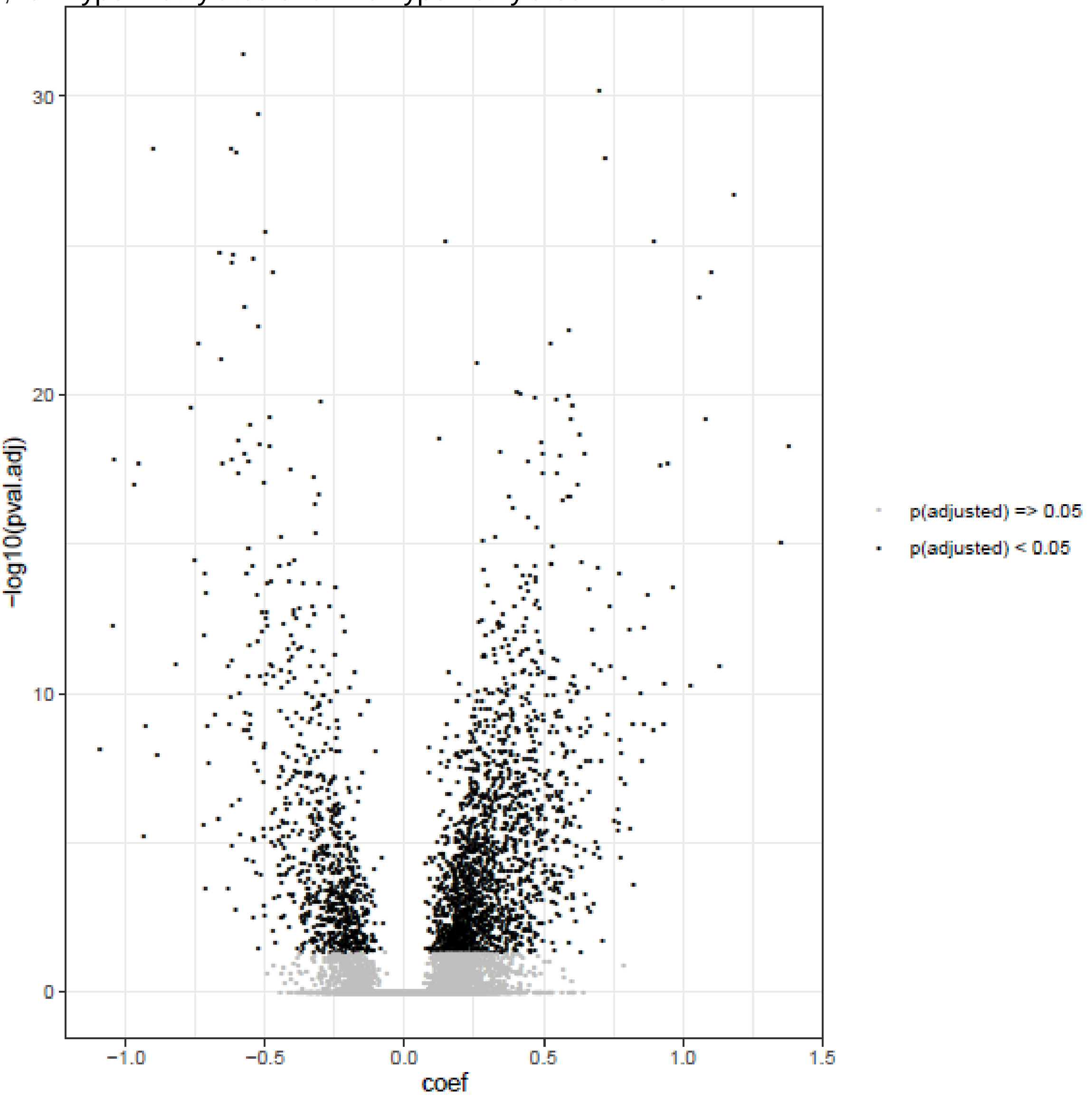

Figure S2

Pathway analysis reveals enrichment of four KEGG terms. Most significant KEGG terms enriched in 15,328 DMSs. Red dashed lines indicate the significance threshold (Benjamini-Hochberg adjusted $p < 0.05$).

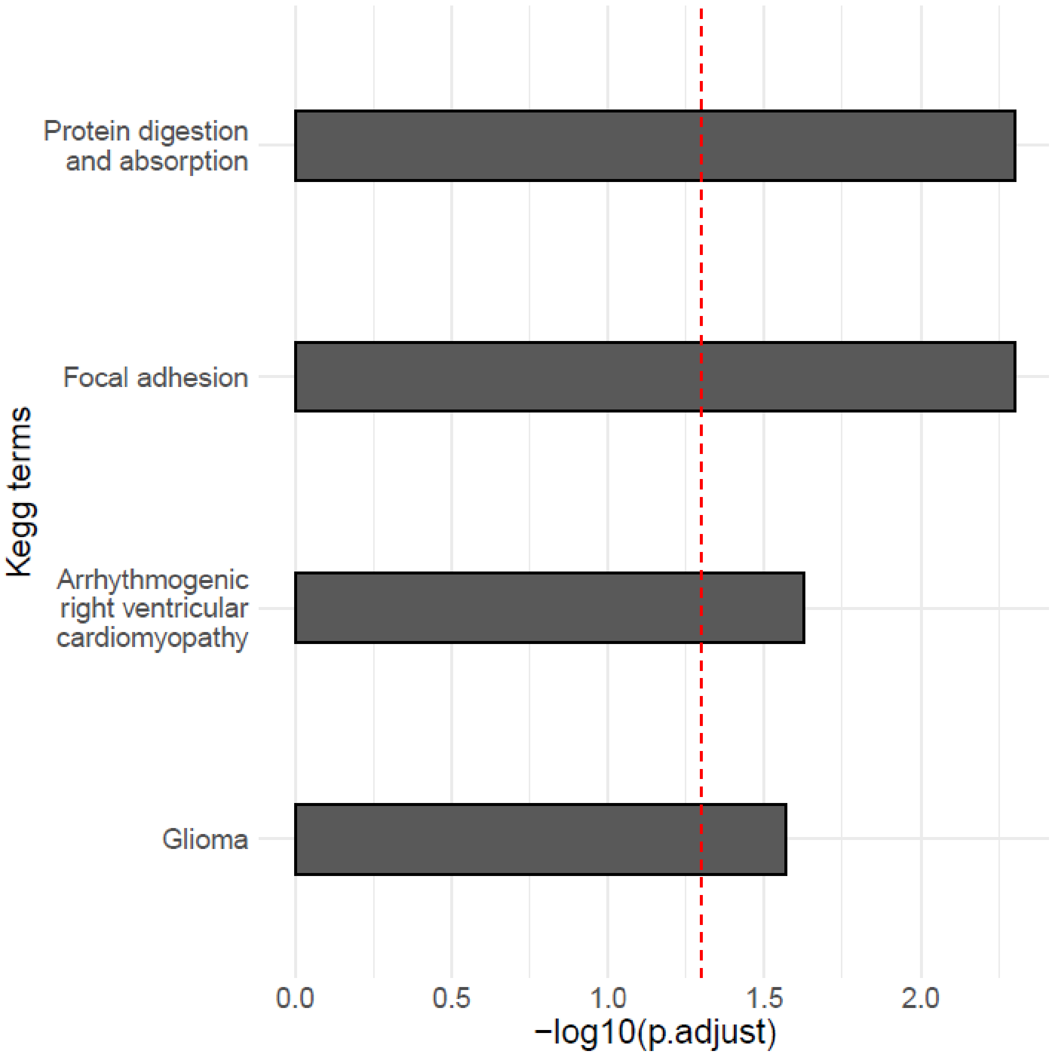

Figure S3

ML-based classifier distinguishes low-grade and high-grade osteoarthritis cartilage

Using cross-validation, we trained and tested Random-Forest based classifiers that distinguish low-grade from high-grade osteoarthritis cartilage samples, the resulting ROC curve is depicted. The area under the curve is 0.967

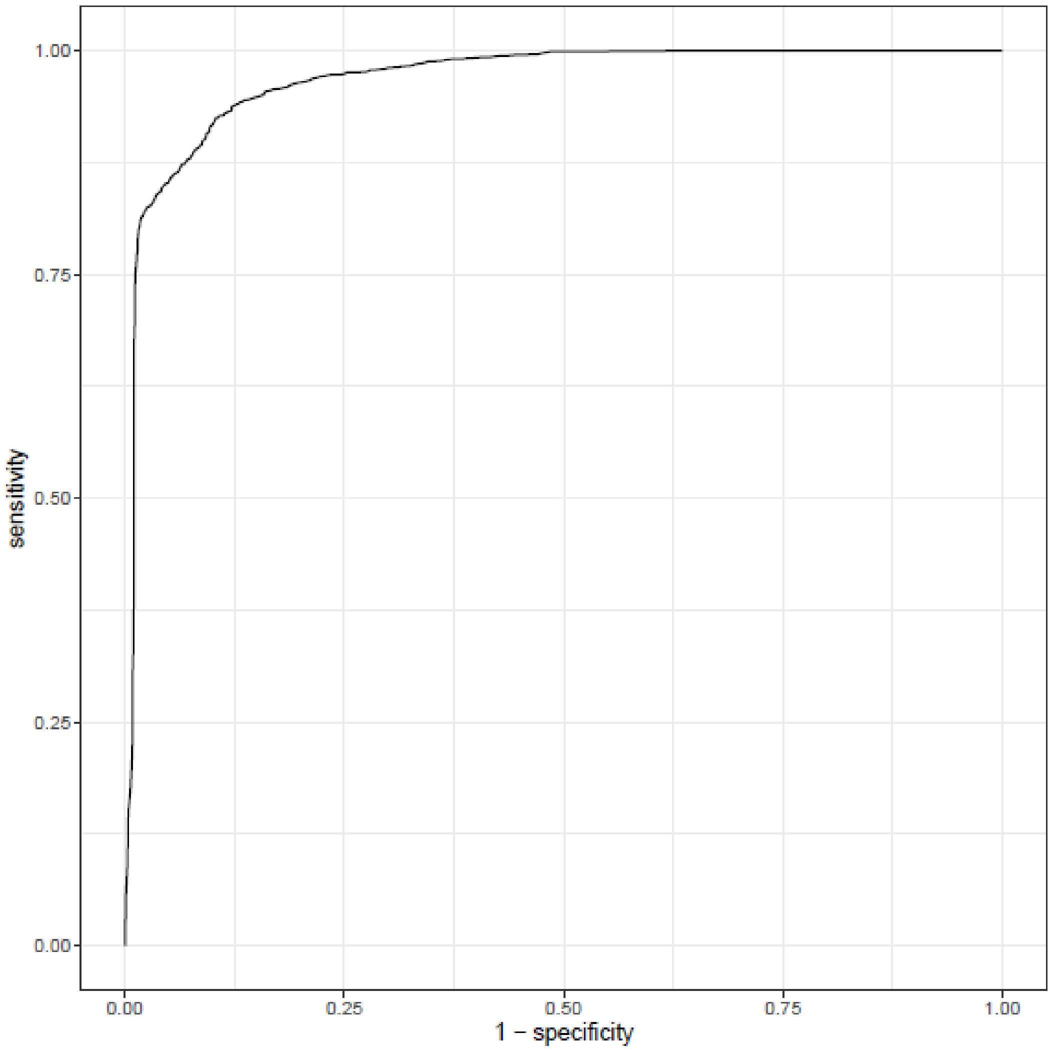

Figure S4

The mQTL landscape in cartilage and synovium Manhattan plots depicting the lowest association p value per SNP with close methylation sites (< 1 Mb) in low-grade osteoarthritis cartilage (A), high-grade osteoarthritis cartilage (B) and synovium (C) on genome-wide scale. The red lines indicate genome-wide significance (Bonferroni p < 0.05).

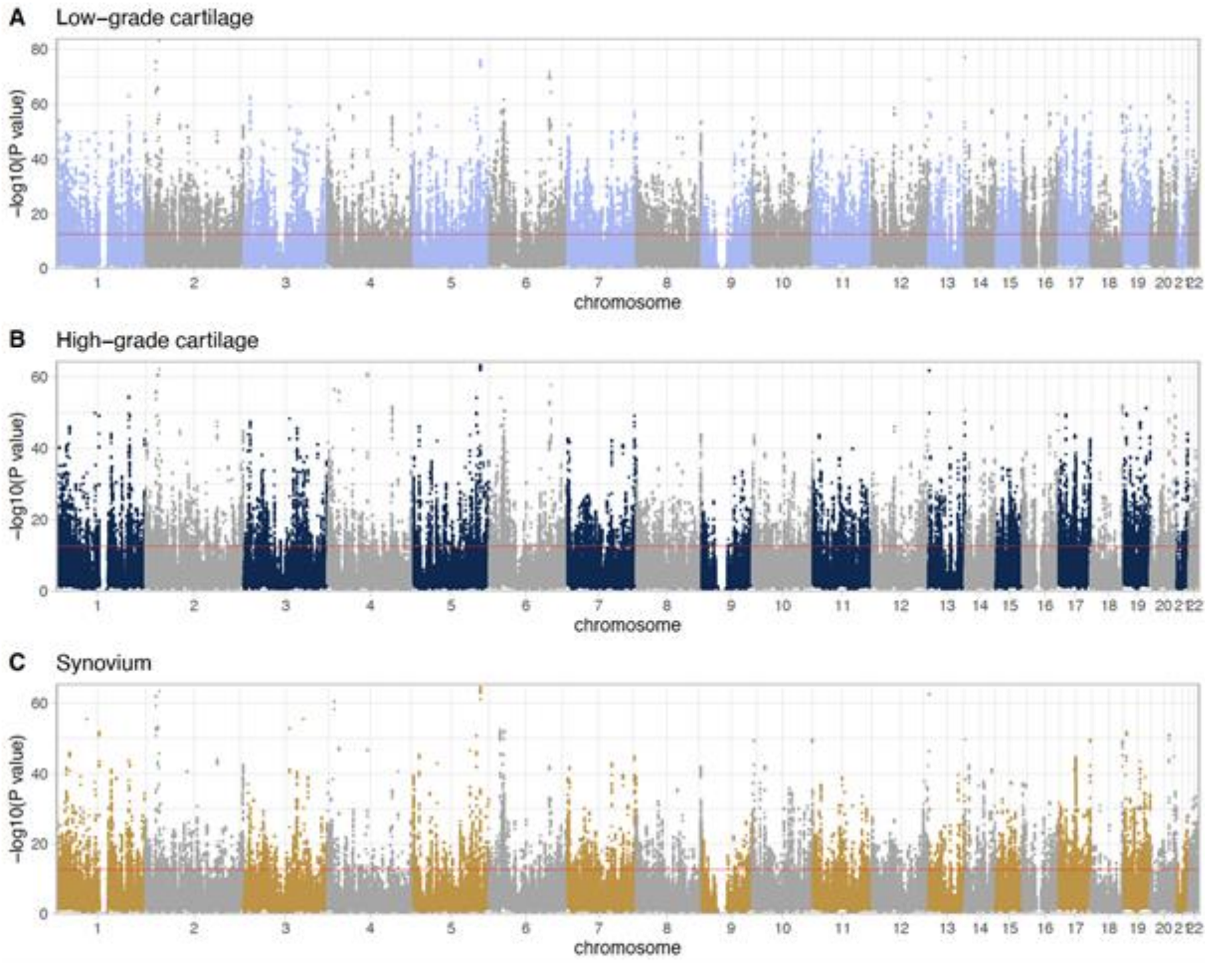

Figure S5

Insights into the mQTL architecture in low-grade osteoarthritis cartilage. Barplots showing the proportions (A) and enrichments (C) of gene annotations of methylation sites with at least one mQTL in low-grade osteoarthritis cartilage. Analogously, barplots B and D describe proportions and enrichments of CpG island annotations, respectively. In plot C and D, all enrichments pass the respective Bonferroni threshold (C: $p < 0.0071$, D: $p < 0.0083$). Plot E describes the number of targeted methylation sites across mQTL, showing that most mQTL are associated with few methylation sites. Similarly, plot F describes the number of mQTL across methylation sites, showing that most methylation sites have a few mQTLs.
The dashed blue line in plots C and D refers to the fold enrichment of 1. Msite = methylation site; TSS: Transcription start site; UTR: Untranslated region; Island: Methylation site within a CpG island; N_Shore and S_Shore: Methylation site within 2kb upstream and downstream around the starting site of a CpG island, respectively; N_Shelf and S_Shelf: Methylation site between 2kb and 4kb upstream and downstream of a CpG island, respectively; OpenSea: Methylation sites further then 4 kb away from a CpG Island.

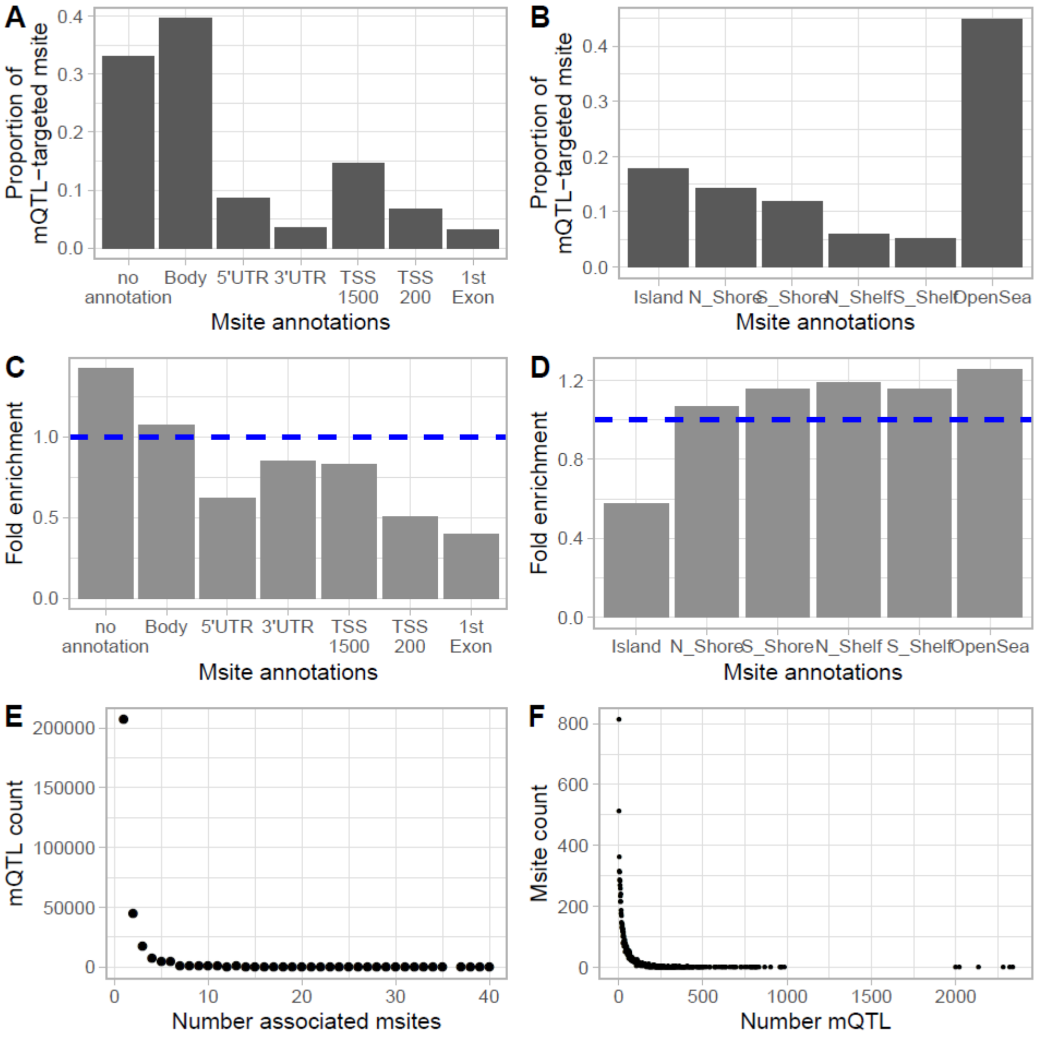

Figure S6
Insights into the mQTL architecture in high-grade osteoarthritis cartilage
Analog to Figure S5, but for the mQTL profile in high-grade osteoarthritis cartilage. In plot C and D, all enrichments pass the respective Bonferroni threshold (C: $p < 0.0071$, D: $p < 0.0083$). Msite = methylation site

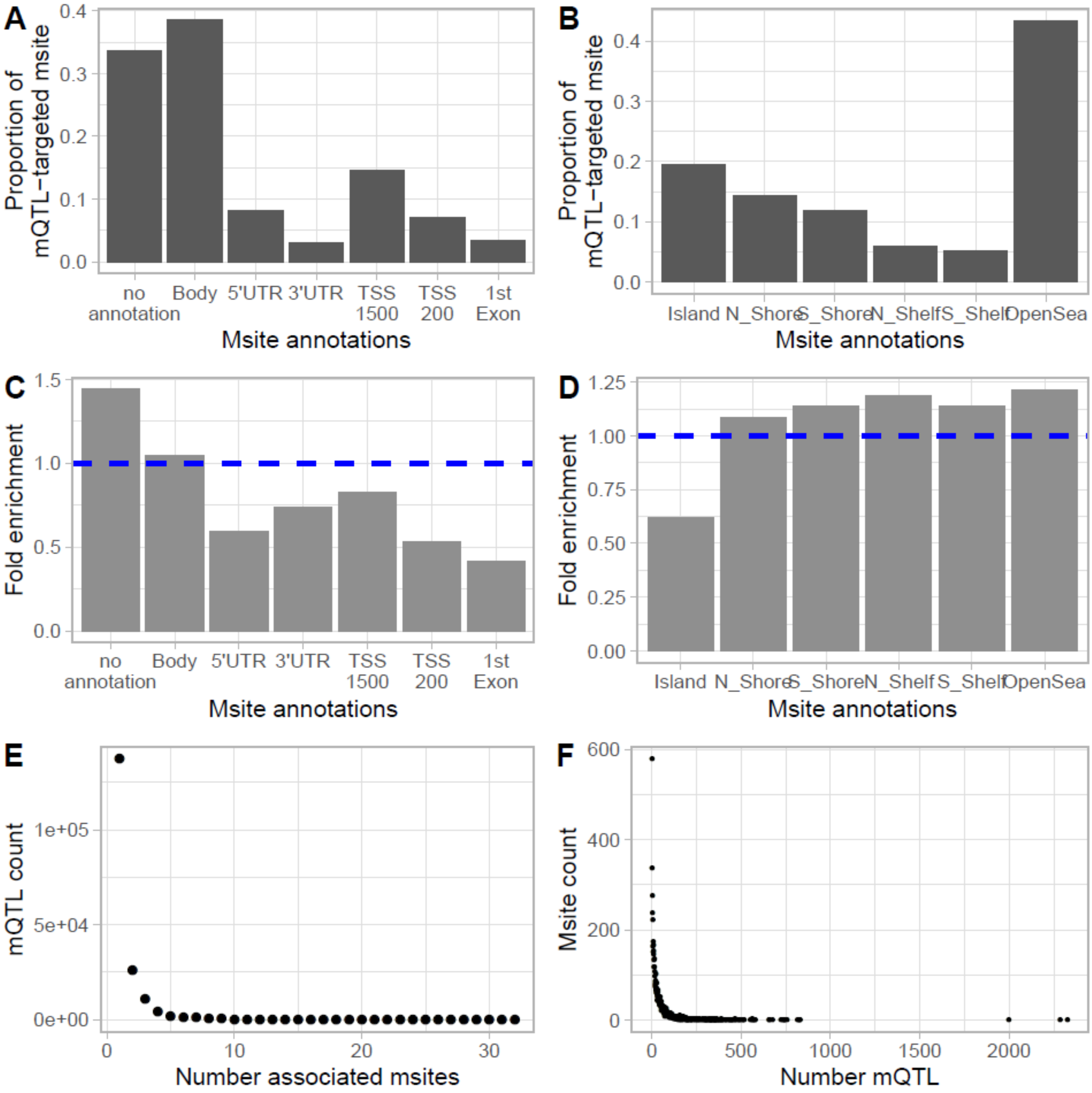

Figure S7

Insights into the mQTL architecture in synovium

Analyses analog to Figure S5, but for methylation sites targeted in synovium. In plots C and D, enrichments passing the respective Bonferroni threshold (C: $p < 0.0071$, D: $p < 0.0083$) are dark grey, otherwise light grey.

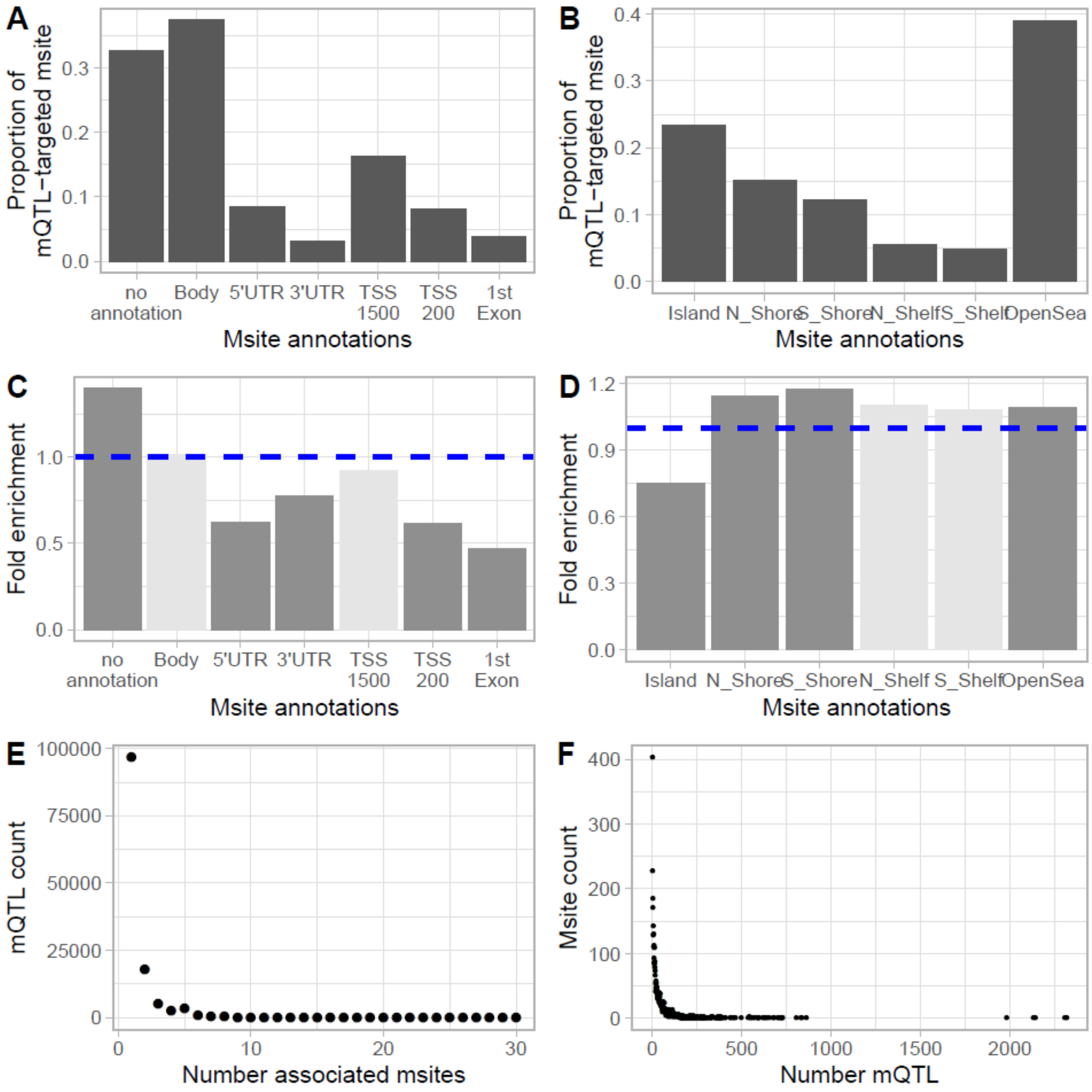

Figure S8

Most significant, sex-specific mQTL effects in thre osteoarthritis tissues. The boxplots describe the most signficiant, sex-specific mQTL in (A) low-grade (beta = 2.32, SE = 0.19, FDR = 6.02 x10^{-12}) and (B) high-grade osteoarthritis cartilage (beta = 3.10, SE = 0.25, FDR = 2.95x10^{-11}) as well as in (C) synovium (beta = 2.90, SE = 0.14, FDR = 1.21x10^{-23}) (C). The boxplots represent 25th, 50th and 75th percentiles, and whiskers extend to 1.5 times the interquartile range.

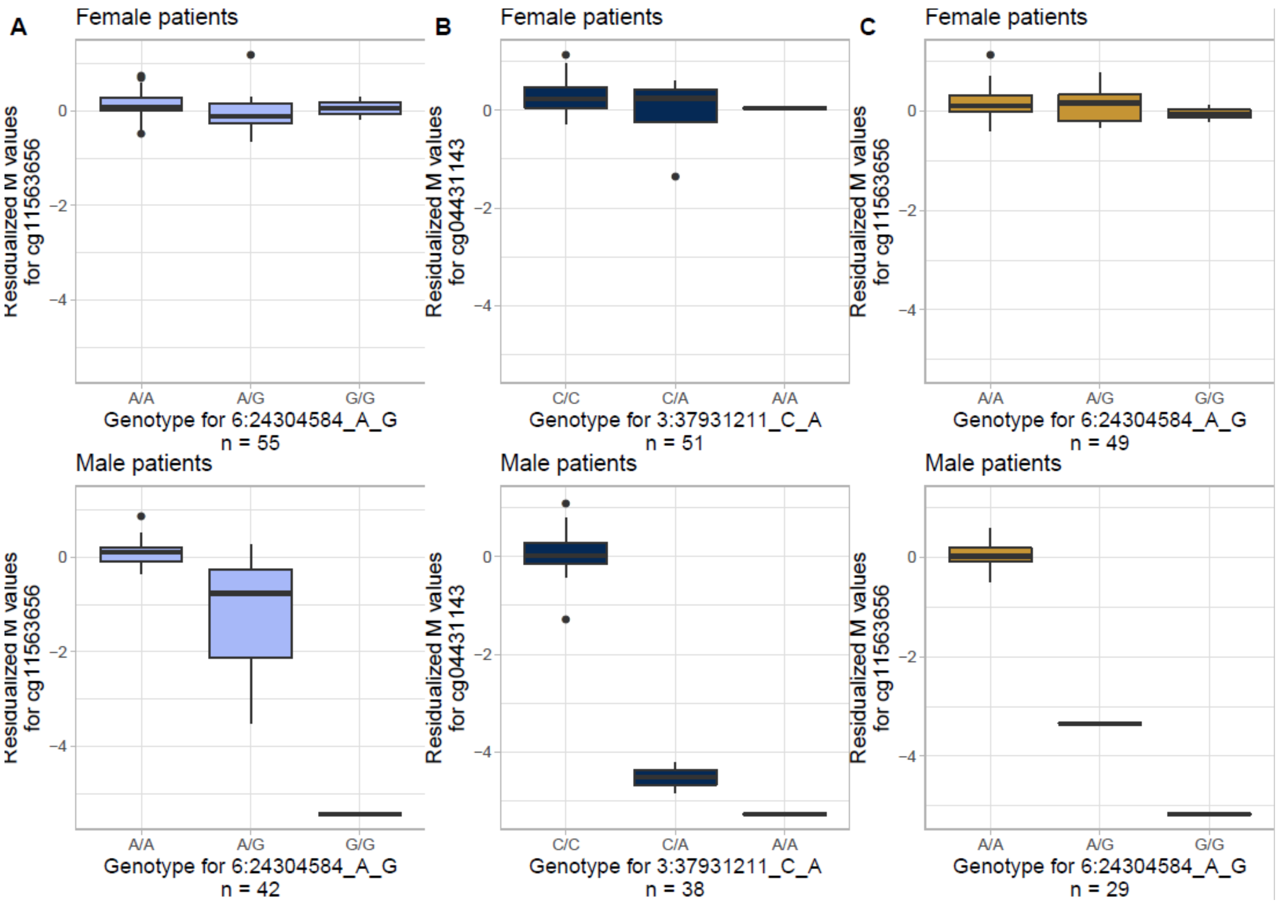

Figure S9

An overview of the Mendelian randomization approach. We estimated the putative causal effect of a methylation site (exposure) on osteoarthritis (outcome). We preselected 15,328 methylation sites which we previously linked to cartilage degeneration in the EWAS. Subsequently, we performed the 2SMR approach per tissue (low-grade osteoarthritis cartilage, high-grade osteoarthritis cartilage and synovium). Of the 15,328 methylation sites, we retained the ones with at least one significant mQTL (FDR < 0.05) and used these mQTLs as instrumental variables (IV) to estimate the putative causal effect of the methylation sites (exposure) on three osteoarthritis-relevant traits. For methylation sites with exactly one independent IV, we performed the Wald-ratio test for methylation sites, otherwise the inverse-variance-weighted (IVW) method

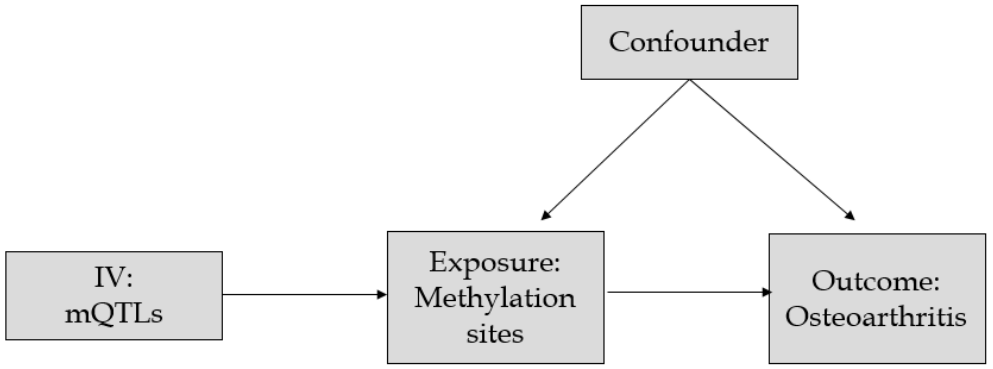

Figure S10

An overview of the Mendelian randomization approach in the reverse direction. We sought to estimate the putative causal effect of osteoarthritis (exposure) on methylation (outcome). We tested every osteoarthritis trait-methylation site trait combination (low-grade osteoarthritis cartilage: n = 10,099, high-grade osteoarthritis cartilage: n = 6,110, synovium: n = 4,662) that we examined in the opposite direction (Figure S9). We used GWAS risk SNPs as instrumental variables (all OA: n = 27, knee OA: n = 10, TKR: n = 4). We applied the IVW method.

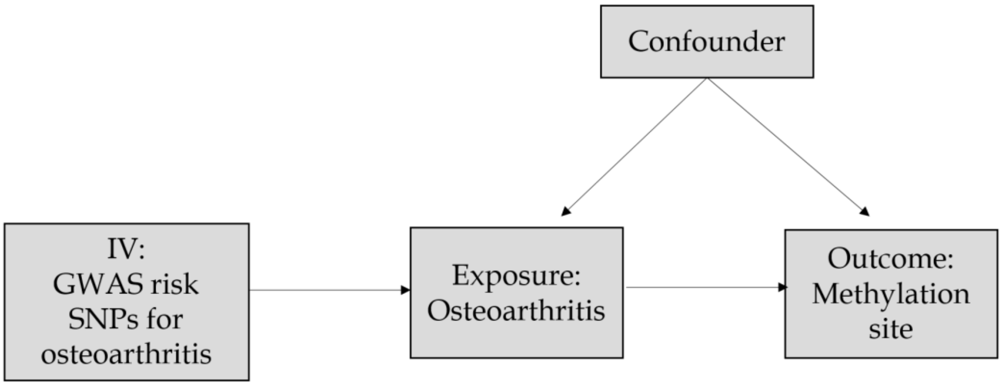

Supplemental Tables:

Table S1. Overview of patient characteristics per tissue, related to the Subjects and Methods
Table S2. List of 15,328 differentially methylated sites, related to Epigenome-wide association study reveals widespread, robustly-replicating signals
Table S3. List of 2,477 differentially methylated regions, related to Epigenome-wide association study reveals widespread, robustly-replicating signals
Table S4. Biological terms that are enriched among 15,328 differentially methylated sites, related to Epigenome-wide association study reveals widespread, robustly-replicating signals
Table S5. Biological terms that are enriched among 7,192 replicated differentially methylated sites, related to Epigenome-wide association study reveals widespread, robustly-replicating signals
Table S6. Numbers of significant mQTL effects per tissue, related to Genome-wide mQTL maps in osteoarthritis-relevant tissues
Table S7. Overview of sex-specific mQTL (FDR < 0.05) in low-grade OA cartilage, related to Genome-wide mQTL maps in osteoarthritis-relevant tissues
Table S8. Overview of sex-specific mQTL (FDR < 0.05) in high-grade OA cartilage, related to Genome-wide mQTL maps in osteoarthritis-relevant tissues
Table S9. Overview of sex-specific mQTL (FDR < 0.05) in synovium, related to Genome-wide mQTL maps in osteoarthritis-relevant tissues
Table S10. List of 195 differential mQTLs, related to figure 5 and Identification of grade-specific mQTLs in cartilage
Table S11. List of significant Mendelian randomization results, related to figure 6 and Assessing the causal role of methylation in osteoarthritis

Table S12. List of colocalising GWAS and mQTL signals, related to figure 7 and Resolution of GWAS signals
Table S13. List of putative causal effects of methylation on gene expression in joint tissues, related to Resolution of GWAS signals

Supplemental Notes

Supplemental Note 1: Power analysis for differential analysis in osteoarthritis cartilage

Based on RNA-seq data of matching low-grade and high-grade OA cartilage samples, the power of this study (n = 90 patients) to detect differentially expressed genes is 70 % at FDR 5% (Steinberg and Zeggini, 2016). This is a substantial improvement compared to the next largest study (which was 17 knee OA samples) with the predicted power of ~20% at the same significance level.

Supplemental Note 2: Epigenome-wide association study reveals widespread, robustly-replicating signals

To identify DNA methylation markers of cartilage degeneration, we performed an epigenome-wide association study (EWAS) on paired low-grade and high-grade osteoarthritis cartilage samples from 90 patients across 401,870 methylation sites. We identified 15,328 differentially methylated sites (DMS) distributed across the whole genome (significance threshold of $p < 1.24 \times 10^{-7}$; Methods), which constitutes 3.81% of all tested sites (Figure 2 A upper panel, Table S1). Of these DMSs, 8,783 (57%) and 6,545 (43%) showed hypermethylation and hypomethylation in high-grade osteoarthritis cartilage, respectively (Figure 2 B and C). We found a significant overrepresentation of hypermethylated sites among DMSs (binomial test $p = 1.57 \times 10^{-73}$).

In epigenome-wide association studies, it is common to estimate differentially methylated regions (DMRs). As methylation levels of proximal cytosines tend to correlate with each other (Eckhardt et al., 2006), this approach enables dimensionality and redundancy reduction and focuses on robust signals. We identified 2,477 DMRs (Bonferroni adjusted $p < 0.05$), each consisting of at least 2 methylation sites (Figure 2 A bottom panel, Table S2). Of these, 1,701 (69%) and 776 (31%) DMRs were hyper- and hypomethylated in high-grade osteoarthritis cartilage, respectively (Figure S1). The mean length of DMRs was 211.74 (sd: 198.36). The shortest and longest DMR is three and 1,608 bp long, respectively.

We conducted pathway enrichment analyses for the 15,328 DMS. We identified significant over-representations (Benjamini-Hochberg adjusted $p < 0.05$) for 29 Gene ontology (GO) and 4 KEGG terms (Table S3). The five most significant GO and four KEGG terms are shown in Figure 2 D and Figure S2. The GO enrichment analysis revealed biological processes that have been previously linked to osteoarthritis, including signals linked to external matrix organization (Rushton et al., 2014), skeletal system development (Bonin et al., 2016; Rushton et al., 2014; Steinberg et al., 2017; Zhang et al., 2016), cell adhesion (Alvarez-Garcia et al., 2016; Steinberg et al., 2017), signaling pathways (e.g. integrin signaling pathway (Steinberg et al., 2017) and platelet activation (Steinberg et al., 2017). In addition, we identified for the first time enrichment of the epithelium-related term "positive regulation of epithelial cell migration" in articular cartilage which may point to a novel etiological mechanism. We did not identify enrichments among 2,477 DMRs.

To replicate our findings, we performed an EWAS between matched low-grade and high-grade osteoarthritis cartilage samples from an independent dataset comprising 17 knee osteoarthritis patients (den Hollander et al., 2014, 2015). As in the discovery analyses, we analysed data on the methylation site and on a regional level. On the site level, 13,420 DMSs

(87.6%) from the discovery data were present in the replication set. Of those, 13,001 (96.9%) showed the same direction of effect in the replication set, 7,192 (53.6%) at nominal significance ($p < 0.05$). The effect sizes of the 7,192 DMSs in the discovery and replication sets were highly correlated (Pearson r = 0.96, $p < 2.2x10^{-16}$) (Figure 3A). Of these replicated DMSs, 3,231 (44.92%) and 3,961 (55.07%) were hypermethylated and hypomethylated in high-grade osteoarthritis cartilage, respectively. A binomial test showed a significant overrepresentation of hypomethylated sites among replicated DMSs ($p = 3.87x10^{-18}$). At the region level, we conservatively considered methylated regions that are composed of exactly the same methylation sites in the discovery and replication set. Given this definition, 105 of 2,477 DMRs (4.2%) were present in the replication set. For all of these, the effects in the replication set were in the same direction as the discovery set and all were nominally significant ($p < 0.05$). Furthermore, the effect sizes of these 105 replicating DMRs set were highly correlated between the discovery and replication datasets (Pearson r: 0.95, p-value < $2.2x10^{-16}$) (Figure 3B). These results point to the robustness of the identified methylation changes. Performing pathway enrichment analysis on 7,192 replicated DMSs identified 18 GO terms (Table S5) and highlighted pathways related to skeletal system development, cell adhesion and lipid metabolic processes.

To assess sex-specific markers of cartilage degeneration, we performed EWAS separately on paired low-grade and high-grade cartilage in female (n = 52) and male (n = 38) patients.
In female patients, we identified 49,695 differentially (FDR < 0.05) methylated sites (DMS). A number of 23,494 and 26,201 showed hypo- and hypermethylation in high-grade cartilage samples, respectively. In male patients, we identified 44,059 DMS (21,355 and 22,704 are hypo- and hypermethylated in high-grade cartilage, respectively).
Of note, we identified methylation sites that show sex-specific effects. In female patients, 1,338 methylation sites showed effects with FDR < 0.05, but $p > 0.05$ in men and in the combined analysis. Analogously, we identified 3,316 methylation sites with male-specific effects. These results suggest sex-specific effects in osteoarthritis cartilage.

Supplemental Note 3: Overlap between mQTL maps and loci of previously reported mQTLs

We tested whether the mQTL maps of this study revealed genetic-epigenetic effects in regions of previously reported mQTLs in osteoarthritis-relevant tissue (Aubourg et al., 2021; Boer et al., 2021). More specifically, we sought to overlap the mQTL maps with 25 and 5 regions of previously identified mQTL effects (+/- 500 kb) in cartilage and synovium, respectively.
In low-grade osteoarthritis cartilage, we identified at least one significant mQTL effect in our mQTL map in 24 of 25 tested regions. These regions contained 28 previously reported mQTLs (Bonferroni $p < 0.05$)
In high-grade osteoarthritis cartilage, we found at least one significant mQTL effect in our mQTL map in 20 of 25 tested regions. These regions contained 28 previously reported mQTLs (Bonferroni $p < 0.05$)
In synovium we determined at least one significant mQTL effect in our mQTL map in all 5 tested regions. These regions contained 4 previously reported mQTLs (Bonferroni $p < 0.05$)

Supplemental Note 4: Sensitivity analysis of colocalisation: comparison between osteoarthritis tissues and peripheral blood

Performing the colocalisation between osteoarthritis GWAS and whole blood mQTL data (section "Comparing colocalization of osteoarthritis loci in joint and whole blood mQTL data") involved fewer variants due to the smaller set of overlapping variants. This could limit the ability to identify colocalising signals, making it unclear whether we can attribute the missing signal to the tissue specificity of the mQTL data. To account for this, we again performed colocalisation between joint mQTL and GWAS data, this time on reduced variant sets (same

variant number as between whole blood mQTL and GWAS data). We then filtered for risk variant-methylation site pairs that showed evidence for colocalisation between osteoarthritis GWAS and (1) joint mQTL data when considering a reduced variant set, but (2) not in whole blood.

Using this strict filtering, we found three all OA variant-methylation site pairs (involving the all OA risk variants rs798726, rs12154055 and rs2856821) and one knee OA variant-methylation site pair (involving the knee OA risk variant rs9277552). Furthermore, we found one knee OA variant-methylation site pair (involving the knee OA risk variant rs56116847) that colocalises in synovium, but not in whole blood.

For example, risk variant rs798726 colocalised with methylation site cg07929082 in low-grade osteoarthritis cartilage with a high probability (93.9% and 97.2% considering the largest possible and a reduced variant set, respectively). However, in whole blood, the colocalisation probability was very low (0.000008%).

Supplemental Subjects and Methods

RESOURCES TABLE

REAGENT or RESOURCE	SOURCE	IDENTIFIER
**Deposited data**		
Summary statistics of differential methylation analysis, Mendelian randomization and methylation QTL data		https://hmgubox.helmholtz-muenchen.de/d/a23fce319fd844d4b293/
Full summary statistics of the methylation QTL analysis		http://mskkp.org
Replication data set	(den Hollander et al., 2014)	https://www.ncbi.nlm.nih.gov/geo/ id: GSE63106
**Software and algorithms**		
R		version 3.5.3
meffil	(Min et al., 2018)	R package, version 1.0.0
minfi	(Aryee et al., 2014; Fortin et al., 2017)	R package, version 1.28.4
IlluminaHumanMethylation450kanno.ilmn12.hg19		R package, version 0.6.0
limma	(Ritchie et al., 2015)	R package, version 3.38.3
sva	(Leek et al., 2012)	R package, version 3.30.1
dmrff	(Suderman et al., 2018)	R package, version 0.0.2
missMethyl	(Maksimovic et al., 2021)	R package, version 1.24.0
MatrixEQTL	(Shabalin, 2012)	R package, version 2.2
vcfR	(Knaus and Grünwald, 2017)	R package, version 1.8.0.
MetaTissue	(Sul et al., 2013)	http://genetics.cs.ucla.edu/metatissue/ Software, version 0.5
GWAS catalog		https://www.ebi.ac.uk/gwas/
GoDMC	(Min et al., 2021)	http://mqtldb.godmc.org.uk/
biomaRt	(Durinck et al., 2009)	R package, version 2.38.0
METAL	(Willer et al., 2010)	
TwoSampleMR	(Hemani et al., 2018)	R package, version 0.4.25

PLINK		Software, version 1.9
coloc.fast		https://github.com/tobyjohnson/gtx/blob/526120435bb3e29c39fc71604eee03a371ec3753/R/coloc.R
ivreg		R package version 0.6-1
FactoMineR	(Lê et al., 2008)	R package, version 2.0
caret		R package, version 6.0.84

Patients and study samples

Samples from osteoarthritis affected knees were collected in 101 patients that underwent total knee replacement due to late-stage osteoarthritis. The patients were collected in 3 cohorts. Cohort1 comprised 13 knee osteoarthritis patients (10 male and 3 female patients) with a mean age of 68, Cohort2 included 18 knee osteoarthritis patients (5 male and 13 female patients) with a mean age of 70 and Cohort 3 consisted of 70 knee osteoarthritis patients (28 male and 42 female patients) with a mean age of 70. Low-grade and high-grade osteoarthritis cartilage samples were collected from each patient, samples from the synovium of patients from Cohort2 and Cohort3. Cartilage samples were graded using the OARSI cartilage classification system (Cohort1) or International Cartilage Repair Society (ICRS) scoring system (Cohort2 and Cohort3; low-grade and high-grade osteoarthritis cartilage is signified by ICRS grades 0 or 1 and 3 or 4, respectively). This work was approved by Oxford NHS REC C (10/H0606/20 and 15/SC/0132), and samples were collected under Human Tissue Authority license 12182, Sheffield Musculoskeletal Biobank, University of Sheffield, UK. Before participating in the study, all patients provided written, informed consent.

Sample extraction

A previous study (Steinberg et al., 2021) reported the isolation of the chondrocytes (section "Isolation of chondrocytes"), the isolation of synoviocytes (section "Isolation of synoviocytes") and DNA extraction (section "DNA, RNA and protein extraction") in its methods part.

DNA methylation data

Genome-wide DNA methylation was measured using the Illumina 450k array in three batches. Batch 1 and 2 were generated using the Infinium HumanMethylation450 Bead Chip (450k array) batch 3 using the Illumina Infinium MethylationEPIC Bead Chip (EPIC array). We used the R package minfi to read idat files (Aryee et al., 2014; Fortin et al., 2017) and combined samples measured on 450k and EPIC arrays. We limited the methylation data to 452,832 methylation sites present on both array types. Based on genotype data, we identified three ethnicity outliers and removed methylation samples from these patients. We performed quality control per sample using the qc.summary function in the R package meffil (Min et al., 2018) and removed gender mismatches (2 samples), X-Y ratio outliers and samples with unbalanced ratios between methylated and unmethylated signals (9 samples). Moreover, we applied quality control functions implemented in minfi and discovered 1 additional methylation/unmethylation ratio outlier. To normalise the signal, we applied functional normalisation (Fortin et al., 2014), an approach that corrects for variation measured by the control probes on the array. Here, we considered the first 8 principal components explaining 95% of the variation in the 34 control probes. We removed probes on sex chromosomes, probes with detection pvalues of $p > 0.01$ in more than 5 % of the samples and previously reported cross-reactive probes (Chen et al., 2013; McCartney et al., 2016; Pidsley et al., 2016). Furthermore, we excluded probes that had been reported to overlap with common genetic variants (= polymorphic probes) as the signal of these probes might mirror genetic variation rather than true methylation signal (Chen et al., 2013). More specifically, we removed previously reported polymorphic methylation probes with common SNPs (MAF > 0.05, European population) directly located at the target methylated probe, at the single base

extension or on the probe body within 10 base pairs. Furthermore, we excluded samples that are technical replicates and that are not annotated to patients of cohorts 1-3. The resulting data comprised 401,870 methylation loci and 266 samples from 98 patients, including 90, 98 and 78 samples from low-grade osteoarthritis cartilage, high-grade osteoarthritis cartilage and synovium, respectively (Table S1). We conducted downstream statistical analyses on M-values as recommended (Du et al., 2010). To map methylation probe identifiers to their corresponding methylation sites and genomic positions (hg19) as well as close RefSeq genes, we used Illumina's annotation file version 1.2, provided by the R package IlluminaHumanMethylation450kanno.ilmn12.hg19.

DNA methylation data (replication set)

We included published methylation data for low-grade and high-grade osteoarthritis cartilage to replicate the findings of the EWAS and the ML-based classifiers (den Hollander et al., 2014). The data is publicly available in the Gene Expression Omnibus database (Edgar et al., 2002) and accessible through the entry number *GSE63106.* The replication data consists of matching low-grade and high-grade osteoarthritis cartilage samples from 31 patients who underwent total joint replacement to treat primary osteoarthritis (knee: 17 patients, hip: 14 patients). We downloaded the data matrix with the beta values per sample ("GSE63106_series_matrix.txt.gz") for 374,412 methylation sites. To ensure higher comparability, we converted these beta values to M-values and used them for all downstream analyses between discovery and replication analyses.

Genotype data

Genotypes were measured with the InfiniumCoreExome-12v1-1_A array or the InfiniumCoreExome-24v1-1_A array (supplemental subjects and methods). Genotype data were preprocessed as previously described (Steinberg et al., 2021).

Briefly, genotypes for *cohort1* were measured on the array InfiniumCoreExome-12v1-1_A array, *cohort2* and *cohort3* on the InfiniumCoreExome-24v1-1_A array. Genotype data were preprocessed as previously described (Steinberg et al., 2021). Briefly, we called the variants using the GenCall (Illumina) tool and mapped the genotypes to GRC37/hg19 by applying an online software (http://www.well.ox.ac.uk/~wrayner/strand/index.html). We then applied the same quality control procedure for both arrays. In short, we (1) removed samples and variants with a call rate < 90%, (2) correlated measured array genotypes to Fluidigm genotypes (no sample achieved a correlation < 0.95) and (3) removed samples using several filters (call rate < 98%, heterozygosity distribution outliers performed using 2 different minor allele frequency (MAF) bins with ≥ 1% MAF and < 1% MAF and sex discrepancies). Subsequently, we also conducted (4) pairwise identity by descent (IBD) analysis using PLINK software. Here, we only considered variants with MAF < 1% and a pruned dataset (linkage disequilibrium based pruning with R2 < 0.2). We further only kept patients with a pairwise PI_HAT ≤ 0.2. We then combined the data with the individuals from the 10000 Genomes Project and reduced the dataset to overlapping variants. By applying multidimensional scaling with PLINK and visually investigating the results of the first 2 components, we discovered and removed three ethnic outliers. Furthermore, we removed variants with a call rate < 98% or Hardy Weinberg p-value (pHWE) $<10^{-4}$.

We combined QCed data from both array types and applied a checking tool (http://www.well.ox.ac.uk/~wrayner/tools/; v4.2.7) to detect variants to be removed due to diverging strand, position and allele frequency information compared to the Haplotype Reference Consortium (HRC) panel. Subsequently, we performed imputation applying the Michigan imputation server (Das et al., 2016) (https://imputationserver.sph.umich.edu/index.html) with Eagle2 (v2.3) phasing and using the HRC panel(v1.1 2016) as reference. Finally, a post-imputation tool was applied (http://www.well.ox.ac.uk/~wrayner/tools/Post-Imputation.html; v1.0.2) to detect and remove genetic variants with unreliable imputation measures (R2 < 0.3, pHWE <1x10-4). In the end,

the considered genotype data comprised 10,249,108 autosomal variants for 98 patients for which methylation data is available.

Sample stratification using multivariate modelling
To investigate differences between tissues on a global level, we used DNA methylation data (including 98, 90 and 78 samples from low-grade and high-grade osteoarthritis cartilage and synovium, respectively) and removed batch effects using the ComBat function (Johnson et al., 2007) from the R package sva and considered these corrected methylation values for the multivariate models. We applied (1) principal component analysis (R function prcomp) and (2) a follow-up hierarchical clustering approach. To calculate the association between the second principal component (*PC2*) and cartilage grades, we built the following linear model: *PC2 ~ cartilage_type*.

For the hierarchical clustering approach, we applied the HCPC function from the R package FactoMineR (parameter settings: metric = "euclidean", method="ward") on the principal components (Lê et al., 2008).

Differential methylation analysis (discovery)
We sought to discover differentially methylated sites (DMSs) in pairs of low-grade and high-grade osteoarthritis cartilage samples from 90 patients. We performed linear modeling using the function lmFit and eBayes function of limma (Ritchie et al., 2015). We added the factor variable patient ID to ensure paired analysis design and additional 18 surrogate variables (SV) to account for technical confounders as covariates. The SV were estimated using the sva package by protecting the outcome variable tissue_state to conserve variation between low-grade osteoarthritis and high-grade osteoarthritis cartilage samples (Leek et al., 2012). The number of relevant SVs were estimated with the num.sv function (‘be’ method). To assess genome-wide significance in the EWAS, we applied Bonferroni correction considering the number of tested methylation sites: $0.05 / 401{,}870 = 1.24\times10^{-7}$. The standard errors of the coefficients were estimated by multiplying the unscaled standard deviations of the coefficients (“stdev.unscaled”) with the standard deviations of residual variances after Bayes shrinkage (“s2.post”).
To quantify the overrepresentation of hypermethylated sites among DMSs, we performed a binomial test (Number of successes: 8,783, number of trails: 15,328, expected probability of success: 0.5, alternative hypothesis: “greater”).
To identify differentially methylated regions (DMRs), we applied the R package dmrff (Suderman et al., 2018) to the summary statistics of the EWAS including the beta coefficients, their respective standard errors and p values. We applied the default parameter setting for the dmrff function (maxgap = 500, p.cutoff = 0.05). Regions were defined as differentially methylated when being composed of more than 1 methylation site and achieving a Bonferroni adjusted $p < 0.05$.
To identify sex-specific markers of cartilage degeneration, we performed EWAS in paired low-grade and high-grade cartilage samples of female (n = 52) and male (n = 38) patients, separately. We applied the same approach as in the combined EWAS to identify DMS (number of SVs: 14 and 10 in female and male-specific EWAS, respectively).

Differential methylation analysis (replication)
We performed an EWAS on knee samples of the replication data (17 low-grade and high-grade osteoarthritis cartilage samples, respectively) to validate our findings. To determine DMSs between low- and high-grade osteoarthritis cartilage, we applied a mixed-effect model (using the R package *lme4*):

$$methyl\_site = tissue\_status + gender + age + slide + 2\ SVs + (1|ID)$$

with *methyl_site* denoting m-values of a specific methylation site and *tissue_status* discriminating between low-grade osteoarthritis and high-grade osteoarthritis cartilage. We

also included 2 surrogate variables (the number was estimated using the *leek* method in the num.sv function of the sva R package) and *slide* as fixed effects to correct for technical variation, whereas *(1|ID)* accounts for patient-specific variation as random effect. We applied this specific model because a similar approach has been applied to this dataset previously (den Hollander et al., 2015).

Replicated DMSs are defined as (1) showing the same direction of effect in the replication set (2) at nominal significance ($p < 0.05$). We calculated the pvalues using the the Kenward-Roger approximation. To test whether there is an overrepresentation of hypomethylated sites among replicated DMSs, we performed a binomial test (Number of successes: 3,961, number of trails: 7,192, expected probability of success: 0.5, alternative hypothesis: “greater”).

We performed the EWAS on a regional level in the replication dataset with dmrff (default settings analog to the discovery analysis) by including summary statistics of the replication EWAS on methylation site level. We regard DMRs as replicated when they are composed of exactly the same sites in the replication set and show the same direction of effect on nominal significance ($p < 0.05$).

Pathway enrichment analysis

We applied the gometh and goregion functions (R package missMethyl) to identify enrichments among DMSs and DMRs (Maksimovic et al., 2021; Phipson et al., 2016). Across all approaches, we used Illumina’s manifest file for the 450k array (version 1.2) to annotate the methylation probes (parameter “anno”). We only considered pathways consisting of between 20 and 200 genes. To identify enrichments among the DMS in the discovery analysis, we used the 15,328 DMS as query (“sig.cpg”) and 401,870 methylation sites that passed the QC as background set. To investigate enrichments among replicated DMS, we used 7,192 replicated DMS as query and 346,288 methylation sites that passed the QC in the discovery and the replication set as background set. To examine enrichments among DMRs, we used 2,477 DMRs as query and 401,870 methylation sites that passed the QC in the discovery as background set.

Distinguishing cartilage grades using ML

To correct for technical variation in the methylation data (considering 98, 90 and 78 low-grade and high-grade osteoarthritis cartilage as well as synovium), we applied sva and estimated 25 relevant surrogate variables (‘be’ method) which we regressed out. We retained methylation samples from low-grade and high-grade osteoarthritis cartilage and standardized methylation values per site to reduce the influence of methylation site-specific variance.
Following up, we constructed classifiers that distinguish cartilage grades. We then trained and tested Random Forest (RF)-based classifiers repeatedly in 5-fold cross-validations (cv) in 25 iterations. Thus, we trained and tested 125 RF models (25 iterations * 5- fold cv) in total.

Per iteration, we divided the data randomly into five subsets, each comprising the same number of samples. Per fold, we then trained a Random Forest classifier on four subsets (= training set) and tested it on the hold-out set (= test set). To train one classifier, we conducted a two-step procedure:

(1) In the training data, we performed an EWAS using linear modelling (R function lmfit from the limma package) to assess the association between each methylation site and tissue status:

methylation ~ tissue_status

Of the differentially methylated sites, we selected 1000 sites with the largest absolute effect size ($p < 0.05$/number of tested methylation sites). Expressly, we only used the training set here to avoid overfitting.

(2) We built Random Forest-based classifiers considering the set of preselected methylation sites from the previous step. Here, we used the trainControl function of the caret R package (parameter setting: method = "repeatedcv", number = 3, repeats = 3) to define the training procedure. To build the actual model, we used the train function (method = ‘rf’).

We tested the resulting classifier on the hold-out test set by comparing the actual and predicted degradation state (low-grade or high-grade osteoarthritis cartilage) and estimated their respective prediction accuracy.
Across all iterations and folds, we trained and tested 125 classifiers, in total. We then reported the mean (μ) and standard deviation (σ) of the respective accuracy values (n = 125).
We further calculated the 95% Confidence interval (95% CI) of these accuracies with:

$$95\%\ CI = \mu \pm 1.96\ \frac{\sigma}{\sqrt{125}}$$

In addition, we calculated the receiver operating characteristic (ROC)-curve (based on the classifier’s classification probabilities) and estimated its corresponding area under the curve (AUC).

Validating ML classifiers

We sought to further investigate the performance of ML classifiers more generally by training one RF-based classifier on our entire dataset (i.e. not to sample subsets as in cross-validation) and then validating this model in an external dataset. We trained the classifier on the entire patient cohort by applying the same two-step procedure as during cross-validation (First: Performing EWAS to select 1000 methylation sites, second: Constructing the RF model). Subsequently, we tested the prediction quality of the resulting classifiers on the validation dataset which we standardized per methylation site (analog to the data preparation in the discovery analysis). We then applied the classifiers and assessed their prediction quality separately in hip and knee samples. Prediction accuracies and their 95% confidence intervals were calculated with carets ConfusionMatrix-function.
We applied the same training-testing strategy to construct a classifier based on support vector machines (SVM; caret’s train function method parameter: ‘svmLinear2’) and gradient boosting machine (gbm; caret’s train function method parameter: ‘gbm’) to compare the performances of these machine learning models on the validation set. Furthermore, we prioritized 300 methylation sites based on the variable importance (mean decrease in node impurity) in the RF model and overlapped them with DMSs.

Identification of methylation quantitative trait loci

We performed genome-wide cis-methylation quantitative trait locus (mQTL) analysis in low-grade (97 samples), high-grade osteoarthritis cartilage (89 samples) as well as in synovium (78 samples), thus including only samples for which complete covariate information was available. We restricted our analyses to SNPs with a minor allele frequency > 0.05 (low-grade osteoarthritis cartilage: 5,382,160 SNPs, high-grade osteoarthritis cartilage: 5,418,639 SNPs, synovium: 5,407,053 SNPs). Furthermore, we defined the cis-distance with 1 Mb. We used the R package vcfR (Knaus and Grünwald, 2017) to read the genotype files and extract allele dosages. We conducted the mQTL analysis using the R package MatrixEQTL (Shabalin, 2012). We estimated the mQTL effects with linear models:

methylation = genotype + age + sex + sequencing_batch

Methylation and *genotype* denoting m-values of methylation sites and the allele dosage values of genetic variants (a continuous value ranging between 0 and 2), respectively. We also included sex, age and sequencing batch information of each sample (*sequencing_batch*) to

correct for biological and technical variation in DNA methylation data. We defined two thresholds to identify genome-wide significant methylation QTL effects:

(1) Bonferroni threshold: Genome-wide significance defined by $p < 0.05$/number of tested SNP-methylation site pairs (Low-grade osteoarthritis cartilage: $p < 3.05 \times 10^{-11}$, high-grade osteoarthritis cartilage: $p < 3.03 \times 10^{-11}$, synovium: $p < 3.03 \times 10^{-11}$).
(2) False-discovery rate (FDR): We estimated the FDR of mQTL-effects using the MatrixEQTL package. It calculates the FDR considering the total number of tested cis-pairs per tissue.

Identification of sex-specific methylation quantitative trait loci

We performed cis-mQTL analysis (cis-distance: 1Mb) to identify sex-specific associations between methylation sites and SNPs per tissue (low-grade osteoarthritis cartilage: samples of 42 male and 55 female patients; high-grade osteoarthritis cartilage: 38 and 51; synovium: 29 and 49).

We considered SNPs with a MAF > 0.05 in samples of both sexes (SNP number in low-grade osteoarthritis cartilage: 4,927,666, high-grade osteoarthritis cartilage: 4,950,062, synovium: 5,062,551). Using the R package MatrixEQTL (Shabalin, 2012), we applied the following interaction model:

$$\text{methylation} = \text{age} + \text{sequencing\_batch} + \text{sex} * \text{genotype}$$

Methylation and *genotype* are denoting m-values of methylation sites and the allele dosage values, respectively. The term *sex * genotype* refers to the interaction term to test for the equality of the genetic effect between samples of the two genders.

Characterizing the mQTL architecture in osteoarthritis tissues

To characterize the mQTL targeted methylation sites in low-grade (n = 10,639) and high-grade (n = 6,785) osteoarthritis cartilage as well as in synovium (n = 4,493), we used annotations of Illumina's annotation file (version 1.2). For the enrichments approaches, we used methylation sites that (1) passed QC and (2) are within 1Mb to a SNP (thus, can be potentially be targeted by a cis-mQTL effect) as background set (n = 401,844). We performed the actual enrichment by applying hypergeometric tests (R function phyper). We applied the Bonferroni correction (location in genes: $p < 0.05/7$ categories = 0.0071; relation to CpG island: $p < 0.05/6$ categories = 0.0083)

Differential mQTL effects in low-grade and high-grade osteoarthritis cartilage

To calculate differential mQTL-effects between low-grade and high-grade osteoarthritis cartilage, we used the software MetaTissue v0.5 (Sul et al., 2013), available under http://genetics.cs.ucla.edu/metatissue/download.html. Analogously to our genome-wide, tissue-specific approach to identify mQTLs, we included sex, age and sequencing_batch as covariates in these models. We used the MetaTissue software to calculate posterior probabilities (m-values) and focused on genetic variant-methylation site pairs with a significant effect in one tissue (m-value > 0.9), but not in the other (m-value < 0.1). We only considered variant-methylation site pairs with a genome-wide significant effect in either low-grade or high-grade osteoarthritis cartilage (Bonferroni correction).

Comparing joint methylation QTLs with a whole blood based meta-study

We compared the effects of significant mQTL associations (Bonferroni correction) that were estimated in osteoarthritis-relevant tissues (low-grade osteoarthritis cartilage, high-grade osteoarthritis cartilage and synovium) with the corresponding effects (mQTL effect between the same variant-methylation site pairs) of a mQTL meta-analysis of 36 cohorts in whole blood**.** From the whole blood mQTL meta-analysis, we considered results from the fixed effect models.

Summary statistics of genome-wide association studies

For the MR approach and the colocalisation analysis, we included summary statistics from three osteoarthritis-related phenotypes: (1) osteoarthritis at any site (all OA) and (2) knee osteoarthritis (knee OA) and (3) total knee replacement (TKR). Summary statistics for all OA and knee OA were previously published (Tachmazidou et al., 2019) and downloaded from the gwas catalog. Summary statistics for TKR were calculated by meta-analysing the arcOGEN and UKBB data using the METAL software (Willer et al., 2010).

Two-sample Mendelian randomization (2SMR)

To estimate putative causal effects of methylation on osteoarthritis in each of the three examined joint tissues (low-grade and high-grade osteoarthritis cartilage as well as for synovium), we applied 2SMR by integrating mQTL and GWAS data from three osteoarthritis traits (all OA, knee OA, TKR).

We performed 2SMR following the workflow implemented in the R package TwoSampleMR (version 0.4.25) (Hemani et al., 2018). We first prioritized 15,328 methylation sites with significantly different methylation levels between low-grade and high-grade osteoarthritis cartilage. For these methylation sites, we selected methylation site-variant pairs with a significant cis-mQTL association (FDR < 0.05). We then converted the available location-based SNP ids (*chr:pos_allele1_allele2*) to rs-ids using information from dbSNP (version 151). Subsequently, we performed clumping ($r2 < 0.01$) to filter for independent mQTLs per methylation site. For this step, we applied the function clump_data of the TwoSampleMR package and used the LD structure of the European population of the 1000 genomes project. We then extracted the associations of the relevant mQTL SNPs from the GWAS summary statistics for three outcome traits (all OA, knee OA, TKR).

To estimate the causal effect of hypermethylation at a specific site on osteoarthritis and thus simplify the interpretation of the MR results, we orientated the beta-coefficient of the mQTL association between a SNP and methylation site to a positive effect. Subsequently, we harmonized the data with the function dat_harmonize.

We then conducted MR by integrating methylation sites (= exposure), osteoarthritis relevant outcomes (= outcome) and mQTL (instrumental variables = IV). For methylation sites with exactly 1 IV, we applied the Wald-ratio, otherwise the inverse-variance-weighted (IVW) method.

In low-grade osteoarthritis cartilage, we tested 3,378 methylation sites for their putative causal effect on osteoarthritis (all OA = 3,378 methylation sites, knee OA = 3,378, TKR = 3,343). In high-grade osteoarthritis cartilage, we considered 2,042 methylation sites (all OA = 2,042, knee OA = 2,042, TKR = 2,026). In synovium, we investigated the effect of 1,561 methylation sites (all OA = 1,560, knee OA = 1,560, TKR = 1,542). In total, we tested 10,099, 6,110 and 4,662 methylation site-osteoarthritis trait combinations in low-grade, high-grade osteoarthritis cartilage and synovium, respectively. Per tissue, we used the Bonferroni method (0.05 / total number of tested methylation site-GWAS trait pairs) to correct for the number of conducted tests (low-grade osteoarthritis cartilage: $p < 4.95 \times 10^{-6}$, high-grade osteoarthritis cartilage: $p < 8.18 \times 10^{-6}$, synovium: $p < 1.07 \times 10^{-5}$).

We investigated the opposite direction of effect (osteoarthritis causal for methylation changes) for every tested methylation site-osteoarthritis trait combination, including osteoarthritis trait as exposure and methylation site as outcome into the MR approach. We used independent GWAS risk SNPs as IV. If a risk SNP was not directly available, we included a proxy SNP (with $r^2 > 0.8$) which we estimated based on the LD structure from the UK Biobank. In summary, we applied 27, 10 and 4 SNPs as IV for all OA, knee OA and TKR, respectively. We applied the IVW method.

Colocalisation analysis

We applied colocalisation analysis to estimate the overlap of the mQTL signals and GWAS osteoarthritis signals (Giambartolomei et al., 2014). We examined genome-wide signals for osteoarthritis at any site (all OA, 33 risk loci), knee osteoarthritis (knee OA, 12 risk loci) and

total knee replacement (TKR, 5 risk loci*)* using colocalisation. Statistically independent signals for all OA and knee OA were previously reported (Tachmazidou et al., 2019). To estimate independent signals for TKR, we performed clumping (--clump-kb: 1000, --clump-r2 0.1) on 31 genome-wide significant variants ($p < 5e-8$) with the PLINK software (version 1.9), here considering the LD structure from the UK Biobank. With this approach, we found five independent signals. We performed colocalisation by applying coloc.fast function (https://github.com/tobyjohnson/gtx/blob/526120435bb3e29c39fc71604eee03a371ec3753/R/coloc.R) using default settings. We conducted the colocalisation analysis separately for each GWAS phenotype and each tissue. To define genomic regions to be tested for colocalisation, we prioritized methylation loci with (1) at least one significant cis-mQTL (FDR < 0.05) and which are in close vicinity (<500 kb distance) to the index variant of a GWAS signal. We then performed colocalisation in the genomic region that is 1Mb upstream and downstream of the methylation site which corresponds to the region in which the cis mQTL analysis was performed. We considered genetic variants present in both the cis mQTL and the GWAS data. As in a previous study in which signals of GWAS for osteoarthritis were overlapped with eQTL results from osteoarthritis-affected tissues (Steinberg et al., 2021), we applied a posterior probability for a shared causal variant (PP4) of >=80% as a threshold indicating colocalisation. Annotated genes and locations of colocalised GWAS signals were extracted from Ensembl Variant Effect Predictor (http://grch37.ensembl.org/Homo_sapiens/Tools/VEP/).

Combining colocalisation results with eQTL data

We combined these colocalisation results with previously estimated eQTL data from the same patient cohort (Steinberg et al., 2021). More specifically, we tested whether the lead SNP of colocalised GWAS OA signals show an eQTL effect on relevant genes at nominal significance ($p < 0.05$). Here, relevant genes (1) are either directly annotated or (2) have a transcription start site within 20 kb to a methylation site for which *cis*-mQTLs colocalised with the GWAS risk locus.

We further tested whether there are associations between a methylation site and gene of the same region when both were linked to a GWAS risk locus (methylation site by colocalising mQTLs, gene by an eQTL effect of the GWAS lead SNP). We used previously published expression data of the same patient cohort in the same tissue types (low-grade osteoarthritis cartilage: 75 patients, high-grade osteoarthritis cartilage: 76, synovium: 70) (Steinberg et al., 2021). We applied the following linear models:

gene = methylation_site + sex + age + 15 peer_factors + seqbatches

with *gene* and *methylation_site* denoting gene expression values and m-values, respectively. *Peer_factors* refer to peer factors that were estimated in gene expression profiles in the respective tissue. *Seqbatches* denote the sequencing batches of the methylation samples.
For methylation site-gene pairs (four pairs and one pair in low-grade and high-grade osteoarthritis cartilage, respectively) for which we found an association at nominal significance ($p < 0.05$), we applied one sample MR to test for causal effects of methylation on gene expression levels (exposure: methylation, outcome: gene expression, instrument: mQTL) using the R package ivreg. Analog to the association analysis, we included sex, age, methylation sequencing batches and 15 gene expression PEER factors as covariates into these models.

Comparative analysis of colocalisation in joint and blood

We tested whether osteoarthritis-risk variant-methylation site pairs that colocalise using joint mQTL data also colocalise when overlapping osteoarthritis GWAS with whole blood mQTL data. For this colocalisation approach, we applied the same colocalisation method as performed on joint mQTL data. We applied a threshold of PP4 >=80% indicating colocalisation and a threshold of PP4 < 20% indicating no colocalisation.

To further increase the comparability between joint- and whole blood-based results, we performed a follow-up sensitivity analysis by conducting colocalisation between joint-mQTL and GWAS osteoarthritis signals on a reduced set of variants that are present in blood and joint mQTL data as well as in the relevant GWAS data. Using strict filtering, we then regarded colocalising GWAS and mQTL signals to be joint tissue-specific when achieving a PP4 >=80% in joint mQTL data on the (1) largest possible as well as on the (2) reduced variant set and when (3) achieving a PP4 < 20% when performing colocalisation between GWAS signals and blood mQTL data.

Supplemental references

Alvarez-Garcia, O., Fisch, K.M., Wineinger, N.E., Akagi, R., Saito, M., Sasho, T., Su, A.I., and Lotz, M.K. (2016). Increased DNA Methylation and Reduced Expression of Transcription Factors in Human Osteoarthritis Cartilage. Arthritis & Rheumatology *68*, 1876–1886. https://doi.org/10.1002/art.39643.

Aryee, M.J., Jaffe, A.E., Corrada-Bravo, H., Ladd-Acosta, C., Feinberg, A.P., Hansen, K.D., and Irizarry, R.A. (2014). Minfi: a flexible and comprehensive Bioconductor package for the analysis of Infinium DNA methylation microarrays. Bioinformatics *30*, 1363–1369. https://doi.org/10.1093/bioinformatics/btu049.

Aubourg, G., Rice, S.J., Bruce-Wootton, P., and Loughlin, J. (2021). Genetics of osteoarthritis. Osteoarthritis and Cartilage https://doi.org/10.1016/j.joca.2021.03.002.

Boer, C.G., Yau, M.S., Rice, S.J., Coutinho de Almeida, R., Cheung, K., Styrkarsdottir, U., Southam, L., Broer, L., Wilkinson, J.M., Uitterlinden, A.G., et al. (2021). Genome-wide association of phenotypes based on clustering patterns of hand osteoarthritis identify *WNT9A* as novel osteoarthritis gene. Ann Rheum Dis *80*, 367–375. https://doi.org/10.1136/annrheumdis-2020-217834.

Bonin, C.A., Lewallen, E.A., Baheti, S., Bradley, E.W., Stuart, M.J., Berry, D.J., van Wijnen, A.J., and Westendorf, J.J. (2016). Identification of Differentially Methylated Regions in New Genes Associated with Knee Osteoarthritis. Gene *576*, 312–318. https://doi.org/10.1016/j.gene.2015.10.037.

Chen, Y., Lemire, M., Choufani, S., Butcher, D.T., Grafodatskaya, D., Zanke, B.W., Gallinger, S., Hudson, T.J., and Weksberg, R. (2013). Discovery of cross-reactive probes and polymorphic CpGs in the Illumina Infinium HumanMethylation450 microarray. Epigenetics *8*, 203–209. https://doi.org/10.4161/epi.23470.

Das, S., Forer, L., Schönherr, S., Sidore, C., Locke, A.E., Kwong, A., Vrieze, S.I., Chew, E.Y., Levy, S., McGue, M., et al. (2016). Next-generation genotype imputation service and methods. Nat Genet *48*, 1284–1287. https://doi.org/10.1038/ng.3656.

Du, P., Zhang, X., Huang, C.-C., Jafari, N., Kibbe, W.A., Hou, L., and Lin, S.M. (2010). Comparison of Beta-value and M-value methods for quantifying methylation levels by microarray analysis. BMC Bioinformatics *11*, 587. https://doi.org/10.1186/1471-2105-11-587.

Durinck, S., Spellman, P.T., Birney, E., and Huber, W. (2009). Mapping identifiers for the integration of genomic datasets with the R/Bioconductor package biomaRt. Nat Protoc *4*, 1184–1191. https://doi.org/10.1038/nprot.2009.97.

Eckhardt, F., Lewin, J., Cortese, R., Rakyan, V.K., Attwood, J., Burger, M., Burton, J., Cox, T.V., Davies, R., Down, T.A., et al. (2006). DNA methylation profiling of human chromosomes 6, 20 and 22. Nat Genet *38*, 1378–1385. https://doi.org/10.1038/ng1909.

Edgar, R., Domrachev, M., and Lash, A.E. (2002). Gene Expression Omnibus: NCBI gene expression and hybridization array data repository. Nucleic Acids Res *30*, 207–210. https://doi.org/10.1093/nar/30.1.207.
Fortin, J.-P., Labbe, A., Lemire, M., Zanke, B.W., Hudson, T.J., Fertig, E.J., Greenwood, C.M., and Hansen, K.D. (2014). Functional normalization of 450k methylation array data improves replication in large cancer studies. Genome Biol *15*, 503. https://doi.org/10.1186/s13059-014-0503-2.
Fortin, J.-P., Triche, T.J., and Hansen, K.D. (2017). Preprocessing, normalization and integration of the Illumina HumanMethylationEPIC array with minfi. Bioinformatics *33*, 558–560. https://doi.org/10.1093/bioinformatics/btw691.
Giambartolomei, C., Vukcevic, D., Schadt, E.E., Franke, L., Hingorani, A.D., Wallace, C., and Plagnol, V. (2014). Bayesian test for colocalisation between pairs of genetic association studies using summary statistics. PLoS Genet *10*, e1004383. https://doi.org/10.1371/journal.pgen.1004383.
Hemani, G., Zheng, J., Elsworth, B., Wade, K.H., Haberland, V., Baird, D., Laurin, C., Burgess, S., Bowden, J., Langdon, R., et al. (2018). The MR-Base platform supports systematic causal inference across the human phenome. ELife *7*, e34408. https://doi.org/10.7554/eLife.34408.
den Hollander, W., Ramos, Y.F.M., Bos, S.D., Bomer, N., van der Breggen, R., Lakenberg, N., de Dijcker, W.J., Duijnisveld, B.J., Slagboom, P.E., Nelissen, R.G.H.H., et al. (2014). Knee and hip articular cartilage have distinct epigenomic landscapes: implications for future cartilage regeneration approaches. Ann Rheum Dis *73*, 2208–2212. https://doi.org/10.1136/annrheumdis-2014-205980.
den Hollander, W., Ramos, Y.F.M., Bomer, N., Elzinga, S., van der Breggen, R., Lakenberg, N., de Dijcker, W.J., Suchiman, H.E.D., Duijnisveld, B.J., Houwing-Duistermaat, J.J., et al. (2015). Transcriptional associations of osteoarthritis-mediated loss of epigenetic control in articular cartilage. Arthritis Rheumatol *67*, 2108–2116. https://doi.org/10.1002/art.39162.
Johnson, W.E., Li, C., and Rabinovic, A. (2007). Adjusting batch effects in microarray expression data using empirical Bayes methods. Biostatistics *8*, 118–127. https://doi.org/10.1093/biostatistics/kxj037.
Knaus, B.J., and Grünwald, N.J. (2017). vcfr: a package to manipulate and visualize variant call format data in R. Mol Ecol Resour *17*, 44–53. https://doi.org/10.1111/1755-0998.12549.
Lê, S., Josse, J., and Husson, F. (2008). FactoMineR: An R Package for Multivariate Analysis. Journal of Statistical Software *25*, 1–18. https://doi.org/10.18637/jss.v025.i01.
Leek, J.T., Johnson, W.E., Parker, H.S., Jaffe, A.E., and Storey, J.D. (2012). The sva package for removing batch effects and other unwanted variation in high-throughput experiments. Bioinformatics *28*, 882–883. https://doi.org/10.1093/bioinformatics/bts034.
Maksimovic, J., Oshlack, A., and Phipson, B. (2021). Gene set enrichment analysis for genome-wide DNA methylation data. Genome Biology *22*, 173. https://doi.org/10.1186/s13059-021-02388-x.
McCartney, D.L., Walker, R.M., Morris, S.W., McIntosh, A.M., Porteous, D.J., and Evans, K.L. (2016). Identification of polymorphic and off-target probe binding sites on the Illumina Infinium MethylationEPIC BeadChip. Genom Data *9*, 22–24. https://doi.org/10.1016/j.gdata.2016.05.012.
Min, J.L., Hemani, G., Davey Smith, G., Relton, C., and Suderman, M. (2018). Meffil: efficient normalization and analysis of very large DNA methylation datasets. Bioinformatics *34*, 3983–3989. https://doi.org/10.1093/bioinformatics/bty476.

Min, J.L., Hemani, G., Hannon, E., Dekkers, K.F., Castillo-Fernandez, J., Luijk, R., Carnero-Montoro, E., Lawson, D.J., Burrows, K., Suderman, M., et al. (2021). Genomic and phenotypic insights from an atlas of genetic effects on DNA methylation. Nat Genet *53*, 1311–1321. https://doi.org/10.1038/s41588-021-00923-x.
Phipson, B., Maksimovic, J., and Oshlack, A. (2016). missMethyl: an R package for analyzing data from Illumina's HumanMethylation450 platform. Bioinformatics *32*, 286–288. https://doi.org/10.1093/bioinformatics/btv560.
Pidsley, R., Zotenko, E., Peters, T.J., Lawrence, M.G., Risbridger, G.P., Molloy, P., Van Djik, S., Muhlhausler, B., Stirzaker, C., and Clark, S.J. (2016). Critical evaluation of the Illumina MethylationEPIC BeadChip microarray for whole-genome DNA methylation profiling. Genome Biol *17*, 208. https://doi.org/10.1186/s13059-016-1066-1.
Ritchie, M.E., Phipson, B., Wu, D., Hu, Y., Law, C.W., Shi, W., and Smyth, G.K. (2015). limma powers differential expression analyses for RNA-sequencing and microarray studies. Nucleic Acids Res *43*, e47. https://doi.org/10.1093/nar/gkv007.
Rushton, M.D., Reynard, L.N., Barter, M.J., Refaie, R., Rankin, K.S., Young, D.A., and Loughlin, J. (2014). Characterization of the Cartilage DNA Methylome in Knee and Hip Osteoarthritis. Arthritis & Rheumatology *66*, 2450–2460. https://doi.org/10.1002/art.38713.
Shabalin, A.A. (2012). Matrix eQTL: ultra fast eQTL analysis via large matrix operations. Bioinformatics *28*, 1353–1358. https://doi.org/10.1093/bioinformatics/bts163.
Steinberg, J., and Zeggini, E. (2016). Functional genomics in osteoarthritis: Past, present, and future. J Orthop Res *34*, 1105–1110. https://doi.org/10.1002/jor.23296.
Steinberg, J., Ritchie, G.R.S., Roumeliotis, T.I., Jayasuriya, R.L., Clark, M.J., Brooks, R.A., Binch, A.L.A., Shah, K.M., Coyle, R., Pardo, M., et al. (2017). Integrative epigenomics, transcriptomics and proteomics of patient chondrocytes reveal genes and pathways involved in osteoarthritis. Sci Rep *7*, 8935. https://doi.org/10.1038/s41598-017-09335-6.
Steinberg, J., Southam, L., Roumeliotis, T.I., Clark, M.J., Jayasuriya, R.L., Swift, D., Shah, K.M., Butterfield, N.C., Brooks, R.A., McCaskie, A.W., et al. (2021). A molecular quantitative trait locus map for osteoarthritis. Nature Communications *12*, 1309. https://doi.org/10.1038/s41467-021-21593-7.
Suderman, M., Staley, J.R., French, R., Arathimos, R., Simpkin, A., and Tilling, K. (2018). dmrff: identifying differentially methylated regions efficiently with power and control. BioRxiv 508556. https://doi.org/10.1101/508556.
Sul, J.H., Han, B., Ye, C., Choi, T., and Eskin, E. (2013). Effectively identifying eQTLs from multiple tissues by combining mixed model and meta-analytic approaches. PLoS Genet *9*, e1003491. https://doi.org/10.1371/journal.pgen.1003491.
Tachmazidou, I., Hatzikotoulas, K., Southam, L., Esparza-Gordillo, J., Haberland, V., Zheng, J., Johnson, T., Koprulu, M., Zengini, E., Steinberg, J., et al. (2019). Identification of new therapeutic targets for osteoarthritis through genome-wide analyses of UK Biobank data. Nature Genetics *51*, 230–236. https://doi.org/10.1038/s41588-018-0327-1.
Willer, C.J., Li, Y., and Abecasis, G.R. (2010). METAL: fast and efficient meta-analysis of genomewide association scans. Bioinformatics *26*, 2190–2191. https://doi.org/10.1093/bioinformatics/btq340.
Zhang, Y., Fukui, N., Yahata, M., Katsuragawa, Y., Tashiro, T., Ikegawa, S., and Michael Lee, M.T. (2016). Genome-wide DNA methylation profile implicates potential cartilage regeneration at the late stage of knee osteoarthritis. Osteoarthritis and Cartilage *24*, 835–843. https://doi.org/10.1016/j.joca.2015.12.013.

Email addresses:
Peter Kreitmaier: peter.kreitmaier@helmholtz-muenchen.de
Matthew Suderman: matthew.suderman@bristol.ac.uk
Lorraine Southam: lorraine.southam@helmholtz-muenchen.de
Rodrigo Coutinho de Almeida: R.Coutinho_de_Almeida@lumc.nl
Konstantinos Hatzikotoulas: hatzikotoulas@helmholtz-muenchen.de
Ingrid Meulenbelt: i.meulenbelt@lumc.nl
Julia Steinberg: Julia.Steinberg@nswcc.org.au
Caroline Relton: Caroline.Relton@bristol.ac.uk
J Mark Wilkinson: j.m.wilkinson@sheffield.ac
Eleftheria Zeggini: eleftheria.zeggini@helmholtz-muenchen.de
